# N-Aryl Benzimidazole and Benzotriazole Derivatives and Their Hybrids as Cytotoxic Agents: Design, Synthesis and Structure–Activity Relationship Studies

**DOI:** 10.3390/molecules29225360

**Published:** 2024-11-14

**Authors:** Yulia R. Aleksandrova, Natalia S. Nikolaeva, Inna A. Shagina, Karina D. Smirnova, Alla A. Zubishina, Alexander I. Khlopotinin, Artem N. Fakhrutdinov, Alexander L. Khokhlov, Roman S. Begunov, Margarita E. Neganova

**Affiliations:** 1 Nesmeyanov Institute of Organoelement Compounds, Russian Academy of Sciences, Moscow 119991, Russia; aleksyu@ineos.ac.ru (Y.R.A.); schagina.in@yandex.ru (I.A.S.); 2 Institute of Physiologically Active Compounds, Russian Academy of Sciences, Chernogolovka 142432, Russia; nikolaevans@bk.ru; 3 Faculty of Biology and Ecology, P. G. Demidov Yaroslavl State University, Yaroslavl 150003, Russia; smirnovakard@gmail.com (K.D.S.); a.zubishina@uniyar.ac.ru (A.A.Z.); mouthleg547@gmail.com (A.I.K.); 4 N. D. Zelinsky Institute of Organic Chemistry, Russian Academy of Sciences, Moscow 119991, Russia; fak.part@gmail.com; 5 Institute of Pharmacy, Yaroslavl State Medical University of the Ministry of Health of the Russian Federation, Yaroslavl 150000, Russia; al460935@yandex.ru

**Keywords:** hybrid molecules, heterocycles, benzimidazoles, benzotriazoles, diphenylamines, anticancer agents, cytotoxicity, antioxidants, cell migration

## Abstract

The era of chemotherapy began in the 1940s, which is the basis of traditional antitumor approaches and, being one of the most high-tech treatment methods, is still widely used to treat various types of cancer. A promising direction in modern medicinal chemistry is currently the creation of hybrid molecules containing several pharmacophore fragments of different structures. This strategy is successfully used to increase the therapeutic efficacy of cytotoxic agents and reduce side effects. In this work, we synthesized 10 1-aryl derivatives of benzimidazole and benzotriazole and 11 hybrids based on them. Among the compounds obtained, the most promising hybrid molecules were diphenylamines, containing an amino group and a benzotriazole cycle in the *ortho* position to the bridging NH group, which showed significant cytotoxic activity, excellent antioxidant properties and the ability to suppress the migration activity of tumor cells. Taken together, our results demonstrate that substituted diphenylamine-based bipharmacophoric compounds may serve as a promising platform for further optimization to obtain effective antitumor compounds.

## 1. Introduction

At the present time, malignant tumors are the second most common cause of mortality from non-communicable diseases in many developed countries, second only to heart and blood vessel diseases [1]. However, according to the forecast of the World Health Organization, cancer may become the leading cause of death worldwide in the coming decades [2]. Over 35 million new cancer cases are predicted for 2050, a 77% increase from the estimated 20 million cases in 2022 [2]. In addition to being an important barrier to increasing life expectancy, cancer is associated with significant social and macroeconomic costs, which could amount to approximately USD 25 trillion by 2050 [3,4].

Despite significant progress in cancer treatment, the search for effective anti-tumor therapy approaches remains a top priority because of the many limitations of current treatment options.

In modern medicine, there are many treatment options for various cancers, depending on the stage of the malignant process. The most important among them, despite the existing shortcomings, is chemotherapy [5,6,7,8,9,10].

The main advantages of this type of treatment are its accessibility and high effectiveness. This is due to the wide variety of drugs available. These drugs can not only cure cancer but also control it, preventing cancer cells from spreading to the body and forming metastases. Chemotherapy is based on the use of cytostatic drugs, which can affect tumor cells and prevent their growth and reproduction. Typically, it is used in combination with surgery and/or radiation therapy, but it can also be used separately for cancer treatment. For many patients, chemotherapy is a key step in the fight against cancer, helping to increase their chances of recovery. 

Now, there is no alternative to chemotherapy for the treatment of several malignant tumors (in particular, with a high prevalence of the tumor process and a high probability of metastasis). Thus, chemotherapeutic agents have played an important role over the past several decades and remain the top choice for the treatment of late-stage malignant neoplasms when surgery and/or radiation therapy cannot be prescribed for certain reasons [5,7,8,9,10].

Thus, the development of new effective cytotoxic agents for the chemotherapy of oncological diseases represents an urgent need in healthcare.

Currently, a promising strategy for creating effective drugs, including antitumor drugs, is the development of hybrid molecules containing several pharmacophore fragments with different structures [11,12,13,14]. 

Often, the pharmacophores of such compounds are nitrogen-containing heterocycles, for example, benzimidazole, triazole and benzotriazole [15,16,17,18,19,20]. Their presence in a hybrid molecule with other pharmacophores enhances the biological activity of the resulting substance. Hybrids based on benzimidazole and triazole exhibit high antitumor activity against various cancer cell lines [21,22,23,24]. Examples of antitumor substances containing benzimidazole and benzotriazole fragments have not been reported. It can be expected that the simultaneous presence of these heterocycles in the molecule will provide a greater antitumor effect than the individual action of each heterocycle.

Another promising pharmacophore is the diphenylamine fragment [25,26,27,28,29]. This fragment is a component of biologically active medicinal drugs, such as diclofenac and tolfenamic acid. There is some structural similarity between the diphenylamine core and the DNA minor groove binders. Molecules that bind to genomic DNA and are easily accessible to chromosomal DNA have been proven to be effective anticancer therapeutic agents [30]. In addition, the literature shows that diphenylamine and a number of its derivatives have antioxidant effects [31,32,33,34], which indicates a wide range of biological properties.

**Figure 1:**
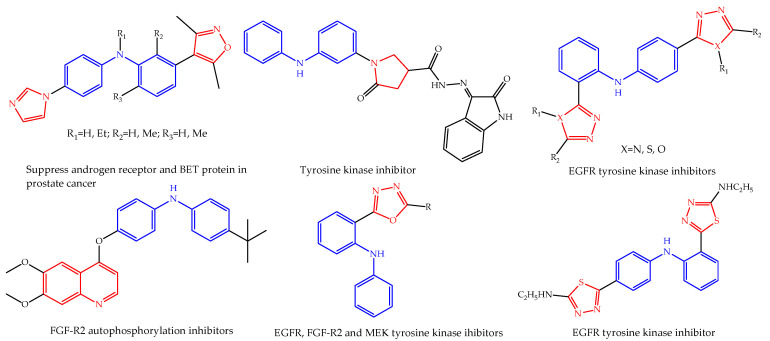
Some examples of azaheterocycle-containing diphenylamines with anticancer activity.

Many diphenylamine derivatives containing azaheterocycles exhibit antitumor activity (Figure 1) [35,36,37,38,39,40].

Thus, there is no doubt that the synthesis and study of the antitumor potential of bipharmacophore compounds based on condensed polyazaheterocycles and substituted diphenylamines can contribute to the development of new pharmaceuticals for cancer chemotherapy.

Therefore, in this study, we synthesized 11 new hybrid molecules based on 1-aryl derivatives of benzimidazole and benzotriazole. The goal was to identify substances with potential antitumor activity.

## 2. Results

### 2.1. Chemistry

**Figure 2:**
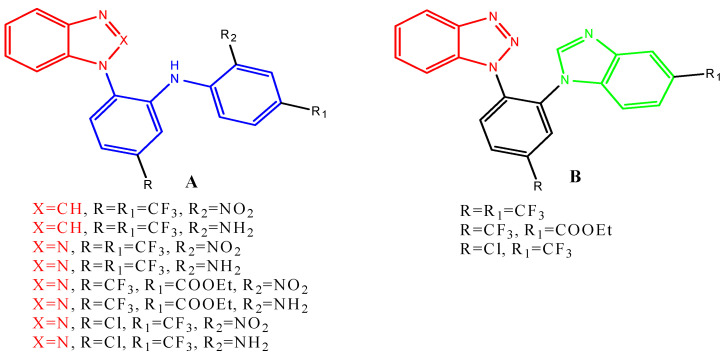
Chemical structures of synthesized hybrid molecules, which are active pharmaceutical ingredients. (**A**) Fused hybrid; (**B**) a hybrid with a linker.

This article describes the synthesis of two types of hybrid molecules (Figure 2): a fused hybrid (**A**) and a hybrid with a linker (**B**).

**Scheme 1:**
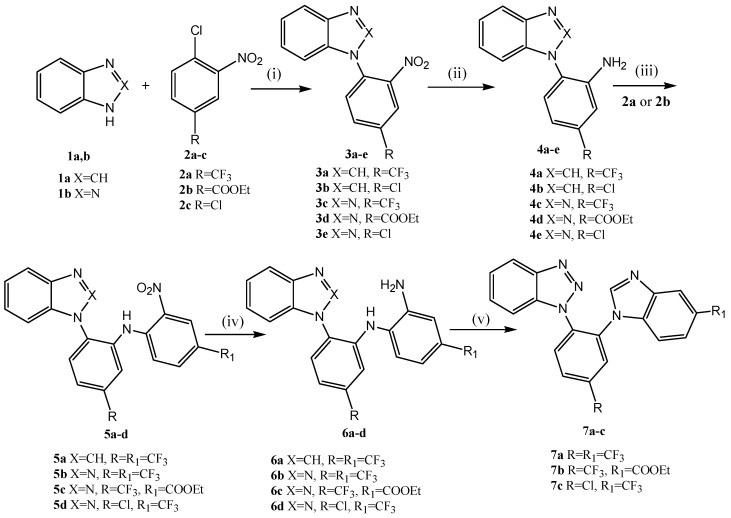
Synthesis of novel hybrid molecules **5a**–**d**, **6a**–**d** and **7a**–**c.** Reagents and conditions: (**i**) K_2_CO_3_, DMF, 110 °C for 2 h of **3a**, 3 h of **3c,d** or 7 h of **3b,e**; (**ii**) TiCl_3_, 10% HCl, *i*-PrOH, 70 °C, 5 min; (**iii**) **2a** or **2b**, K_2_CO_3_, DMF, 110 °C, 12 h; (**iv**) TiCl_3_, 10% HCl, *i*-PrOH, 70 °C, 5 min; (**v**) HCOOH, DMF, 1 h, 90 °C.

The synthesis of the bipharmacophore molecules **5a**–**d**, **6a**–**d** and **7a**–**c** was carried out according to Scheme 1.

*ortho*-Nitrohaloarenes were used as ligands. They contained substituents that enhance the biological activity of substances: a chlorine atom and trifluoromethyl and ethoxycarbonyl groups [41,42,43,44,45,46,47,48,49,50].

**Scheme 2:**
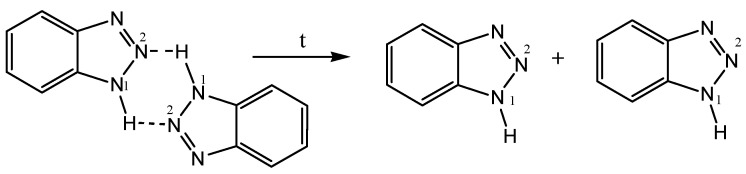
Destruction of hydrogen bonds in the putative benzotriazole dimer in DMF solution under the influence of temperature.

The presence of a halogen atom activated by electron acceptor groups in the **2a**–**c** ligand facilitated the introduction of the first pharmacophore, the benzimidazole or benzotriazole cycle in the S*_N_*Ar reaction. A prerequisite for the synthesis of 1-(2-nitrophenyl)-1*H*-benzotriazoles **3c**–**e** was to carry out the substitution process at a temperature of at least 110 °C. At lower temperatures, the formation of isomeric 2-(2-nitroaryl)-2*H*-benzotriazoles occurred. Apparently, at low temperature, benzotriazole molecules in DMFA solution are mainly in the form of dimers, in which a proton transition from the nitrogen atom N^1^ of one associate to the N^2^ of another is possible. The reaction center in such dimers can be an atom of both N^1^ and N^2^. When the temperature rises, the hydrogen bonds in the dimer are destroyed (Scheme 2) and one molecule of 1*H*-benzotriazole participates in the reaction.

The duration of the S*_N_*Ar process depended on the electronic nature of the R substituent in reagent **2**. At R = CF_3_ (**2a**) and COOEt (**2b**), a 100% conversion of was observed for **1a** after 2 h and for **1b** after 3 h. The reaction time increased to 7 h when using dihalogennitroarene, **2c**.

To form the 2nd pharmacophore, the nitro group in **3a**–**e** nitro derivatives was reduced to amino group. The reduction of the **3a**–**e** nitro compounds was carried out with titanium (III) chloride in an acidic aqueous alcohol medium at 70 °C for 5 min. Isopropanol was used as a solvent, in which the nitroarenes **3a**–**e** were dissolved by heating. Acetic acid was not used due to difficulties in separating the product. In other proton solvents, not all nitro compounds dissolved. After 5 min, the reaction mass was rapidly cooled and NH_4_OH was treated to pH 7–8. Amine product **4** was isolated by the extraction of the reaction mixture with chloroform. Compounds **4a**–**e** were obtained with high yields and subsequently used without additional purification.

**Scheme 3:**
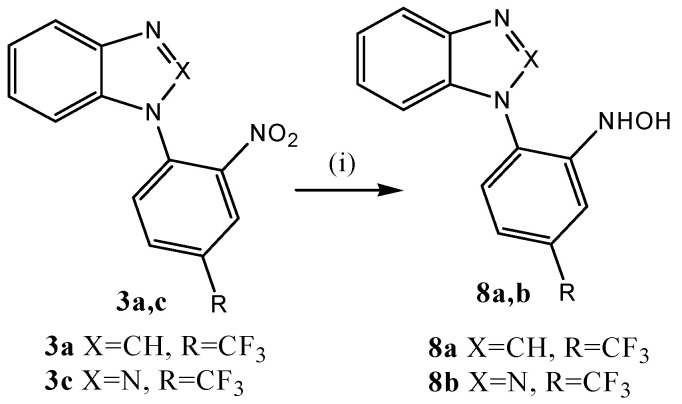
Synthesis of *N*-(2-(1*H*-benzimidazol-1-yl)-5-(trifluoromethyl)phenyl)hydroxylamine (**8a**) and *N*-(2-(1*H*-benzotriazol-1-yl)-5-(trifluoromethyl)phenyl)hydroxylamine (**8b**)**.** Reagents and conditions: (i) SnCl_2_∙2H_2_O, 18% HCl, *i*-PrOH, 70 °C, 5 min.

The use of SnCl_2_ for the reduction of nitro compounds **3** was undesirable. During the reaction, for 5 min, the reaction mass mainly contained the product of the incomplete reduction of the nitro group: arylhydroxylamine **8** (Scheme 3). An increase in the reduction process time could lead to the isomerization of the resulting amino products [51,52,53].

Some of the amino compounds **4** were used in the S*_N_*Ar reaction with *ortho*-nitrogalogenarenes **2**. Compounds **4** showed weak nucleophilic properties. Therefore, the substitution process was carried out for 12 h at 110 °C. As a result, heterocyclic derivatives of diphenylamine **5** were obtained: hybrid type **A** molecules containing an *ortho*-nitro group. The formation of a diphenylamine fragment was evidenced by the presence of an NH-group proton signal in the ^1^H NMR spectrum of the **5a**–**d** compounds, having the form of a narrow singlet, at 9.79–10.0 m.d.

To expand the structural diversity of the type **A** hybrids, the nitro group in compounds **5a**–**d** was reduced to amino group. The reduction was carried out similarly to the synthesis of amines **4**. The presence of an amino group in the *ortho* position contributed to the displacement of the proton signal of the NH group of diphenylamines **6a**–**d** into the high-field region of the ^1^H NMR spectrum 7.32–7.53 ppm.

The hybrid type **B** molecules were obtained by reacting **6a**–**d** with formic acid. The intermolecular heterocyclization reaction was carried out in DMF for 1 h at 90 °C. In the ^1^H NMR spectrum of the hybrid **7a**–**c** molecules, there were no signals of protons of the NH_2_ group in the region of 5.09–5.31 ppm and the NH group. The presence of a characteristic heteroaromatic proton signal at 8.52–8.61 ppm in the form of a narrow singlet indicated the successful formation of a benzimidazole cycle [51,52,53].

Thus, we obtained various compounds—fuse hybrids **5**, **6a**–**d** and hybrids with a linker **7a**–**c** and their biological activity was studied at the next stage of the work.

### 2.2. Biological Evaluation

#### 2.2.1. Cytotoxic Effect

At the first stage of biological testing, we studied the effect of the synthesized compounds on the survival of cells of both tumoral and normal origin. In order to assess the effect of the nature of the heterocycle and substituents in the hybrid molecule on the manifestation of toxic effects, we studied intermediate compounds **3a**–**e** and **4a**–**e**. It is well known that the presence of cytotoxic properties in candidates for antitumor drugs is a prerequisite for their further study as effective therapeutic agents. In our work, the cytotoxic profile of the synthesized molecules was assessed using the MTT test, based on the ability of a colorless tetrazolium salt (3-[4,5-dimethylthiazol-2-yl]-2,5-diphenyltetrazolium bromide, MTT) to be reduced to colored formazan in the presence of the mitochondrial enzymes of living cells. For this purpose, a number of cell lines of tumoral origin (SH-SY5Y, A549, MCF-7 and SW-480), as well as normal origin (Hek-293), were used. The results of this study are presented in Table 1 as the IC_50_ values of the cytotoxic effect.

Analysis of the cytotoxic profile of intermediates **3a**–**e** and **4a**–**e** showed that substances **3a**–**e** containing a nitro group were less toxic than their corresponding amino derivatives **4a**–**e**. Almost all nitro compounds, with the exception of **3a** which contained a benzimidazole ring and a trifluoromethyl group, did not cause cancer cell death in the range of concentrations studied. In turn, of the five amines **4a**–**e** studied, only three substances, **4a**, **4c**, **4e**, affected the survival of cell cultures. At the same time, the amino derivative **4c**, containing a benzotriazole ring and a trifluoromethyl group, had the most pronounced cytotoxic effect on neuroblastoma cells without affecting the survival of normal Hek-293 cells.

A similar effect was observed when considering the influence of the fused hybrid structures (type A) **5a**–**d** and **6a**–**d** on the survival of various cells of tumoral and normal origin. Thus, nitro derivatives **5a**–**d** did not have a toxic effect on all the tested cell lines. At the same time, the presence of an amino group in compounds **6b**–**d** led to a significant increase in cytotoxic properties. The exception was hybrid **6a**, containing a benzimidazole ring instead of a benzotriazole ring. It was not toxic to the studied cell lines. All the benzotriazole derivatives of diphenylamine **6b**–**d** had a more pronounced ability to suppress the survival of cells of tumoral origin compared to amino compounds **4c**–**e**.

Interestingly, depending on the nature of the substituents R and R1, a variation in the cytotoxic effect of hybrids **6b**–**d** was observed with respect to the studied cell lines. Thus, at R = R1 = CF_3_, compound **6b** suppressed the survival of all cells, including those of normal origin, Hek-293 (the IC_50_ values of the cytotoxic effect were in the range from 22 to 26 μM). A similar effect was also exerted by substance **6d** (R = Cl, R1 = CF_3_). However, the presence of a chlorine atom in its structure instead of a CF_3_ group led to a decrease in the cytotoxic effect (the IC_50_ values of the cytotoxic effect were in the range from 31 to 41 μM). Of particular interest were the data on the cytotoxicity of substance **6c** (R = CF_3_, R1 = COOEt), containing an ethoxycarbonyl group in the *ortho*-phenylenediamine fragment. Thus, **6c** did not exert a cytotoxic effect on a cell line of normal origin, leading to a significant decrease in the survival of SH-SY5Y and A549 cancer cells (with the IC_50_ values of the cytotoxic effect being 20.94 ± 0.13 and 37.68 ± 0.20, respectively). This is certainly considered a positive property of the substance, potentially indicating its ability to exhibit a selective effect on cells of tumoral origin without affecting healthy microenvironments.

In turn, in the series of hybrids with a linker (type B) **7a**–**c**, only the bipharmacophoric substance **7c** had a cytotoxic effect on the breast ductal adenocarcinoma MCF-7, while exhibiting more pronounced toxic properties in relation to cells of normal origin. Based on the data obtained in the study of the cytotoxic profile of the synthesized molecules, the most promising hybrid compounds were diphenylamines **6**, containing an amino group and a benzotriazole ring in the *ortho* position to the bridging NH group, and varying the substituents in these molecules made it possible to change the cytotoxic effect of the hybrids in relation to various types of cancer cells. Thus, at the next stages of this biological research, we were faced with the goal of clarifying the possible mechanisms of action of a number of diphenylamines, **6a**–**d,** synthesized as potential antitumor agents.

#### 2.2.2. Antioxidant Activity

It is well known that tumor cells generate a large number of reactive oxygen species (ROS) compared to normal cells due to the peculiarities of their metabolism [54]. Thus, playing the role of secondary messengers [55,56], ROS participate in various stages of tumor development, including oncogenesis [57,58,59], proliferation [60,61], angiogenesis [62,63], metastasis [64,65] and drug resistance [54,66]. Moreover, excessively high intracellular levels of ROS disrupt the structure of the lipids, proteins and DNA of healthy cells, triggering a cycle of genomic instability and aggravating the process of tumor transformation [67]. In this regard, the ability of potential antineoplastic agents to inhibit ROS formation is considered a promising property, and a large number of research groups aim to create molecules with such an action profile.

**Figure 3:**
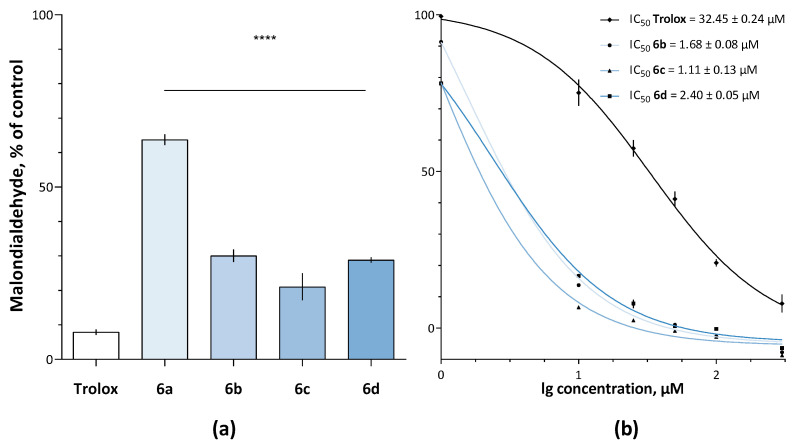
Effect of **6a**–**d** on the lipid peroxidation of rat brain homogenate (2 mg/mL) initiated by Fe(II)-500 μM. (**a**) Histograms reflecting the content of malondialdehyde in samples under the action of compounds at a concentration of 100 μM. (**b**) Curves of the “concentration–effect” dependence, reflecting the calculation of the values of IC_50_ inhibiting the lipid peroxidation effect. The concentration of the substances ranged from 1 to 300 μM. To assess the statistical significance, a one-sided ANOVA and the Dunnett multiple comparison test were used, where ****, *p* < 0.0001, when compared to a control taken as 100%.

In our work, the antioxidant status of the compounds was determined by their ability to influence the process of Fe(II)-induced lipid peroxidation (LPO) of rat brain homogenate (2 mg/mL). The effect of the substances on LPO was studied using a modified version of the TBA test in a plate format [68]. This test is based on the reaction of 2-thiobarbituric acid with intermediate LPO products, resulting in the formation of a colored trimethine complex, the main role in the formation of which belongs to malondialdehyde. As shown in Figure 3a, compounds **6b**, **c**, **d** effectively inhibited LPO initiated by ferrous iron ions. This was expressed in a decrease in the level of malondialdehyde in the system by 70, 79 and 71%, respectively, tending to the values of the well-known antioxidant Trolox. Additionally, the IC_50_ values of the lipid peroxidation inhibitory effect were calculated for these molecules, the values of which are presented in Figure 2B. Interestingly, the IC_50_ values of these compounds significantly exceeded those of the Trolox (19, 14 and 29 times, respectively), which indicates the ability of these substances to exhibit a more pronounced effect at much lower concentrations.

#### 2.2.3. Modulation of the Migration Ability and Antiproliferative Effect

It is also well known that migration and proliferation of cancer cells are key processes involved in the metastatic cascade of malignant neoplasms [69,70,71]. In turn, the ability of therapeutic agents to inhibit these processes is considered a promising property of antitumor agents. In this regard, in order to assess the possible antimetastatic potential of cytotoxic diphenylamines **6b**, **c** and **d**, we analyzed the effect of the substances on the migration and proliferation of tumor cells A549 in a scratch test. 

**Figure 4:**
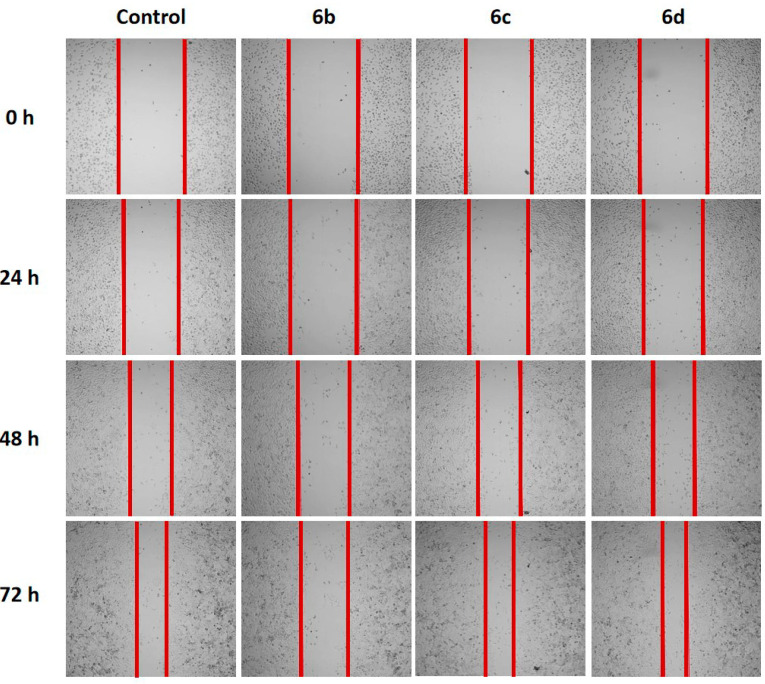
The ability of diphenylamines **6b**, **6c** and **6d** to modulate the migratory ability and proliferative potential of A549 cells, studied using the scratch test. Representative images of defect healing, taken immediately after the scratch was applied and 24, 48 and 72 h after cell treatment with the test substances (×40).

Based on a preliminary series of experiments, 1 μM was chosen as the concentration used in this test, excluding the intrinsic cytotoxic effect of the substances. Figure 4 shows the results of the study of the ability of **6b**, **c** and **d** to suppress the migration and proliferation of the A549 cells. Thus, the data obtained 24 h after the damage was applied allowed us to evaluate the migration capacity of the cells, minimizing the contribution of cell proliferation. Although we did not find any differences in the studied parameter, and a decrease in the scratch width was observed both in the control and in the cells treated with the studied compounds, when evaluating the antiproliferative potential of the diphenylamines after 48 and 72 h, compound **6b** demonstrated a clear ability to slow down the closure of the defect (control in Figure 4). Thus, in the control samples on the second and third days of the experiment, 63.83 ± 2.47% and 48.191 ± 3.16% of the unhealed defect area remained from the original, while **6b** managed to significantly increase this parameter.

**Figure 5:**
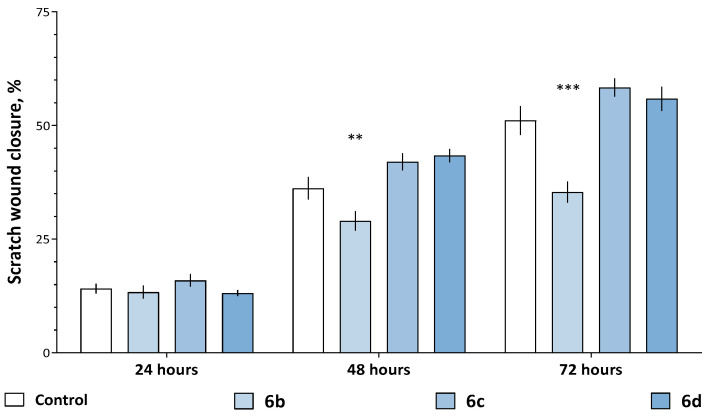
Histogram showing the area of the scratch overgrowth of A549 cells under the action of 2-aminodiphenylamines **6b**, **6c** and **6d** at different time intervals. The concentration of the studied compounds was 1 μM; the control samples contained an equivalent volume of solvent (1% DMSO). **—*p* ≤ 0.01; ***—*p* ≤ 0.001 (two-way ANOVA, the Bonferroni post hoc test).

Additionally, Figure 5 shows a quantitative analysis of the proliferative activity of the A549 cell line under the influence of the studied molecules, where a significant decrease in the proliferation coefficient of cells exposed to 2-aminodiphenylamine **6b** containing two trifluoromethyl substituents is confirmed, compared to the control group. A similar ability of the diphenylamine derivative to suppress the migration of cancer cells, shown in the work of Yan et al. [72], was accompanied by excellent antiproliferative activity and a pronounced inhibitory effect on tumors in mice with HT29 xenografts.

Thus, the obtained results indicate that 2-aminodiphenylamine **6b** has the ability to clearly inhibit the proliferation of tumor cells, which correlates with its most pronounced cytotoxic activity.

## 3. Materials and Methods

### 3.1. Chemistry

#### 3.1.1. Reagents and Materials

The solvents and reagents used for this project were purchased from Acros Organics (Geel, Belgium) and used without purification unless otherwise stated. The melting points of compounds were determined on a PolyTherm A (München, Germany) heating stage at a heating rate of 4 °C/min and were not corrected. ^1^H and ^13^C NMR spectra were recorded on a Bruker (Billerica, Massachusetts, USA) DRX500 instrument (the frequencies for ^1^H and ^13^C were 500 and 125 MHz, respectively) or Bruker DRX400 instrument (the frequencies for ^1^H and ^13^C were 400 and 100 MHz, respectively) in DMSO-*d*_6_ at 303 K (^1^H and ^13^C NMR spectra are in Supplementary Materials). The residual proton (*δ* 2.5) and carbon (*δ* 39.5) signals of the DMSO-*d*_6_ were used as internal standards. The assignment of the proton and carbon spectra was carried out using 1D NOE, 2D {1H-1H} NOESY, {^1^H-^13^C} HSQC and {^1^H-^13^C} HMBC spectroscopy. High resolution mass spectra were recorded on a MicrOTOF II instrument (Bruker Daltonics, Billerica, Massachusetts, USA) using electrospray ionization. The mass scanning range (*m*/*z* 50) was 3000 Da; the compounds were injected with a syringe. MeCN and MeOH solvents were used; the solution flow rate was 4.0 μL/min.

#### 3.1.2. Synthesis and General Procedures

General procedure for the synthesis of 1-(2-nitroaryl)-1*H*-benzimidazoles (**3a,b**) and 1-(2-nitroaryl)-1*H*-benzotriazoles (**3c**–**e**).
K_2_CO_3_ (20.70 g, 0.15 mol) and **2a**–**c** (0.1 mol) were added to a solution of **1a, b** (0.1 mol) in DMF (100 mL). The reaction mixture was stirred at 110 °C for 2 h in the synthesis of **3a**, 3 h in the syntheses of **3c, d** or 7 h in the syntheses of **3b, e**. The reaction mixture was poured into water. The precipitate was filtered off and crystallized from *i*-PrOH.
*1-(2-Nitro-4-(trifluoromethyl)phenyl)-1H-benzimidazole* (**3a**): Yield 98%. m.p. 130−132 °C. ^1^H NMR (500 MHz, DMSO-*d*_6_) *δ* 8.71 (d, 1H, H^3′^, *J* = 1.5 Hz), 8.54 (s, 1H, H^2^), 8.41 (dd, 1H, H^5′^, *J* = 8.3, 1.6 Hz), 8.16 (d, 1H, H^6′^, *J* = 8.3 Hz), 7.78–7.82 (m, 1H, H^4^), 7.29–7.39 (m, 3H, H^5^, H^6^, H^7^). ^13^C{^1^H} NMR (126 MHz, DMSO-*d*_6_) *δ* 145.13 (C^2′^), 143.39 (C^2^), 143.10 (C^4a^), 133.71 (C^7a^), 132.19 (C^1′^), 131.58 (q, *J* = 3.4 Hz, C^5′^), 131.37 (C^6′^), 130.04 (q, *J* = 34.2 Hz, C^4′^), 123.88 (C^6^), 123.48 (q, *J* = 3.6 Hz, C^3′^), 122.90 (C^5^), 122.78 (q, *J* = 273.6 Hz, CF_3_), 120.00 (C^4^), 109.79 (C^7^). HRMS (ESI/TOF) *m*/*z* calculated for C_14_H_9_F_3_N_3_O_2_ [M+H]^+^: 308.0648. Found: 308.0631.
*1-(4-Chloro-2-nitrophenyl)-1H-benzimidazole* (**3b**): Yield 94%. m.p. 102–105 °C. ^1^H NMR (500 MHz, DMSO-*d*_6_) *δ* 8.48 (s, 1H, H^2^), 8.47 (d, 1H, H^3′^, *J* = 2.4 Hz), 8.09 (dd, 1H, H^5′^, *J* = 8.5, 2.4 Hz), 7.93 (d, 1H, H^6′^, *J* = 8.5 Hz), 7.76–7.81 (m, 1H, H^4^), 7.27–7.35 (m, 2H, H^5^, H^6^), 7.23–7.27 (m, 1H, H^7^). ^13^C{^1^H} NMR (126 MHz, DMSO-*d*_6_) *δ* 145.61 (C^2′^), 143.63 (C^2^), 143.01 (C^4a^), 134.79 (C^5′^), 134.30 (C^4′^), 134.05 (C^7a^), 131.76 (C^6′^), 127.59 (C^1′^), 125.88 (C^3′^), 123.78 (C^6^), 122.72 (C^5^), 119.94 (C^4^), 109.77 (C^7^). HRMS (ESI/TOF) *m*/*z* calculated for C_13_H_9_ClN_3_O_2_ [M+H]^+^: 274.0384. Found: 274.0369.
*1-[2-Nitro-4-(trifluoromethyl)phenyl]-1H-benzotriazole* (**3c**): Yield 93%. m.p. 133–136 °C. ^1^H NMR (400 MHz, DMSO-*d*_6_) *δ* 9.07 (d, 1H, H^3′^, *J* = 2.6 Hz), 8.81 (dd, 1H, H^5′^, *J* = 8.8, 2.5Hz), 8.40 (d, 1H, H^6′^, *J* = 8.8 Hz), 8.28 (d, 1H, H^4^, *J* = 8.4 Hz), 7.85 (d, 1H, H^7^, *J* = 8.4 Hz), 7.77 (td, 1H, H^6^, *J* = 8.3, 1.0 Hz), 7.61 (td, 1H, H^5^, *J* = 8.3, 1.0 Hz). ^13^C NMR (101 MHz, DMSO-*d*_6_) *δ* 145.93, 145.32, 133.31, 132.30, 132.00, 131.41 (q, *J* = 35 Hz), 130.32, 129.87, 126.07, 125.99 (q, *J* = 272 Hz), 124.38, 120.65, 111.03. HRMS (ESI/TOF) *m*/*z* calculated for C_13_H_7_F_3_N_4_O_2_: 309.2196 [M + H]^+^. Found: 309.2191.
*Ethyl 4-(1H-benzotriazol-1-yl)-3-nitrobenzoate* (**3d**): Yield 91%. m.p. 115–119 °C. ^1^H NMR (400 MHz, DMSO-*d*_6_) *δ* 8.70 (d, *J* = 1.8 Hz, 1H, H^2^), 8.51 (dd, *J* = 8.2, 1.9 Hz, 1H, H^6^), 8.29–8.19 (m, 2H, H^4′^, H^5^), 7.80 (dt, *J* = 8.4, 1.1 Hz, 1H, H^7′^), 7.72 (td, *J* = 8.3, 1.0 Hz, 1H, H^6′^), 7.58 (td, *J* = 8.3, 1.0 Hz, 1H, H^5′^), 4.43 (q, *J* = 7.1 Hz, 2H, CH_2_), 1.38 (t, *J* = 7.1 Hz, 3H, CH_3_). ^13^C NMR (101 MHz, DMSO-*d*_6_) *δ* 163.97, 145.95, 144.97, 135.52, 133.25, 132.58, 132.16, 130.25, 129.15, 127.21, 126.00, 120.66, 110.92, 62.72, 14.71. HRMS (ESI/TOF) *m*/*z* calculated for C_15_H_13_N_4_O_4_: 313.2876 [M + H]^+^. Found: 313.2873.
*1-(4-Chloro-2-nitrophenyl)-1H-benzotriazole* (**3e**): Yield 89%. m.p. 134–137 °C. ^1^H NMR (400 MHz, DMSO-*d*_6_) *δ* 8.51 (d, *J* = 2.2 Hz, 1H, H^3′^), 8.23 (d, *J* = 8.5 Hz, 1H, H^4^), 8.15 (dd, *J* = 8.5, 1.5 Hz, 1H, H^5′^), 8.11 (d, *J* = 8.5, 1H, H^6′^), 7.76 (d, *J* = 8.2 Hz, 1H, H^7^), 7.69 (td, *J* = 8.3, 1.0 Hz, 1H, H^6^), 7.56 (td, *J* = 8.3, 1.0 Hz, 1H, H^5^). ^13^C NMR (101 MHz, DMSO-*d*_6_) *δ* 145.79, 135.97, 135.38, 133.63, 130.49, 130.04, 127.75, 126.75, 125.81, 120.50, 110.88. HRMS (ESI/TOF) *m*/*z* calculated for C_12_H_8_ClN_4_O_2_: 275.6699 [M + H]^+^. Found: 275.6698.
General procedure for the synthesis of 1-(2-aminoaryl)-1*H*-benzimidazoles (**4a,b**) and 1-(2-aminoaryl)-1*H*-benzotriazoles (**4c**–**e**).

15% solution of TiCl_3_ (144 mL, 0.14 mol) in 10% HCl at 70 °C was added to the solution of compounds **3a**–**e** (0.02 mol) in isopropanol (100 mL). After 5 min, the reaction mixture was treated with 25% aqueous ammonia to pH = 7-8 and extracted with a few portions of hot chloroform (Σ = 250 mL). The chloroform was distilled off to give compounds **4a**–**e**.

*2-(1H-benzimidazol-1-yl)-5-(trifluoromethyl)aniline* (**4a**)**:** Yield 97%. m.p. 209−210 °C. ^1^H NMR (500 MHz, DMSO-*d*_6_) *δ* 8.33 (s, 1H, H^2′^), 7.75–7.81 (m, 1H, H^4′^), 7.35 (d, *J* = 8.1 Hz, 1H, H^3^), 7.23–7.31 (m, 3H, H^5′^, H^6′^, H^6^), 7.17–7.22 (m, 1H, H^7′^), 6.98 (dd, *J* = 8.1, 1.6 Hz, 1H, H^4^), 5.58 (s, 2H, NH_2_). ^13^C{^1^H} NMR (DMSO-*d*_6_, 126 MHz) *δ* 145.27 (C^1^), 143.83 (C^2′^), 143.37 (C^4′a^), 133.69 (C^7′a^), 130.12 (q, *J* = 31.8 Hz, C^5^), 129.13 (C^3^), 124.22 (q, *J* = 272.9 Hz, CF_3_), 123.07 (C^6′^), 122.95 (C^2^), 122.08 (C^5′^), 119.74 (C^4′^), 112.29 (q, *J* = 4.0 Hz, C^6^), 111.99 (q, *J* = 4.0 Hz, C^4^), 110.81 (C^7′^). HRMS (ESI/TOF) *m*/*z* calculated for C_14_H_11_F_3_N_3_ [M + H]^+^: 278.0906. Found: 278.0890.
*2-(1H-benzimidazol-1-yl)-5-chloroaniline* (**4b**)**:** Yield 97%. m.p. 192−195 °C. ^1^H NMR (500 MHz, DMSO-*d*_6_) *δ* 8.26 (s, 1H, H^2′^), 7.72–7.79 (m, 1H, H^4′^), 7.21–7.33 (m, 2H, H^5′^, H^6′^), 7.15–7.19 (m, 1H, H^7′^), 7.13 (d, *J* = 8.3 Hz, 1H, H^3^), 6.98 (d, *J* = 2.3 Hz, 1H, H^6^), 6.69 (dd, *J* = 8.4, 2.4 Hz, 1H, H^4^), 5.39 (s, 2H, NH_2_). ^13^C{^1^H} NMR (126 MHz, DMSO-*d*_6_) *δ* 146.08 (C^1^), 144.05 (C^2′^), 143.32 (C^4′a^), 133.98 (C^7′a^), 133.93 (C^5^), 129.69 (C^3^), 122.92 (C^6′^), 121.92 (C^5′^), 119.66 (C^4′^), 118.81 (C^2^), 115.55 (C^4^), 114.94 (C^6^), 110.69 (C^7′^). HRMS (ESI/TOF) *m*/*z* calculated for C_13_H_11_ClN_3_ [M+H]^+^: 244.0642. Found: 244.0626.
*2-(1H-benzotriazol-1-yl)-5-(trifluoromethyl)aniline***(4c):** Yield 98%. m.p. 136–139 °C. ^1^H NMR (500 MHz, DMSO-*d*_6_) *δ* 8.18 (d, *J* = 8.3 Hz, 1H, H^4′^), 7.59 (ddd, *J* = 8.1, 6.8, 1.0 Hz, 1H, H^6′^), 7.47–7.53 (m, 2H, H^5′^, H^7′^), 7.46 (d, *J* = 8.2 Hz, 1H, H^3^), 7.34 (d, *J* = 2.0 Hz, 1H, H^6^), 7.01 (dd, *J* = 8.2, 2.0 Hz, 1H, H^4^), 5.78 (s, 2H, NH_2_). ^13^C NMR (DMSO-*d*_6_, 126 MHz) *δ* 145.91, 145.50, 133.74, 131.76 (q, *J* = 32 Hz), 129.29, 128.96, 125.02, 124.71 (q, *J* = 271 Hz, CF_3_), 123.51 (m), 120.42, 113.44 (q, *J* = 4.1 Hz), 112.55 (q, *J* = 4 Hz), 111.46. HRMS (ESI/TOF) *m*/*z* calculated for C_13_H_10_F_3_N_4_: 279.2400 [M + H]^+^. Found: 279.2398.
*Ethyl 3-amino-4-(1H-benzotriazol-1-yl)benzoate* (**4d**): Yield 95%. m.p. 132–136 °C. ^1^H NMR (400 MHz, DMSO-*d*_6_) *δ* 8.17 (d, *J* = 8.2 Hz, 1H, H^4′^), 7.65 (d, *J* = 1.9 Hz, 1H, H^2^), 7.59 (t, *J* = 8.4 Hz, 1H, H^6′^), 7.52–7.44 (m, 2H, H^5′^, H^7′^), 7.37 (d, *J* = 8.2 Hz, 1H, H^5^), 7.29 (dd, *J* = 8.2, 1.9 Hz, 1H, H^6^), 5.60 (s, 2H, NH_2_), 4.35 (q, *J* = 7.1 Hz, 2H, CH_2_), 1.34 (t, *J* = 7.1 Hz, 3H, CH_3_). ^13^C NMR (DMSO-*d*_6_, 101 MHz) *δ* 166.15, 145.90, 144.83, 133.66, 132.40, 128.90, 128.25, 125.00, 124.30, 120.22, 117.95, 117.11, 111.53, 61.55, 14.84. HRMS (ESI/TOF) *m*/*z* calculated for C_15_H_15_N_4_O: 267.3053 [M + H]^+^. Found: 267.3051.
*2-(1H-benzotriazol-1-yl)-5-chloroaniline* (**4e***).* Yield 94%. m.p. 154–156 °C. ^1^H NMR (400 MHz, DMSO-d6) *δ* 8.16 (d, *J* = 8.3 Hz, 1H, H^4′^), 7.58 (dd, *J* = 8.3, 6.8 Hz, 1H, H^6′^), 7.49–7.45 (m, 2H, H^5′^, H^7′^), 7.24 (d, *J* = 8.4 Hz, 1H, H^3^), 7.03 (d, *J* = 2.3 Hz, 1H, H^6^), 6.74 (dd, *J* = 8.4, 2.3 Hz, 1H, H^4^), 5.55 (s, 2H). ^13^C NMR (101 MHz, DMSO-*d*_6_,) *δ* 146.38, 145.85, 135.64, 133.97, 129.85, 128.79, 124.88, 120.16, 119.70, 116.20, 116.00, 111.38. HRMS (ESI/TOF) *m*/*z* calculated for C_12_H_10_ClN_4_: 245.6871 [M + H]^+^. Found: 245.6873.
General procedure for the synthesis of *N*-(2-(1*H*-benzimidazol-1-yl)-5-(trifluoromethyl)phenyl)hydroxylamine (**8a**) and *N*-(2-(1*H*-benzotriazol-1-yl)-5-(trifluoromethyl)phenyl)hydroxylamine (**8b**)

A solution of SnCl_2_∙2H_2_O (2.37 g, 0.015 mol) in 18% HCl (25 mL) was added at 70 °C to a solution of compound **3a** or **3c** (0.005 mol) in isopropanol (25 mL). After 5 min, the reaction mixture was treated with 25% aqueous ammonia to pH = 7–8 and extracted with a few portions of chloroform (Σ = 150 mL). The solvent was distilled off and the residue was recrystallized from *i*-PrOH to give compound **8a** or **8b**.

*N-(2-(1H-benzimidazol-1-yl)-5-(trifluoromethyl)phenyl)hydroxylamine* (**8a**)*:* Yield 86%. m.p. 153–156 °C. ^1^H NMR (DMSO-*d*6, 500 MHz) *δ* 8.79 (d, *J* = 1.6 Hz, 1H, OH), 8.61 (s, 1H, NH), 8.31 (s, 1H, H^2′^), 7.79–7.23 (m, 1H, H^4′^), 7.59 (d, *J* = 1.2 Hz, 1H, H^6^), 7.48 (d, *J* = 7.9 Hz, 1H, H^3^), 7.32–7.24 (m, 3H, H^5′^, H^4^, H^6′^), 7.24–7.18 (m, 1H, C^7′^). ^13^C{^1^H} NMR (DMSO-*d*6, 126 MHz) *δ* 148.0 (C^1^), 143.7 (C^2′^), 143.4 (C^4′a^), 133.7 (C^7′a^), 129.9 (q, *J* = 31.9 Hz, C^5^), 128.3 (C^3^), 124.1 (q, *J* = 272.5 Hz, CF_3_), 123.5 (C^2^), 122.9 (C^6′^), 122.1 (C^5′^), 119.7 (C^4′^), 115.6 (q, *J* = 3.8 Hz, C^4^), 111.1 (C^7′^), 110.2 (q, *J* = 3.8 Hz, C^6^). HRMS (ESI/TOF) *m*/*z* calculated for C_14_H_11_F_3_N_3_O [M+H]^+^: 294.0855. Found: 294.0841.
*N-(2-(1H-benzotriazol-1-yl)-5-(trifluoromethyl)phenyl)hydroxylamine* (**8b**): Yield 82%. m.p. 151–154 °C. ^1^H NMR (400 MHz, DMSO-*d*_6_) *δ* 8.87 (s, 1H, OH), 8.68 (s, 1H, NH), 8.18 (dt, *J* = 8.3, 1.0 Hz, 1H, H^4′^), 7.64–7.55 (m, 3H, H^6^, H^3^, H^5′^,), 7.55–7.43 (m, 2H, H^6′^, H^7′^), 7.30 (dd, *J* = 8.2, 2.1 Hz, 1H, H^4^). ^13^C NMR (101 MHz, DMSO-*d*_6_) *δ* 148.11, 145.96, 133.77, 131.60 (q, *J* = 31.8 Hz), 128.79, 126.07, 124.98, 123.86, 123.36, 120.22, 115.95, 111.77, 111.05. HRMS (ESI/TOF) *m*/*z* calculated for C_13_H_10_F_3_N_4_O [M+H]^+^: 295.2394. Found: 295.2389.
General procedure for the synthesis of *N*-[2-(1*H*-benzimidazol-1-yl)-5-R-phenyl]-2-nitro-4-R1-aniline (**5a**) and *N*-[2-(1*H*-benzotriazol-1-yl)-5-R-phenyl]-2-nitro-4-R1-aniline (**5b**–**d**).

K_2_CO_3_ (3.17 g, 0.023 mol) and **2a,b** (0.015 mol) were added to a solution of **4a,c,e** (0.015 mol) in DMF (100 mL). The reaction mixture was stirred for 12 h at 110 °C. The reaction mixture was poured into water. The precipitate was filtered off and crystallized from *i*-PrOH.

*N-[2-(1H-benzimidazol-1-yl)-5-(trifluoromethyl)phenyl]-2-nitro-4-(trifluoromethyl)aniline* (**5a**). Yield 93%. m.p. 180–183 °C. ^1^H NMR (400 MHz, DMSO-*d*_6_) δ 9.79 (s, 1H, NH), 8.36 (s, 1H, H^2′’^), 8.11 (s, 1H, H^6′^), 8.03 (s, 1H, H^3^), 7.99–7.92 (m, 2H, H^3′^, H^4′^), 7.60–7.53 (m, 1H, H^4′’^), 7.45–7.38 (m, 2H, H^5^, H^7′’^), 7.22–7.14 (m, 2H, H^5′’^, H^6′’^), 6.82 (d, *J* = 8.9 Hz, 1H, H^6^). ^13^C NMR (101 MHz, DMSO-*d*_6_) δ 143.40, 142.96, 142.73, 135.01, 134.80, 132.43, 132.39, 131.02 (q, *J* = 3.0 Hz), 129.86 (q, *J* = 32.6 Hz), 129.25, 126.17 (q, *J* = 3.6 Hz), 124.58 (q, *J* = 4.5 Hz), 123.57 (q, *J* = 272.5 Hz), 123.35, 123.30 (q, *J* = 271.1 Hz), 123.12 (q, *J* = 4.4 Hz), 122.50, 119.58, 118.10 (q, *J* = 33.8 Hz), 118.00, 110.89. HRMS (ESI/TOF) *m*/*z* calculated for C_21_H_13_F_6_N_4_O_2_: 467.3434 [M + H]^+^. Found: 467.3435.
*N-[2-(1H-benzotriazol-1-yl)-5-(trifluoromethyl)phenyl]-2-nitro-4-(trifluoromethyl)aniline* (**5b**). Yield 92%. m.p. 203–204 °C. ^1^H NMR (400 MHz, DMSO-*d*_6_) *δ* 9.93 (s, 1H, NH), 8.16 (s, 1H, H^6′^), 8.08 (d, *J* = 8.3 Hz, 1H, H^3′^), 8.04 (s, 1H, H^3^), 8.02 (d, *J* = 9.0 Hz, 1H, H^4′’^), 7.97 (d, *J* = 8.5 Hz, 1H, H^4′^), 7.73 (d, *J* = 8.3 Hz, 1H, H^7′’^), 7.58–7.47 (m, 2H, H^5^, H^6′’^), 7.41 (t, *J* = 7.8 Hz, 1H, H^5′’^), 6.88 (d, *J* = 8.9 Hz, 1H, H^6^). ^13^C NMR (101 MHz, DMSO-*d*_6_) *δ* 144.94, 142.46, 134.41, 134.08, 133.06, 131.94, 131.11 (q, *J* = 2.9 Hz), 130.84 (q, *J* = 32.4 Hz), 128.71, 128.60, 125.45 (m), 124.69, 124.06 (m), 123.42 (q, *J* = 272.7 Hz), 123.21 (q, *J* = 271.1 Hz), 123.12 (q, *J* = 4.5 Hz), 119.40, 118.59 (q, *J* = 33.8 Hz), 118.09, 110.87. HRMS (ESI/TOF) *m*/*z* calculated for C_20_H_12_F_6_N_5_O_2_: 468.3315 [M + H]^+^. Found: 468.3312.
*Ethyl 4-[2-(1H-benzotriazol-1-yl)-5-(trifluoromethyl)anilino]-3-nitrobenzoate* (**5c**). Yield 91%. m.p. 192–196 °C. ^1^H NMR (400 MHz, DMSO-*d*_6_) *δ* 10.00 (s, 1H, NH), 8.28 (s, 1H, H^2^), 8.15 (s, 1H, H^6′^), 8.08 (d, *J* = 8.3 Hz, 1H, H^3′^), 8.01 (d, *J* = 8.3 Hz, 1H, H^4′’^), 7.97 (d, *J* = 8.5 Hz, 1H, H^4′^), 7.74 (d, *J* = 8.2 Hz, 1H, H^7′’^), 7.68 (d, *J* = 9.0 Hz, 1H, H^6^), 7.53 (t, *J* = 7.6 Hz, 1H, H^6′’^), 7.40 (t, *J* = 7.6 Hz, 1H, H^5′’^), 6.79 (d, *J* = 8.9 Hz, 1H, H^5^), 4.24 (q, *J* = 7.1 Hz, 2H, CH_2_), 1.26 (t, *J* = 7.1 Hz, 3H, CH_3_). ^13^C NMR (101 MHz, DMSO-*d*_6_) *δ* 163.50, 144.77, 142.74, 134.60, 134.23, 133.89, 133.17, 131.84, 130.66 (q, *J* = 32.8 Hz), 128.47, 128.34, 126.81, 124.95 (q, *J* = 3.7 Hz), 124.41, 123.76 (q, *J* = 3.8 Hz), 123.21 (q, *J* = 273.1 Hz), 119.82, 119.21, 116.75, 110.60, 60.62, 13.76. HRMS (ESI/TOF) *m*/*z* calculated for C_22_H_17_F_3_N_5_O_4_: 472.3961 [M + H]^+^. Found: 472.3958.
*N-[2-(1H-benzotriazol-1-yl)-5-chlorophenyl]-2-nitro-4-(trifluoromethyl)aniline* (**5d**). Yield 93%. m.p. 178–181 °C. ^1^H NMR (400 MHz, DMSO-*d*_6_) *δ* 9.79 (s, 1H, NH), 8.05 (s, 1H, H^3^), 8.01 (d, *J* = 8.3 Hz, 1H, H^4′’^), 7.88 (s, 1H, H^6′^), 7.87 (d, *J* = 6.0 Hz, 1H, H^3′^), 7.71–7.65 (m, 2H, H^4′^, H^7′’^), 7.58–7.49 (m, 2H, H^5^, H^6′’^), 7.40 (t, *J* = 7.6 Hz, 1H, H^5′’^), 6.95 (d, *J* = 8.9 Hz, 1H, H^6^). ^13^C NMR (101 MHz, DMSO-*d*_6_) *δ* 144.86, 142.60, 135.06, 134.79, 132.85, 132.21, 131.21 (q, *J* = 3.5 Hz), 129.93, 129.15, 128.45, 127.87, 127.41, 124.56, 123.22 (q, *J* = 271.2 Hz), 123.17 (q, *J* = 4.3 Hz), 119.35, 118.51 (q, *J* = 33.8 Hz), 118.28, 110.79. HRMS (ESI/TOF) *m*/*z* calculated for C_19_H_12_ClF_3_N_5_O_2_: 434.7785 [M + H]^+^. Found: 434.7781.

General procedure for the synthesis of *N*-[2-(1*H*-benzimidazol-1-yl)-5-R-phenyl]-2-amino-4-R1-aniline (**6a**) and *N*-[2-(1*H*-benzotriazol-1-yl)-5-R-phenyl]-2-amino-4-R1-aniline (**6b**–**d**).

The reduction of nitro compounds **5** was carried out similarly to the reduction of nitroarenes **3**.

*N-[2-(1H-benzimidazol-1-yl)-5-(trifluoromethyl)phenyl]-2-amino-4-(trifluoromethyl)aniline* (**6a**). Yield 96%. m.p. 205–207 °C. ^1^H NMR (400 MHz, DMSO-*d*_6_) *δ* 8.51 (s, 1H, H^2′’^), 7.77–7.70 (m, 1H, H^4′’^), 7.56 (d, *J* = 8.1 Hz, 1H, H^3′^), 7.48–7.40 (m, 2H, NH, H^7′’^), 7.33–7.23 (m, 3H, H^4′^, H^5′’^, H^6′’^), 7.03 (d, *J* = 8.1 Hz, 1H, H^6^), 7.01 (d, *J* = 1.6 Hz, 1H, H^3^), 6.95 (d, *J* = 1.6 Hz, 1H, H^6′^), 6.72 (dd, *J* = 8.1, 1.6 Hz, 1H, H^5^), 5.31 (s, 2H, NH_2_). ^13^C NMR (101 MHz, DMSO-*d*_6_) *δ* 144.10, 143.44, 143.02, 141.14, 133.62, 129.65 (q, *J* = 31.7 Hz), 129.07, 128.97, 126.93, 125.52 (q, *J* = 31.3 Hz), 124.52 (q, *J* = 271.8 Hz), 124.04, 123.92 (q, *J* = 272.4 Hz), 123.13, 122.13, 119.61, 115.75 (q, *J* = 3.9 Hz), 113.10 (m), 112.50 (q, *J* = 4.2 Hz), 111.08 (q, *J* = 3.3 Hz), 110.97. HRMS (ESI/TOF) *m*/*z* calculated for C_21_H_15_F_6_N_4_: 437.3605 [M + H]^+^. Found: 437.3601.
*N-[2-(1H-benzotriazol-1-yl)-5-(trifluoromethyl)phenyl]-2-amino-4-(trifluoromethyl)aniline* (**6b**). Yield 94%. m.p. 170–173 °C. ^1^H NMR (400 MHz, DMSO-*d*_6_) *δ* 8.14 (d, *J* = 8.4 Hz, 1H, H^4′’^), 7.77 (d, *J* = 8.3 Hz, 1H, H^7′’^), 7.68 (d, *J* = 8.1 Hz, 1H, H^3′^), 7.56 (t, *J* = 7.6 Hz, 1H, H^6′’^), 7.52 (s, 1H, NH), 7.45 (t, *J* = 7.6 Hz, 1H, H^5′’^), 7.33 (d, *J* = 8.2 Hz, 1H, H^4′^), 7.07–7.02 (m, 2H, H^6′^, H^6^), 6.98 (s, 1H, H^3^), 6.73 (d, *J* = 8.1 Hz, 1H, H^5^), 5.28 (s, 2H, NH_2_). ^13^C NMR (101 MHz, DMSO-*d*_6_) *δ* 145.37, 142.92, 140.66, 132.96, 130.82 (q, *J* = 31.7 Hz), 129.44, 128.53, 128.18, 126.6, 125.63 (q, *J* = 31.3 Hz), 124.50 (q, *J* = 271.7 Hz), 124.29, 123.95, 123.81 (q, *J* = 272.7 Hz), 119.45, 115.53 (q, *J* = 3.9 Hz), 113.35 (q, *J* = 3.9 Hz), 112.50 (q, *J* = 4.3 Hz), 111.39, 111.16 (q, *J* = 4.1 Hz). HRMS (ESI/TOF) *m*/*z* calculated for C_20_H_14_F_6_N_5_: 438.3485 [M + H]^+^. Found: 438.3481.
*Ethyl 3-amino-4-[2-(1H-benzotriazol-1-yl)-5-(trifluoromethyl)anilino]benzoate* (**6c**). Yield 92%. m.p. 178–181 °C. ^1^H NMR (400 MHz, DMSO-*d*_6_) *δ* 8.14 (d, *J* = 8.3 Hz, 1H, H^4′’^), 7.75 (d, *J* = 8.3 Hz, 1H, H^7′’^), 7.69 (d, *J* = 8.2 Hz, 1H, H^3′^), 7.56 (t, *J* = 7.7 Hz, 1H, H^6′’^), 7.53 (s, 1H, NH), 7.45 (t, *J* = 7.6 Hz, 1H, H^5′’^), 7.34 (d, *J* = 8.0 Hz, 1H, H^4′^), 7.32 (d, *J* = 1.7 Hz, 1H, H^3^), 7.11 (s, 1H, H^6′^), 7.06 (dd, *J* = 8.2, 1.7 Hz, 1H, H^5^), 6.97 (d, *J* = 8.2 Hz, 1H, H^6^), 5.09 (s, 2H, NH_2_), 4.23 (q, *J* = 7.1 Hz, 2H, CH_2_), 1.28 (t, *J* = 7.1 Hz, 3H, CH_3_). ^13^C NMR (101 MHz, DMSO-*d*_6_) *δ* 165.76, 145.34, 141.86, 140.38, 132.88, 130.73 (q, *J* = 31.7 Hz), 129.87, 129.34, 128.17, 126.94, 126.14, 124.27, 123.81 (q, *J* = 272.8 Hz), 122.51, 122.45, 119.44, 117.59, 115.80 (m), 113.92 (m), 111.32, 60.22, 14.22. HRMS (ESI/TOF) *m*/*z* calculated for C_22_H_19_F_3_N_5_O_2_: 442.4132 [M + H]^+^. Found: 442.4134.
*N-[2-(1H-benzotriazol-1-yl)-5-chlorophenyl]-2-amino-4-(trifluoromethyl)aniline* (**6d**). Yield 91%. m.p. 191–194 °C. ^1^H NMR (400 MHz, DMSO-*d*_6_) *δ* 8.13 (d, *J* = 8.3 Hz, 1H, H^4′’^), 7.72 (d, *J* = 8.3 Hz, 1H, H^7′’^), 7.55 (t, *J* = 7.6 Hz, 1H, H^6′’^), 7.49–7.41 (m, 2H, H^3′^, H^5′’^), 7.32 (s, 1H, NH), 7.09–7.02 (m, 2H, H^6^, H^4′^), 6.98 (s, 1H, H^3^), 6.78 (d, *J* = 1.8 Hz, 1H, H^6′^), 6.75 (d, *J* = 8.2 Hz, 1H, H^5^), 5.25 (s, 2H, NH_2_). ^13^C NMR (101 MHz, DMSO-*d*_6_) *δ* 145.30, 142.89, 141.52, 135.05, 133.20, 129.86, 128.77, 128.01, 125.54 (q, *J* = 31.2 Hz), 124.51 (q, *J* = 271.7 Hz), 124.13, 122.57, 119.37, 118.97, 116.23, 112.58, 112.53, 111.19, 11.14. HRMS (ESI/TOF) *m*/*z* calculated for C_19_H_14_ClF_3_N_5_: 404.7956 [M + H]^+^. Found: 404.7953.
General procedure for the synthesis of 1-[2-(1*H*-benzimidazol-1-yl)phenyl]-1*H*-benzotriazole (**7a**–**c**):

Formic acid (0.6 mL, 0.016 mol) was added to a solution of **6b**–**d** (0.01 mol) in DMF (20 mL). The reaction mixture was heated for 1 h at 90 °C. After cooling, the reaction mixture was poured into water and treated with a 25% aqueous ammonia solution to pH = 7. The precipitate was filtered off and crystallized from *i*-PrOH.

*1-{4-(trifluoromethyl)-2-[5-(trifluoromethyl)-1H-benzimidazol-1-yl]phenyl}-1H-benzotriazole* (**7a**). Yield 96%. m.p. 124–128 °C. ^1^H NMR (400 MHz, DMSO-*d*_6_) *δ* 8.61 (s, 1H, H^2′’^), 8.57 (s, 1H, H^3′^), 8.35–8.27 (m, 2H, H^5′^, H^6′^), 7.97 (d, *J* = 8.3 Hz, 1H, H^4^), 7.93 (s, 1H, H^4′’^), 7.59 (d, *J* = 8.3 Hz, 1H, H^7^), 7.47 (t, *J* = 7.7 Hz, 1H, H^6^), 7.38–7.31 (m, 2H, H^5^, H^6′’^), 7.18 (d, *J* = 8.5 Hz, 1H, H^7′’^). ^13^C NMR (101 MHz, DMSO-*d*_6_) *δ* 146.17, 144.63, 142.24, 135.61, 135.09, 132.35, 131.41 (q, *J* = 33.6 Hz), 131.16, 129.32, 128.94, 127.92 (q, *J* = 4.0 Hz), 127.11 (q, *J* = 3.8 Hz), 124.89, 124.61 (q, *J* = 272.0 Hz), 123.41 (q, *J* = 31.8 Hz), 123.22 (q, *J* = 273.1 Hz), 119.99 (q, *J* = 3.7 Hz), 119.48, 117.11 (q, *J* = 4.0 Hz), 110.75, 109.98. HRMS (ESI/TOF) *m*/*z* calculated for C_21_H_12_F_6_N_5_: 448.3434 [M + H]^+^. Found: 448.3429.
*Ethyl 1-[2-(1H-benzotriazol-1-yl)-5-(trifluoromethyl)phenyl]-1H-benzimidazole-5-carboxylate* (**7b**). Yield 94%. m.p. 100–104 °C. ^1^H NMR (400 MHz, DMSO-*d*_6_) *δ* 8.61 (s, 1H, H^2^), 8.57 (s, 1H, H^6′^), 8.36–8.24 (m, 2H, H^3′^, H^4′^), 8.10 (s, 1H, H^4^), 7.96 (d, *J* = 8.2 Hz, 1H, H^4′’^), 7.60–7.51 (m, 2H, H^6^, H^7′’^), 7.46 (t, *J* = 7.4 Hz, 1H, H^6′’^), 7.34 (t, *J* = 7.5 Hz, 1H, H^5′’^), 7.01 (d, *J* = 8.8 Hz, 1H, H^7^), 4.25 (q, *J* = 7.1 Hz, 2H, CH_2_), 1.28 (t, *J* = 6.2 Hz, 3H, CH_3_). ^13^C NMR (101 MHz, DMSO-*d*_6_) *δ* 165.68, 145.89, 144.61, 142.40, 136.32, 134.97, 132.27, 131.31 (q, *J* = 33.0 Hz), 131.20, 129.34, 128.91, 127.79 (q, *J* = 3.5 Hz), 127.02 (q, *J* = 3.7 Hz), 124.84, 124.34, 124.14, 123.21 (q, *J* = 273.0 Hz), 121.12, 119.49, 109.92, 109.66, 60.66, 14.14. HRMS (ESI/TOF) *m*/*z* calculated for C_23_H_17_F_3_N_5_O_2_: 452.4081 [M + H]^+^. Found: 452.4079.
*1-{4-Chloro-2-[5-(trifluoromethyl)-1H-benzimidazol-1-yl]phenyl}-1H-benzotriazole* (**7c**). Yield 97%. m.p. 136–141 °C. ^1^H NMR (400 MHz, DMSO-*d*_6_) *δ* 8.52 (s, 1H, H^2′’^), 8.28 (d, *J* = 2.1 Hz, 1H, H^3′^), 8.09 (d, *J* = 8.6 Hz, 1H, H^6′^), 8.02 (dd, *J* = 8.6, 2.1 Hz, 1H, H^5′^), 7.95 (d, *J* = 8.3 Hz, 1H, H^4^), 7.91 (s, 1H, H^4′’^), 7.52 (d, *J* = 8.3 Hz, 1H, H^7^), 7.44 (t, *J* = 7.6 Hz, 1H, H^6^), 7.37–7.30 (m, 2H, H^5^, H^6′’^), 7.22 (d, *J* = 8.5 Hz, 1H, H^7′’^). ^13^C NMR (101 MHz, DMSO-*d*_6_) *δ* 146.01, 144.53, 142.14, 135.58, 135.49, 132.57, 131.76, 130.94, 130.75, 129.81, 129.47, 128.74, 124.70, 124.62 (q, *J* = 271.8 Hz), 123.41 (q, *J* = 31.7 Hz), 120.03 (q, *J* = 3.7 Hz), 119.39, 117.07 (q, *J* = 4.1 Hz), 110.85, 109.83. HRMS (ESI/TOF) *m*/*z* calculated for C_20_H_12_ClF_3_N_5_: 414.7904 [M + H]^+^. Found: 414.7901.

### 3.2. Biological Evaluation

#### 3.2.1. Cell Culture

For the experiments, we used tumor cell cultures SH-SY5Y, human neuroblast-like cells, A549, human lung adenocarcinoma, MCF-7, human mammary adenocarcinoma, SW-480 and human colorectal adenocarcinoma, as well as cells of normal origin obtained from human embryonic kidneys, Hek-293. The cell lines were obtained from the collection of the Institute of Cytology, Russian Academy of Sciences (St. Petersburg, Russia).

All cells were cultured in the Dulbecco Modified Eagle’s Medium (DMEM) with a high glucose content, 10% fetal bovine serum and 1% penicillin-streptomycin as an antibiotic, in a moist incubator with 5% CO_2_ at 37°C.

#### 3.2.2. MTT-Assay

To measure cellular metabolic activity, a colorimetric analysis was performed using the MTT reagent (3-(4,5–dimethylthiazol-2-yl)-2,5-diphenyltetrazolium bromide), which is an inhibitor of viable cells [73,74].

Briefly, cells in the logarithmic growth phase were digested and resuspended to adjust the cell density. The cells were then seeded in 96-well plates at a density of 10,000 cells per well. After 24 h of the incubation of the cells under the standard conditions described above (for the purpose of cell adhesion), the cells were treated with the synthesized compounds at different concentrations (0.1 to 100 µM) or a vehicle alone as a control (1% DMSO) for 24 h. After the incubation time of the cells with synthesized compounds, 5 mg/mL of MTT was added to the cells and incubated for 2 h at 37 °C. Next, the supernatant was discarded; after that, the formazan crystals were dissolved in a DMSO and the absorbance of each well was measured at 570 nm using a multifunctional microplate reader, Cytation3 (Biotech Tools Inc., Winooski, USA).

#### 3.2.3. Animals and Rat Brain Homogenate

All manipulations with animals were approved by the Bioethics Committee of the Federal State Budgetary Institution of Science Federal Research Center for Problems of Chemical Physics and Medical Chemistry of the Russian Academy of Sciences (Approval No. 70, date 31 March 2023).

In experiments involving the use of animals, male nonlinear rats weighing 200–220 g were used. The animals were kept in a standard vivarium.

To obtain rat brain homogenate, an animal previously anesthetized in a CO_2_ chamber was decapitated using a guillotine. The brain was homogenized in a buffer containing KCl (120 mM) and HEPES (20 mM), pH = 7.4 at 4 ° C and centrifuged at 1500 g to obtain a supernatant. 

The prepared homogenate was used immediately.

#### 3.2.4. TBARS Method

The intensity of the lipid peroxidation of the rat brain homogenate was evaluated using a modified TBARS test. The studied compounds at different concentrations (from 1 to 100 µM) and 0.5 mM Fe(II) (as the initiator of the lipid peroxidation reaction) were added to the rat brain homogenate (2 mg/mL), after which the samples were thermostated for 30 min at 37 °C. In the wells of the negative control, the Fe(II) was replaced with water; in samples with a positive control, a solvent was introduced instead of the studied compounds. 

At the end of incubation, a TBA reagent (0.25 M thiobartituric and 1% trichloroacetic acids dissolved in 0.25 N HCl) was added to each well and heated at 90 °C for 90 min. After that, the samples were centrifuged for 15 min at 6000 g, the supernatant was discarded and the optical density was measured as 540 nm using a multifunctional microplate reader, Cytation3 (Biotech Tools Inc., Winooski, VT, USA). 

#### 3.2.5. Wound-Healing Assay

The migration activity of A549 cells was determined by the wound-healing assay (scratch test) [75,76,77,78,79]. To do this, cells (15,000 cells per well) were seeded in a 96-well plate and cultured under the standard conditions described above. After 24 h of incubation, when the cells became 90% confluent, the medium was selected and an identical scratch wound across the center of each well was made by a 200 µL pipette tip. 

To remove detached cells, the cell monolayer was washed twice with PBS; after that, the growth media and test compounds at 1 µM (or 1% DMSO as a solvent) were added to each well. A phase contrast microscope at a ×40 magnification was used to take photomicrographs after making the wound and then 24 h, 48 h and 72 h later.

The analysis of the obtained images was calculated using the open-source program ImageJ [80] and a special module: Wound_healing_size_tool [81].

## 4. Conclusions

During a series of chemical transformations, the molecular design of hybrid molecules with cytotoxicity against certain cancer cell lines was carried out. The patterns of S*_N_*Ar and reduction reactions used for the formation of bipharmacophore substances have been studied. The factors influencing the regioselectivity of these processes have been established. As a result, effective conditions for the synthesis of hybrid molecules of two types were selected. 

In this study of the biological activity of a number of bipharmacophoric compounds based on condensed polyazaheterocycles and substituted diphenylamines, we found that the most promising hybrid compounds were diphenylamines **6**, containing an amino group and a benzotriazole ring in the *ortho* position to the bridging NH group, which exhibited significant cytotoxic activity. Moreover, varying the substituents in these molecules, namely, replacing the trifluoromethyl group in the *ortho*-phenylenediamine fragment with COOEt, allowed compound **6c** to achieve a selectivity of toxic action specifically with respect to tumor cells without affecting healthy cells, which may be due to the excellent antioxidant properties of this molecule and is categorically considered a positive property of the therapeutic agent. Although **6c** failed to demonstrate the ability to suppress the migratory activity of A549 tumor cells, unlike its analog, **6b**, which contained two CF_3_ groups in its structure, the data obtained suggest the potential for chemical modification, which will result in molecules combining the beneficial properties of hybrids **6b** and **6c**. 

Thus, the competent combination of two complementary fragments in the structure of the hybrid molecules made it possible to obtain compounds that can serve as a promising platform for further optimization in order to obtain effective antitumor compounds.

## Figures and Tables

**Table 1:** IC_50_ values of the cytotoxic effect of the studied compounds **3**–**7**.

Compound	IC_50_, μM *				
	SH-SY5Y	A549	MCF-7	SW-480	Hek-293
**3a**	62.20 ± 0.11	≥100	92.42 ± 1.40	≥100	77.29 ± 2.12
**3b**	≥100	≥100	≥100	≥100	≥100
**3c**	≥100	≥100	≥100	≥100	≥100
**3d**	≥100	≥100	≥100	≥100	≥100
**3e**	≥100	≥100	≥100	≥100	≥100
**4a**	66.75 ± 0.41	67.17 ± 0.12	65.26 ± 0.07	84.03 ± 0.38	82.89 ± 0.46
**4b**	≥100	≥100	≥100	≥100	≥100
**4c**	39.59 ± 0.05	65.38 ± 0.17	75.20 ± 1.43	≥100	≥100
**4d**	≥100	≥100	≥100	≥100	≥100
**4e**	99.13 ± 1.47	91.87 ± 0.02	82.48 ± 0.32	≥100	87.40 ± 1.42
**5a**	≥100	≥100	≥100	≥100	≥100
**5b**	≥100	≥100	≥100	≥100	≥100
**5c**	≥100	≥100	≥100	≥100	≥100
**5d**	≥100	≥100	≥100	≥100	≥100
**6a**	≥100	≥100	≥100	≥100	≥100
**6b**	25.98 ± 0.17	26.28 ± 0.02	23.29 ± 0.12	25.09 ± 0.24	22.43 ± 0.41
**6c**	20.94 ± 0.13	37.68 ± 0.20	77.05 ± 0.67	≥100	≥100
**6d**	31.56 ± 0.07	41.43 ± 0.79	33.77 ± 0.64	40.38 ± 0.01	41.39 ± 0.05
**7a**	≥100	≥100	≥100	≥100	≥100
**7b**	≥100	≥100	≥100	≥100	≥100
**7c**	≥100	≥100	45.12 ± 0.22	≥100	36.27 ± 0.14

* IC_50_ is the concentration of the compound that causes 50% cell death.

## Data Availability

Data are contained within the article and Supplementary Materials.

## Supplementary Materials

The following supporting information can be downloaded at: https://www.mdpi.com/article/10.3390/molecules29225360/s1, ^1^H and ^13^C NMR spectra of compounds.
