# Supplementary material for: N-Aryl Benzimidazole and Benzotriazole Derivatives and Their Hybrids as Cytotoxic Agents: Design, Synthesis and Structure–Activity Relationship Studies
Source: Molecules. 2024 Nov 14;29(22):5360. doi: 10.3390/molecules29225360 (PMC11596563; doi:10.3390/molecules29225360)
Supplement: Supplementary file 1 [file molecules-29-05360-s001.zip › molecules-3251227-supplementary.pdf]

Supplementary information

N-Aryl Benzimidazole and Benzotriazole Derivatives and Their Hybrids as Cytotoxic Agents: Design, Synthesis and Structure-Activity Relationship Studies

Yulia R. Aleksandrova[1], Natalia S. Nikolaeva[2], Inna A. Shagina[1,2], Karina D. Smirnova[3], Alla A. Zubishina[3], Alexander I. Chlopotinin[3], Artem N. Fakhrutdinov[4], Alexander L. Khokhlov[5], Roman S. Begunov[3,5,*] and Margarita E. Neganova [1*]

[1] Nesmeyanov Institute of Organoelement Compounds, Russian Academy of Sciences, 119991 Moscow, Russia; aleksyu@ineos.ac.ru (YuA), neganovam@ineos.ac.ru (MN)

[2] Institute of Physiologically Active Compounds at Federal Research Center of Problems of Chemical Physics and Medicinal Chemistry, Russian Academy of Sciences, Severnij pr. 1, 142432 Chernogolovka, Russia

[3] Faculty of Biology and Ecology, P. G. Demidov Yaroslavl State University of the Ministry of Science and Higher Education of the Russian Federation, 14 Sovetskaya Str., 150003 Yaroslavl, Russia begunov@uniyar.ac.ru (RB)

[4] N. D. Zelinsky Institute of Organic Chemistry, Russian Academy of Sciences, 47 Leninsky Prosp., 119991 Moscow, Russia

[5] Institute of Pharmacy, Yaroslavl State Medical University of the Ministry of Health of the Russian Federation, 5 Revolutsionnaya st., 150000 Yaroslavl, Russia

* Correspondence: begunov@uniyar.ac.ru (RB); neganovam@ineos.ac.ru (MN)

Table of Contents:

1. ^{1}H and ^{13}C NMR spectra of compounds **3a-3e** S2-S6
2. ^{1}H and ^{13}C NMR spectra of compounds **4a-4e** S7-S11
3. ^{1}H and ^{13}C NMR spectra of compounds **5a-5d** S12-S15
4. ^{1}H and ^{13}C NMR spectra of compounds **6a-6d** S16-S19
5. ^{1}H and ^{13}C NMR spectra of compounds **7a-7c** S20-S22
6. ^{1}H and ^{13}C NMR spectra of compounds **8a, 8b** S23-S24

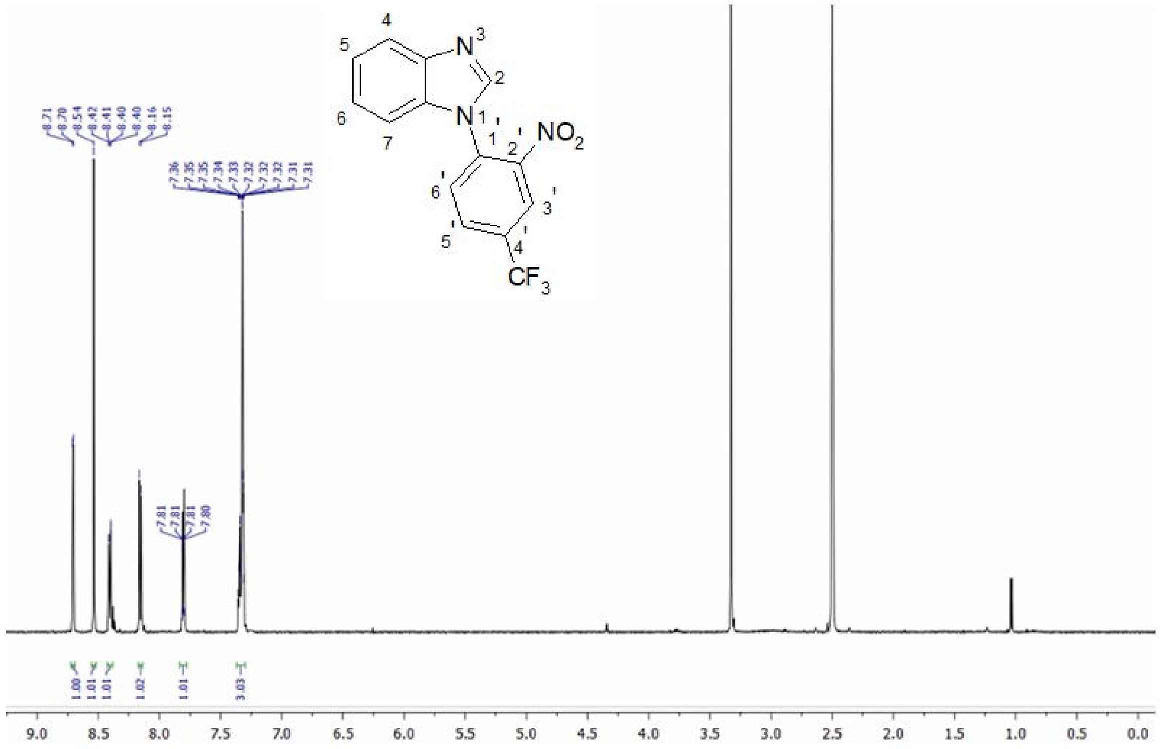

^{1}H NMR spectra of 1-(2-nitro-4-(trifluoromethyl)phenyl)-1*H*-benzimidazole (**3a**) (DMSO-d_6)

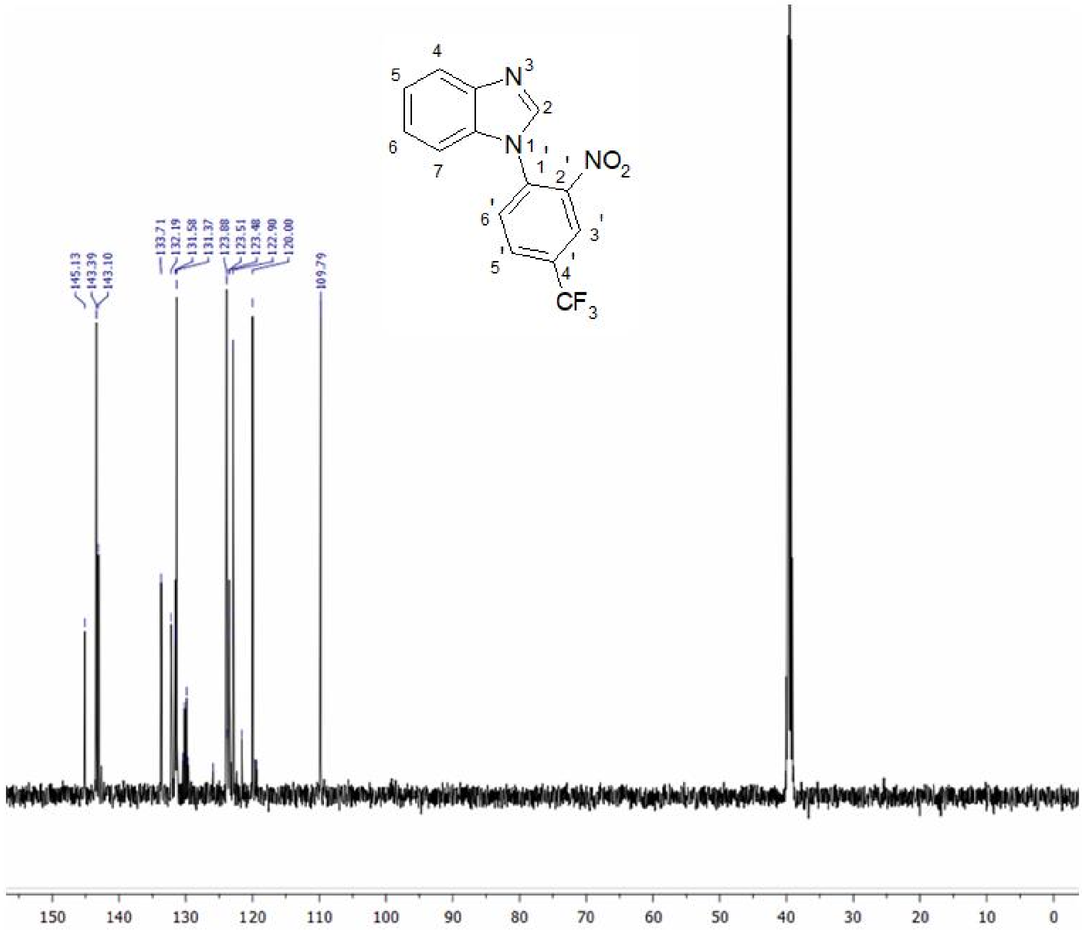

^{13}C NMR spectra of 1-(2-nitro-4-(trifluoromethyl)phenyl)-1*H*-benzimidazole (**3a**) (DMSO-d_6)

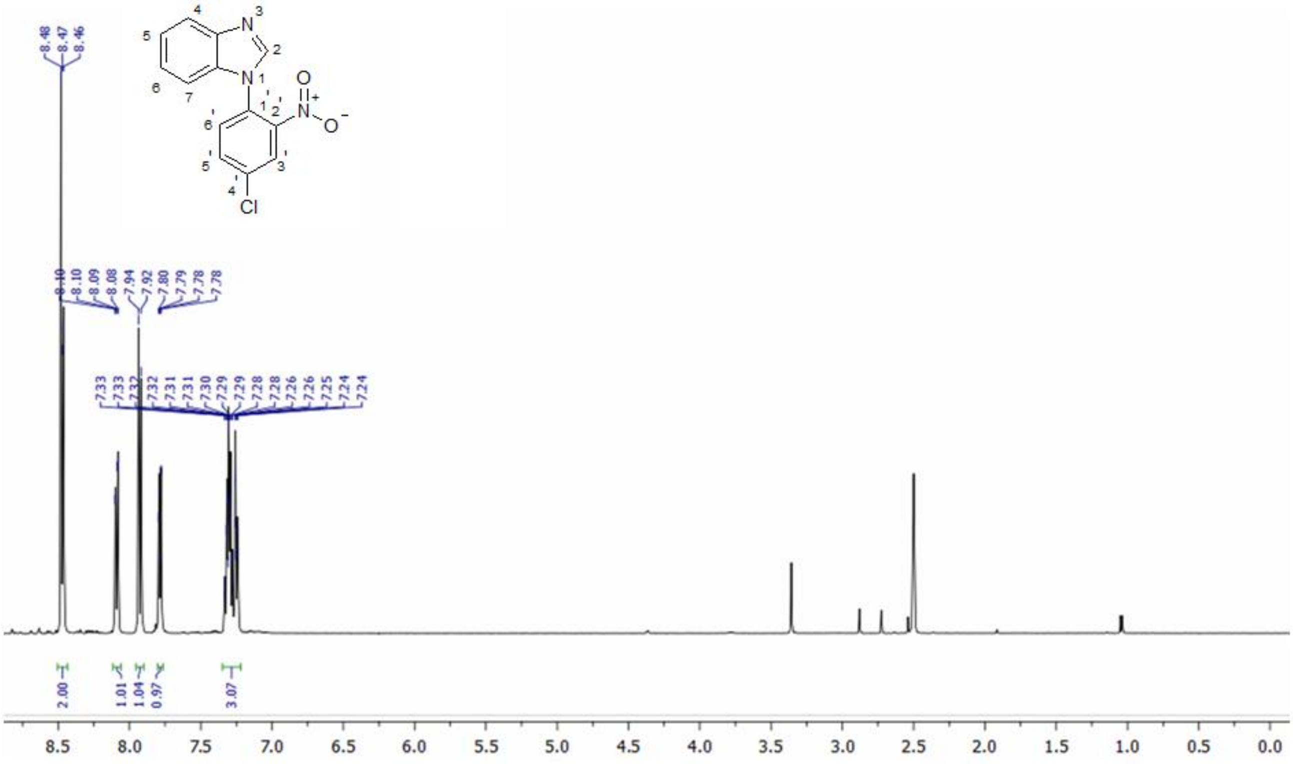

^{1}H NMR spectra of 1-(4-chloro-2-nitrophenyl)-1*H*-benzimidazole (**3b**) (DMSO-d_6)

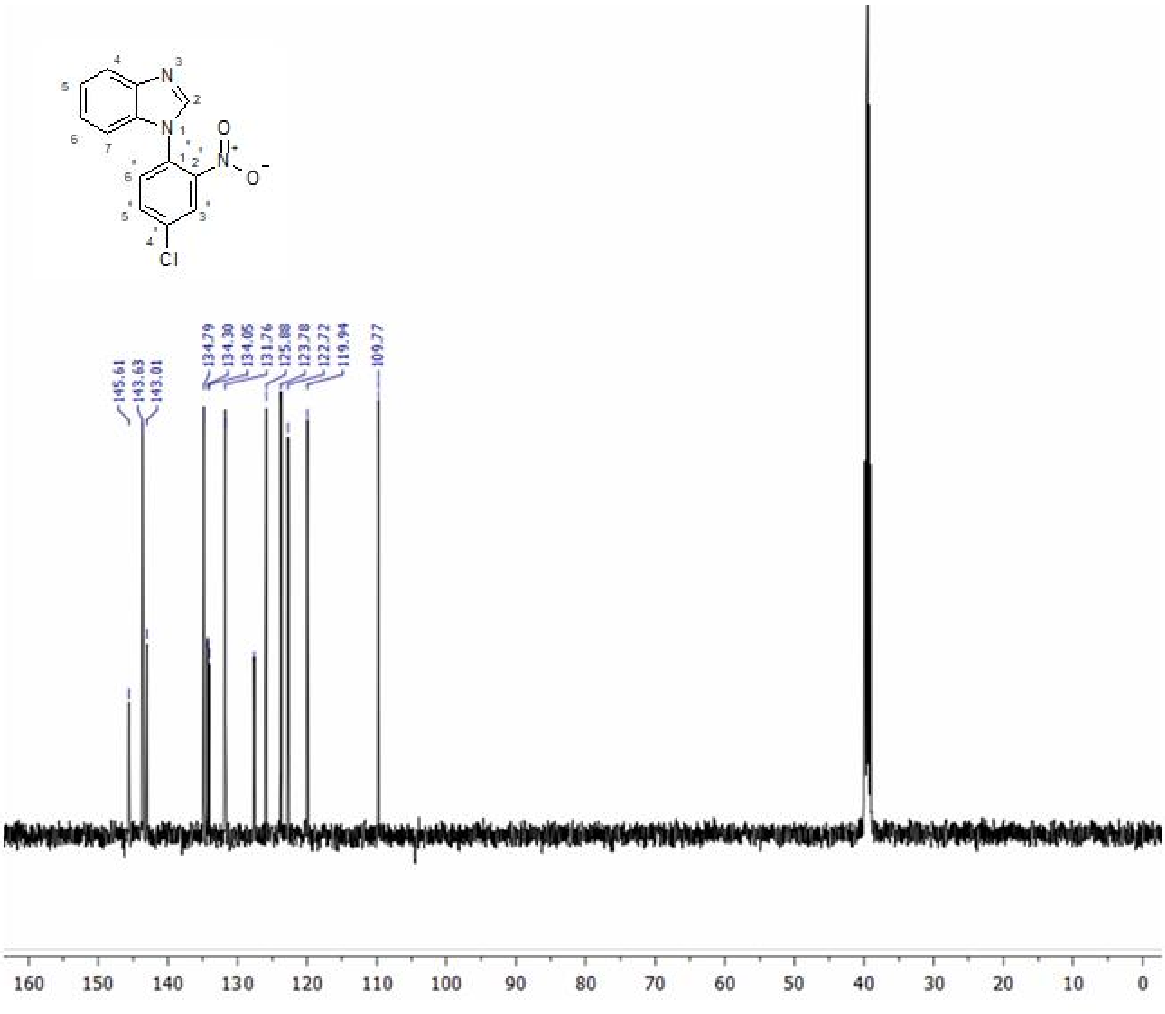

^{13}C NMR spectra of 1-(4-chloro-2-nitrophenyl)-1*H*-benzimidazole (**3b**) (DMSO-d_6)

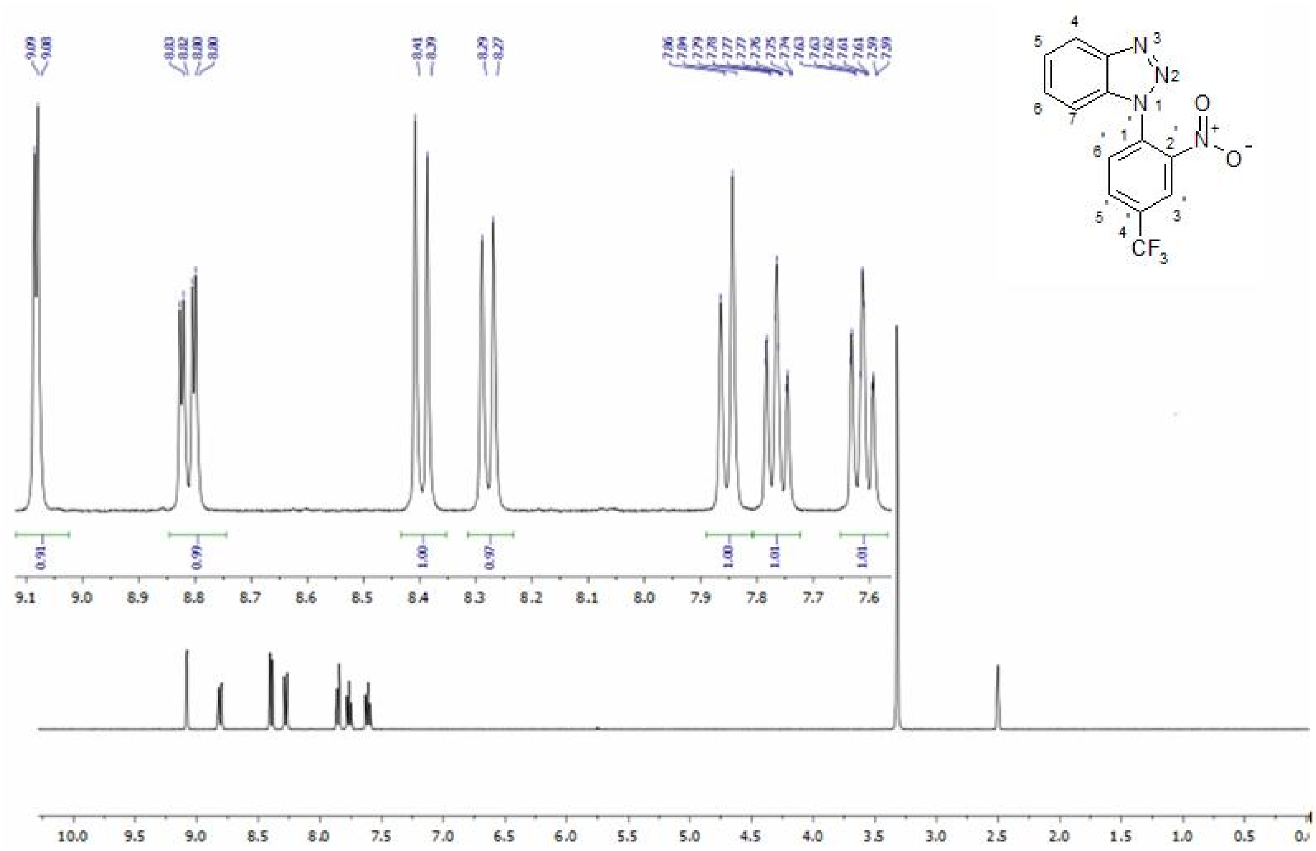

^{1}H NMR spectra of 1-[2-nitro-4-(trifluoromethyl)phenyl]-1*H*-benzotriazole (**3c**) (DMSO-d_6)

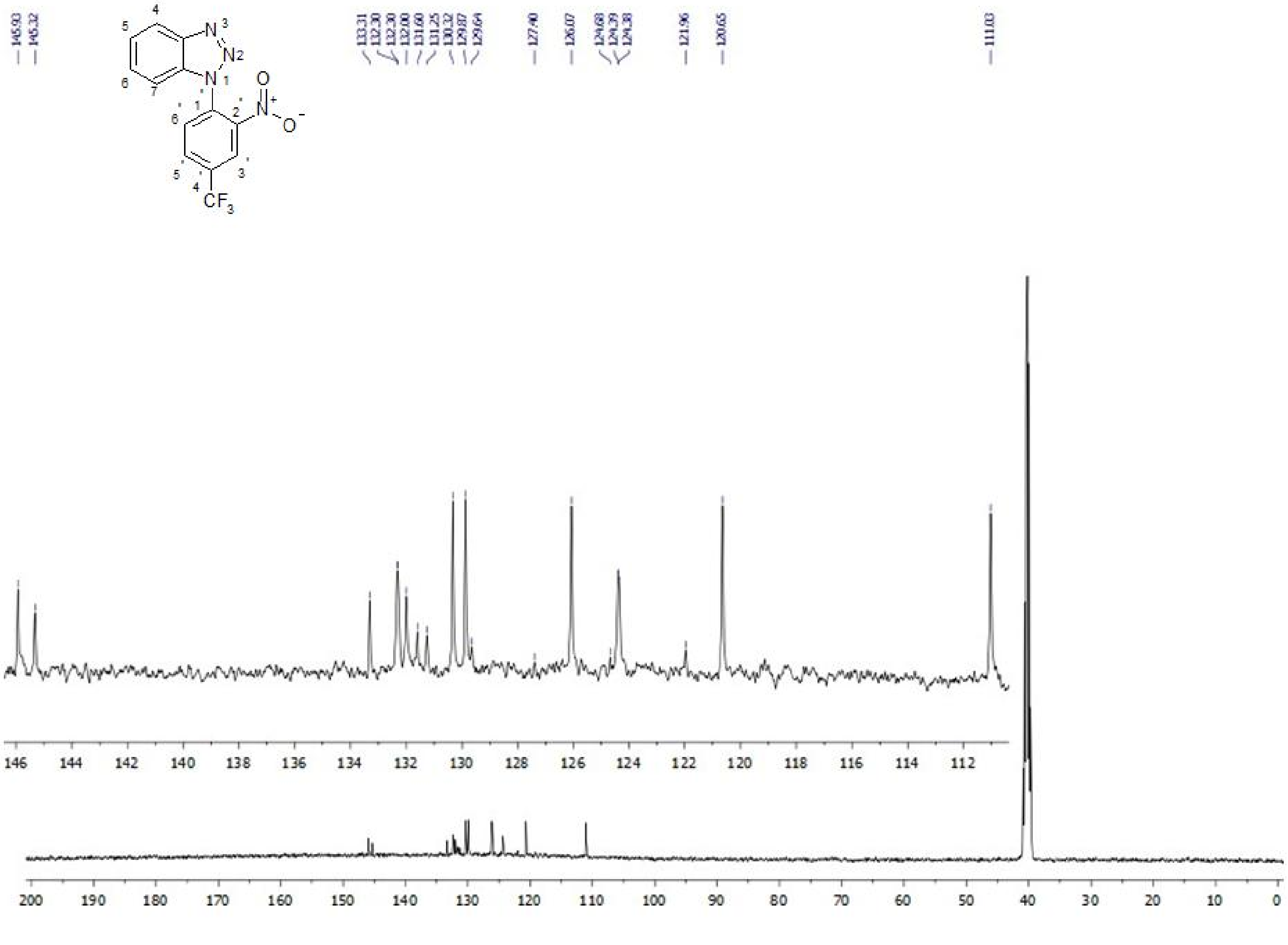

^{13}C NMR spectra of 1-[2-nitro-4-(trifluoromethyl)phenyl]-1*H*-benzotriazole (**3c**) (DMSO-d_6)

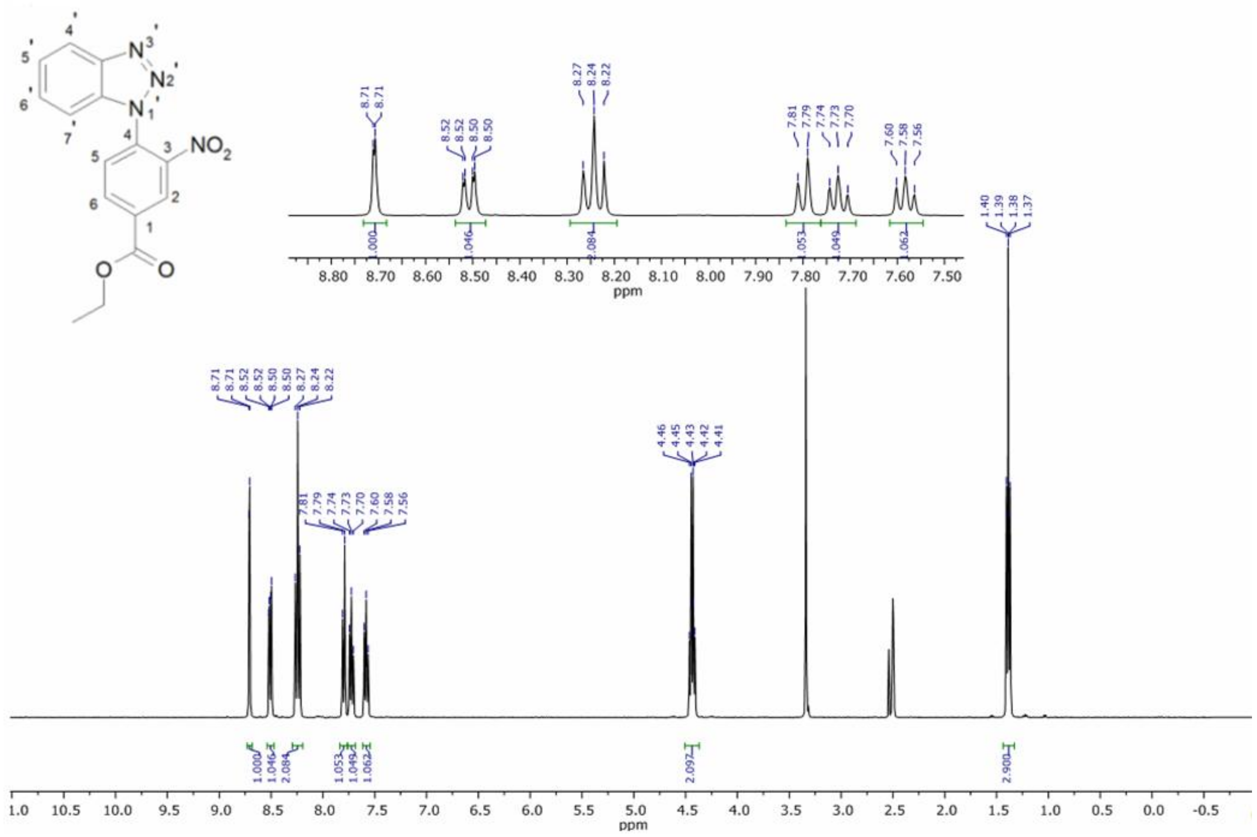

^{1}H NMR spectra of ethyl 4-(1*H*-benzotriazol-1-yl)-3-nitrobenzoate (**3d**) (DMSO-d_6)

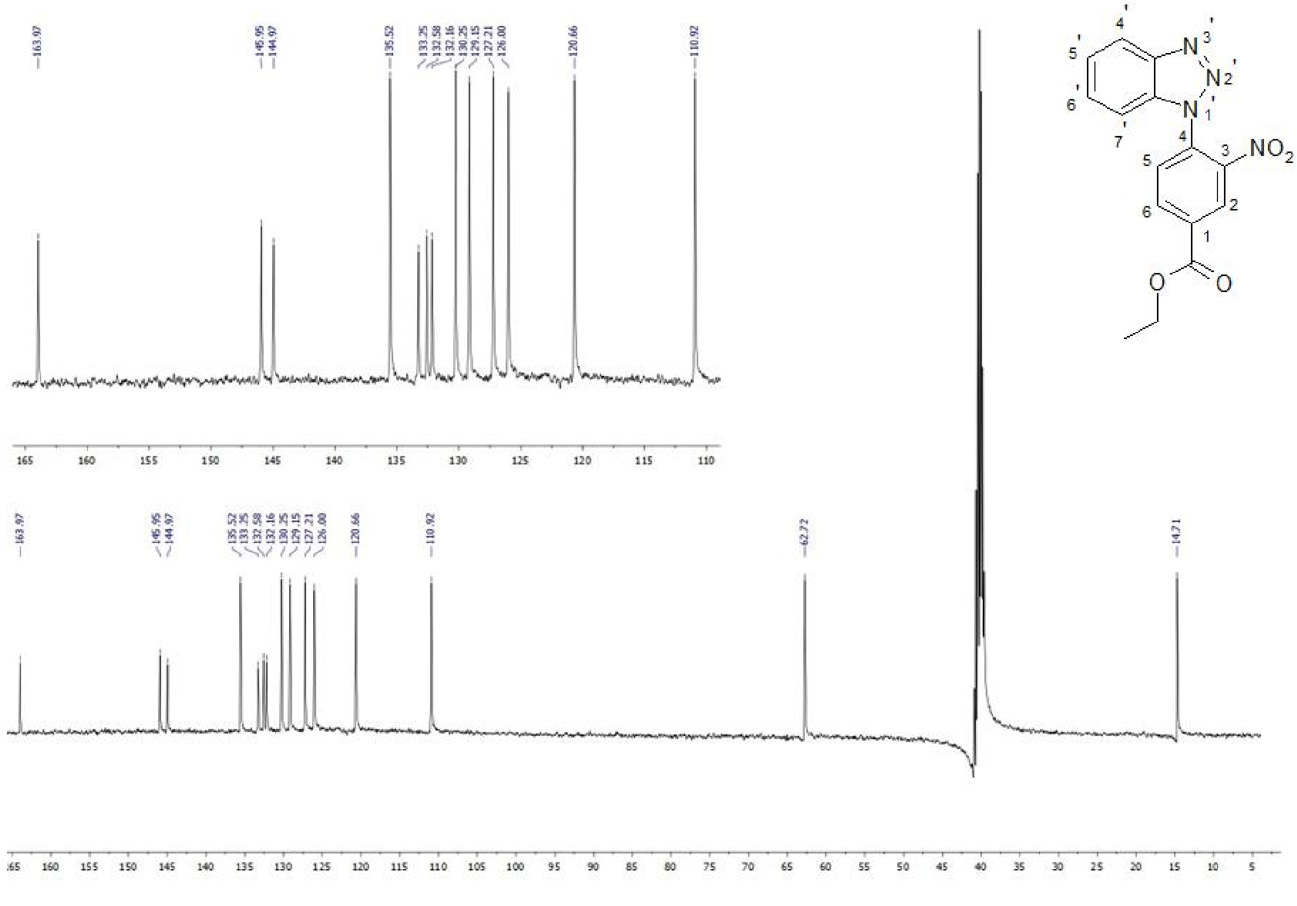

^{13}C NMR spectra of ethyl 4-(1*H*-benzotriazol-1-yl)-3-nitrobenzoate (**3d**) (DMSO-d_6)

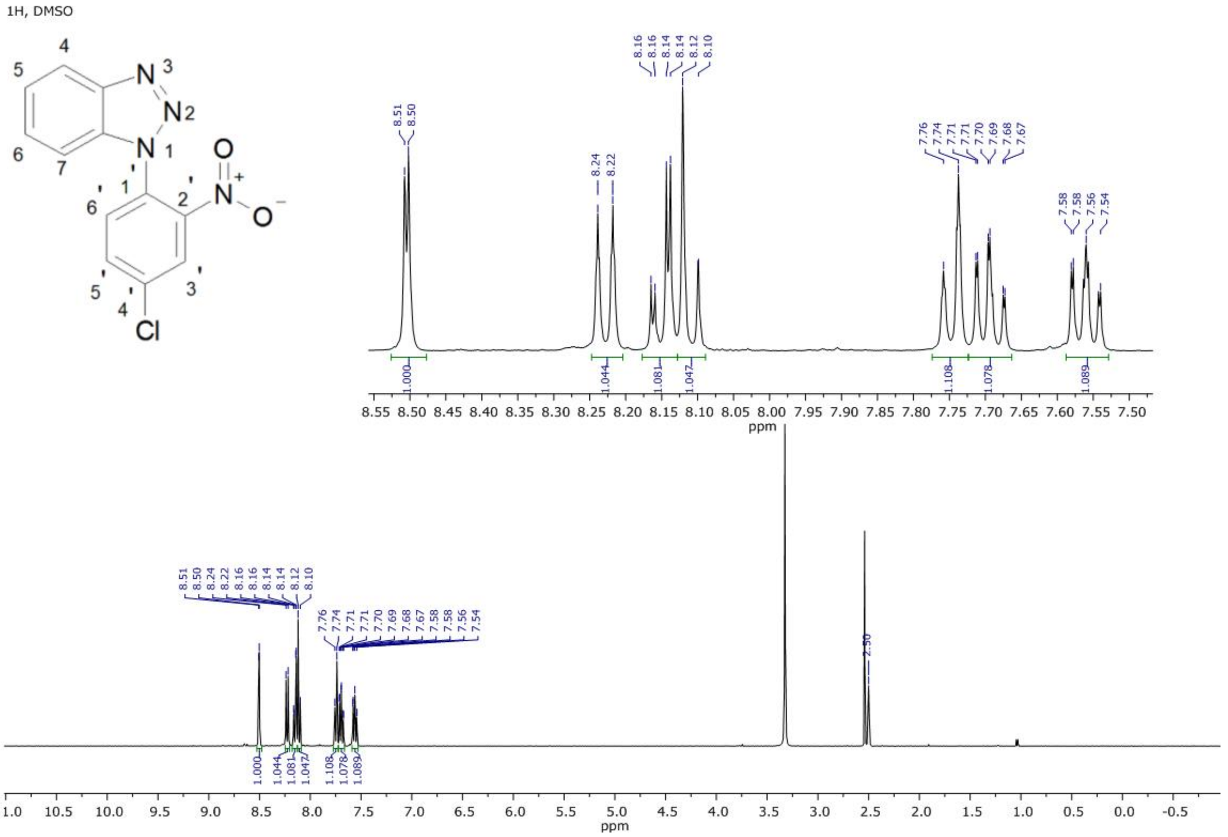

^{1}H NMR spectra of 1-(4-chloro-2-nitrophenyl)-1*H*-benzotriazole (**3e**) (DMSO-d_6)

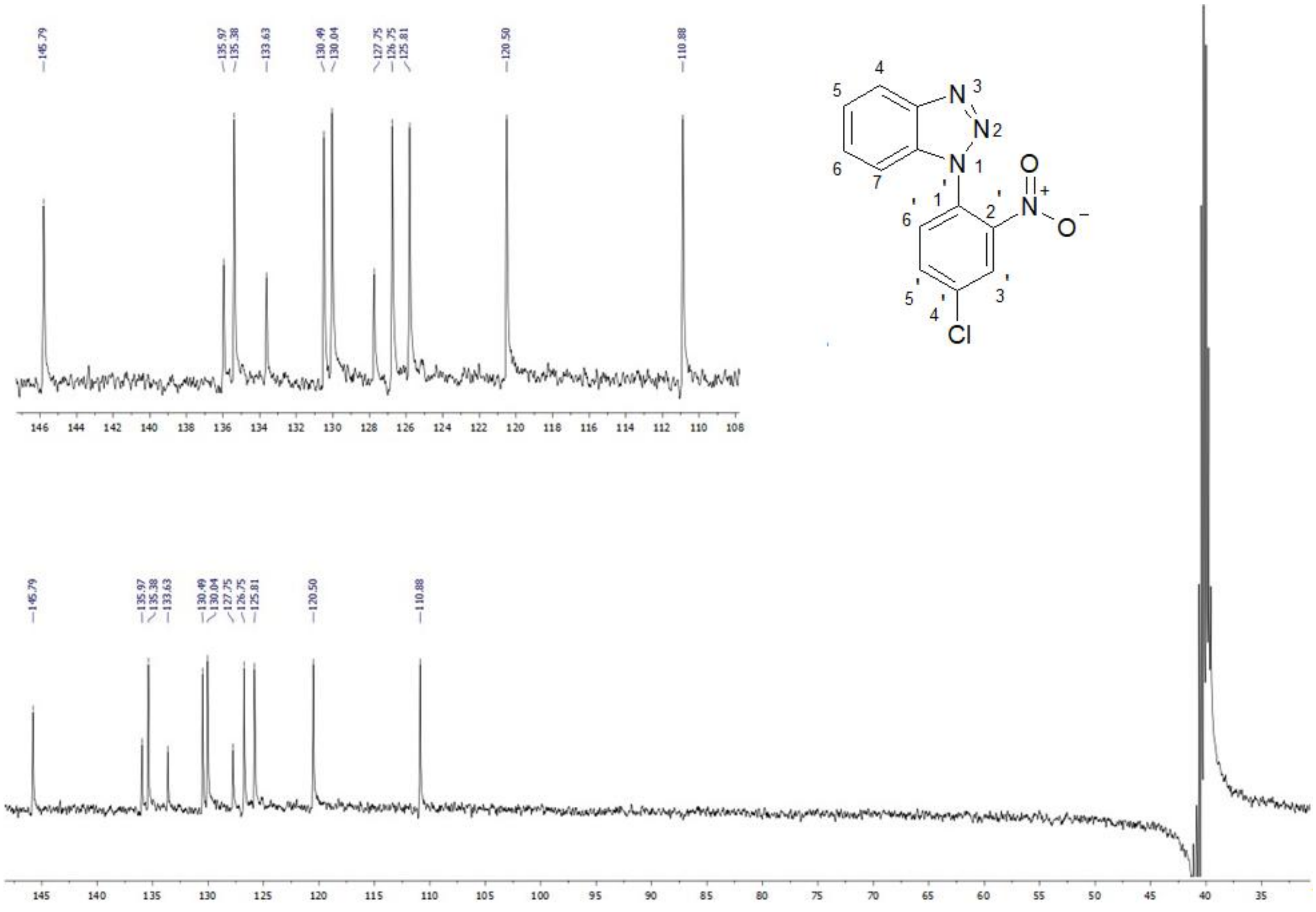

^{13}C NMR spectra of 1-(4-chloro-2-nitrophenyl)-1*H*-benzotriazole (**3e**) (DMSO-d_6)

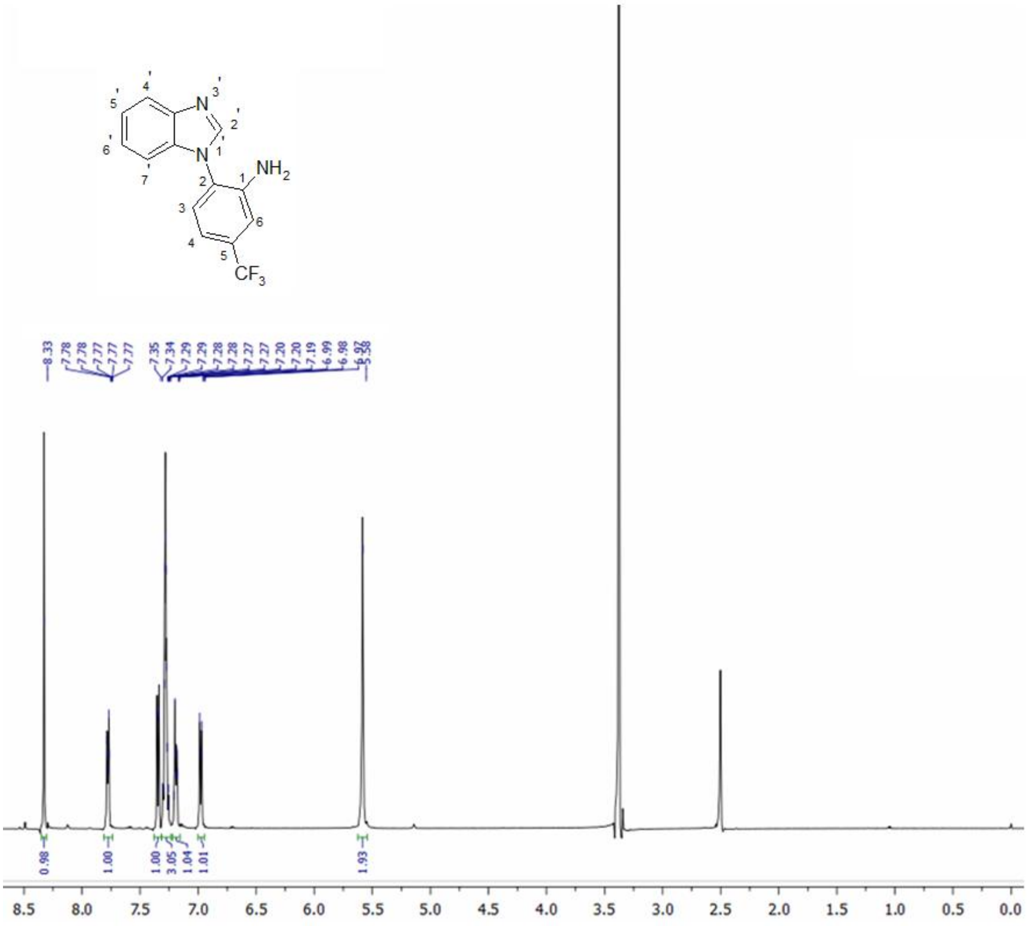

^{1}H NMR spectra of 2-(1*H*-benzimidazol-1-yl)-5-(trifluoromethyl)aniline **(4a)** (DMSO-d_6)

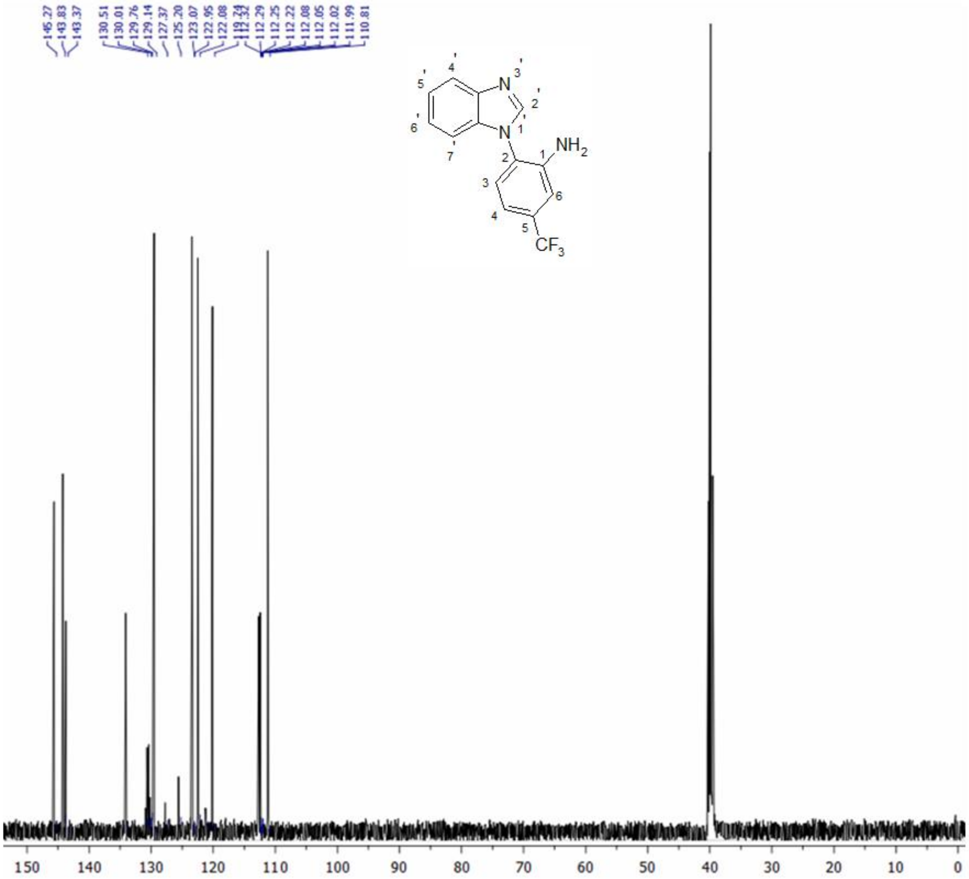

^{13}C NMR spectra of 2-(1*H*-benzimidazol-1-yl)-5-(trifluoromethyl)aniline **(4a)** (DMSO-d_6)

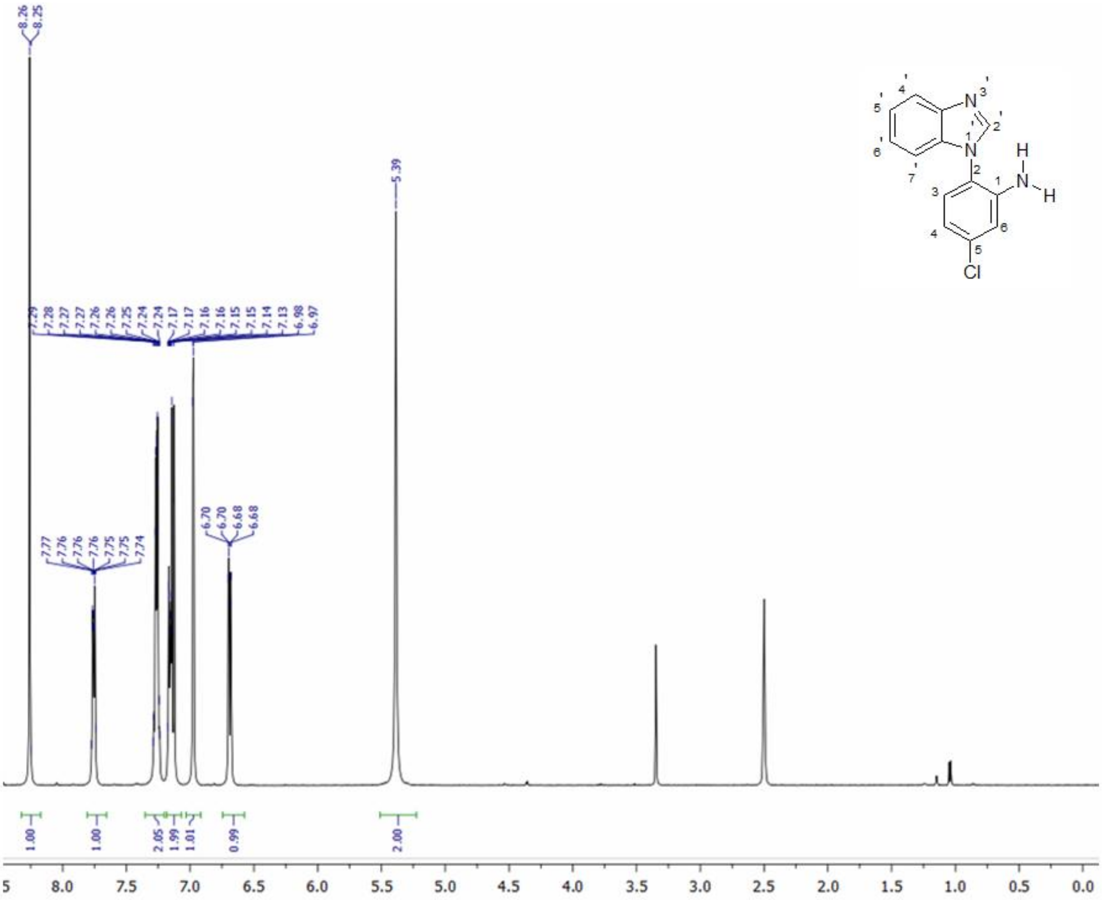

^{1}H NMR spectra of 2-(1*H*-benzimidazol-1-yl)-5-chloroaniline **(4b)** (DMSO-d_6)

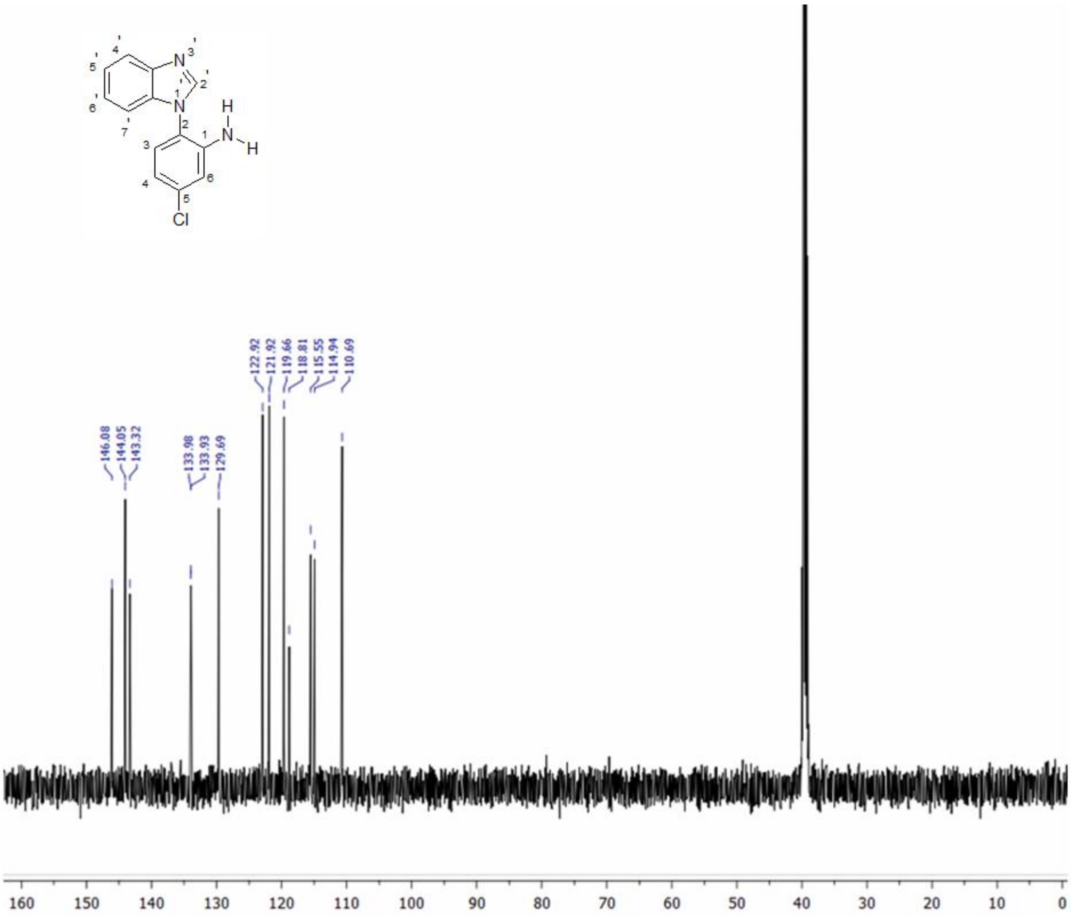

^{13}C NMR spectra of 2-(1*H*-benzimidazol-1-yl)-5-chloroaniline **(4b)** (DMSO-d_6)

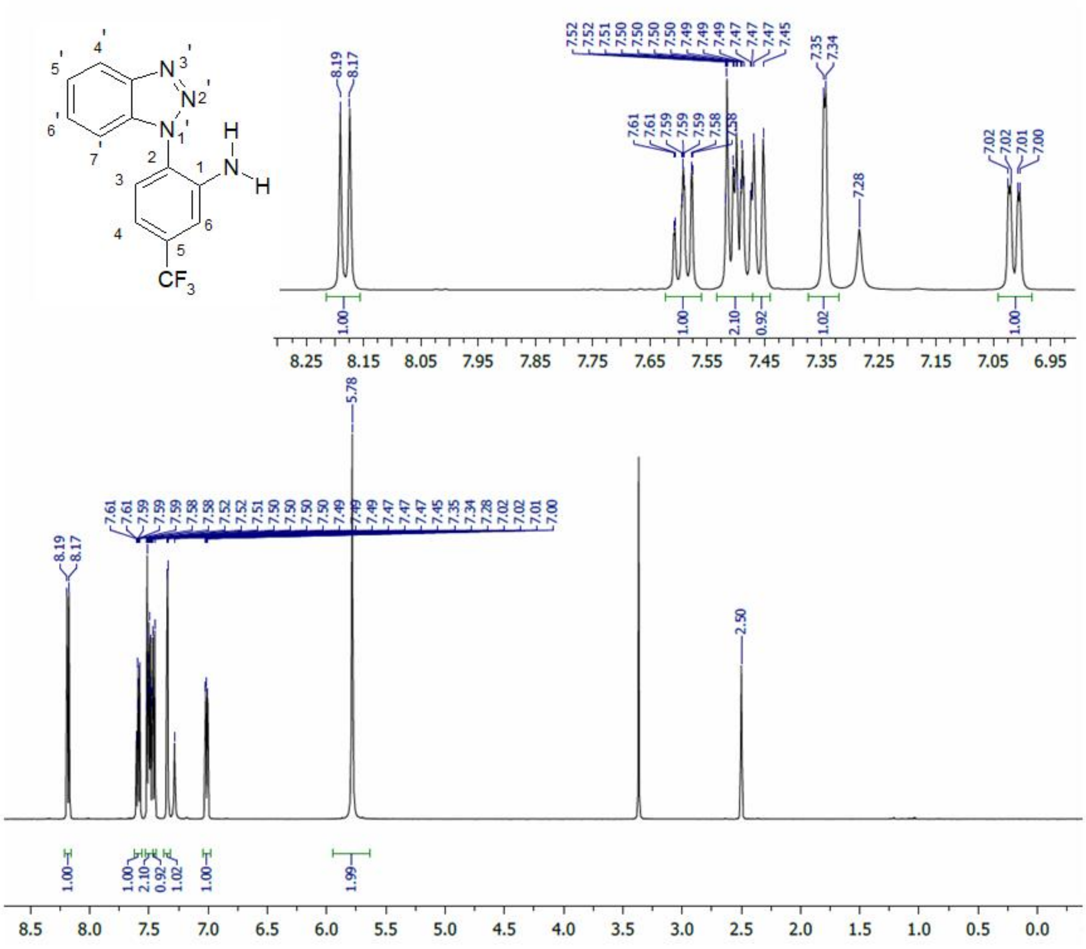

^{1}H NMR spectra of 2-(1*H*-benzotriazol-1-yl)-5-(trifluoromethyl)aniline **(4c)** (DMSO-d_6)

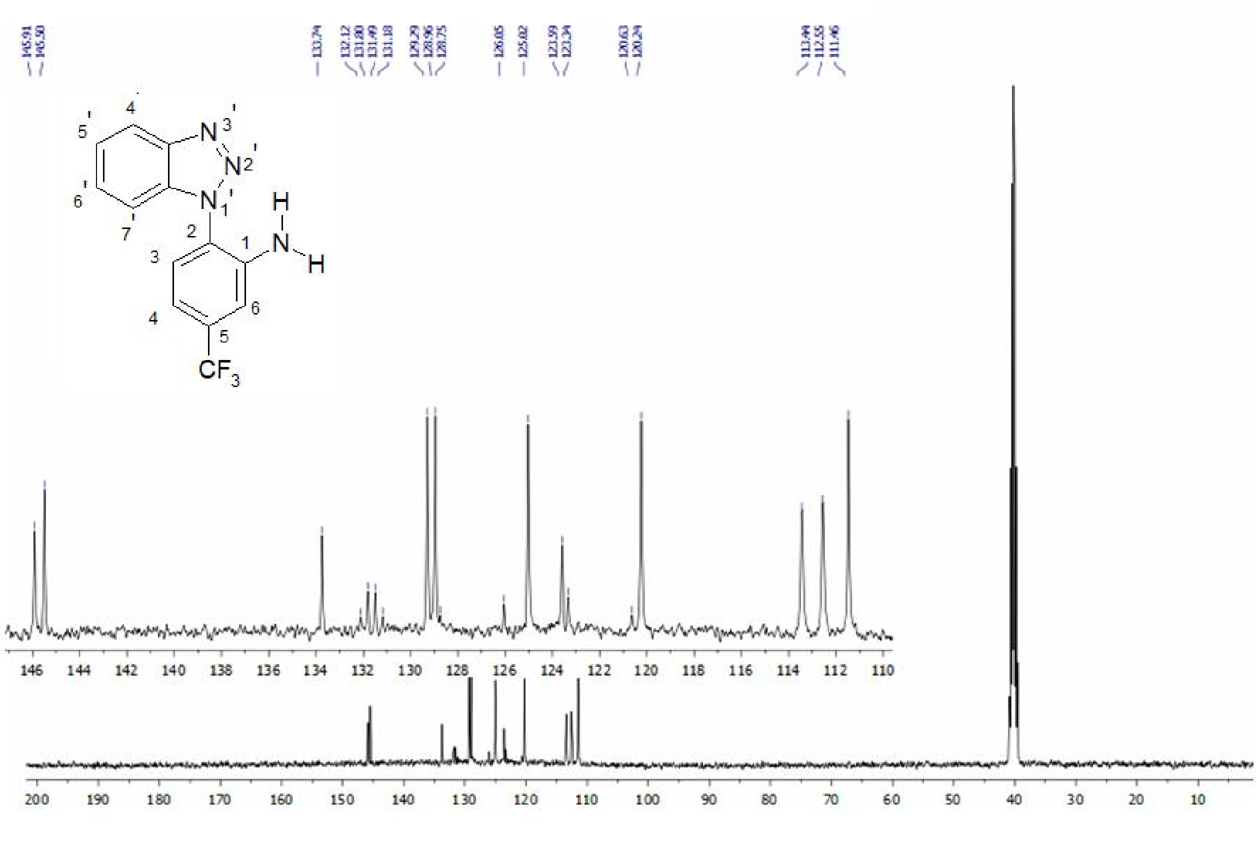

^{13}C NMR spectra of 2-(1*H*-benzotriazol-1-yl)-5-(trifluoromethyl)aniline **(4c)** (DMSO-d_6)

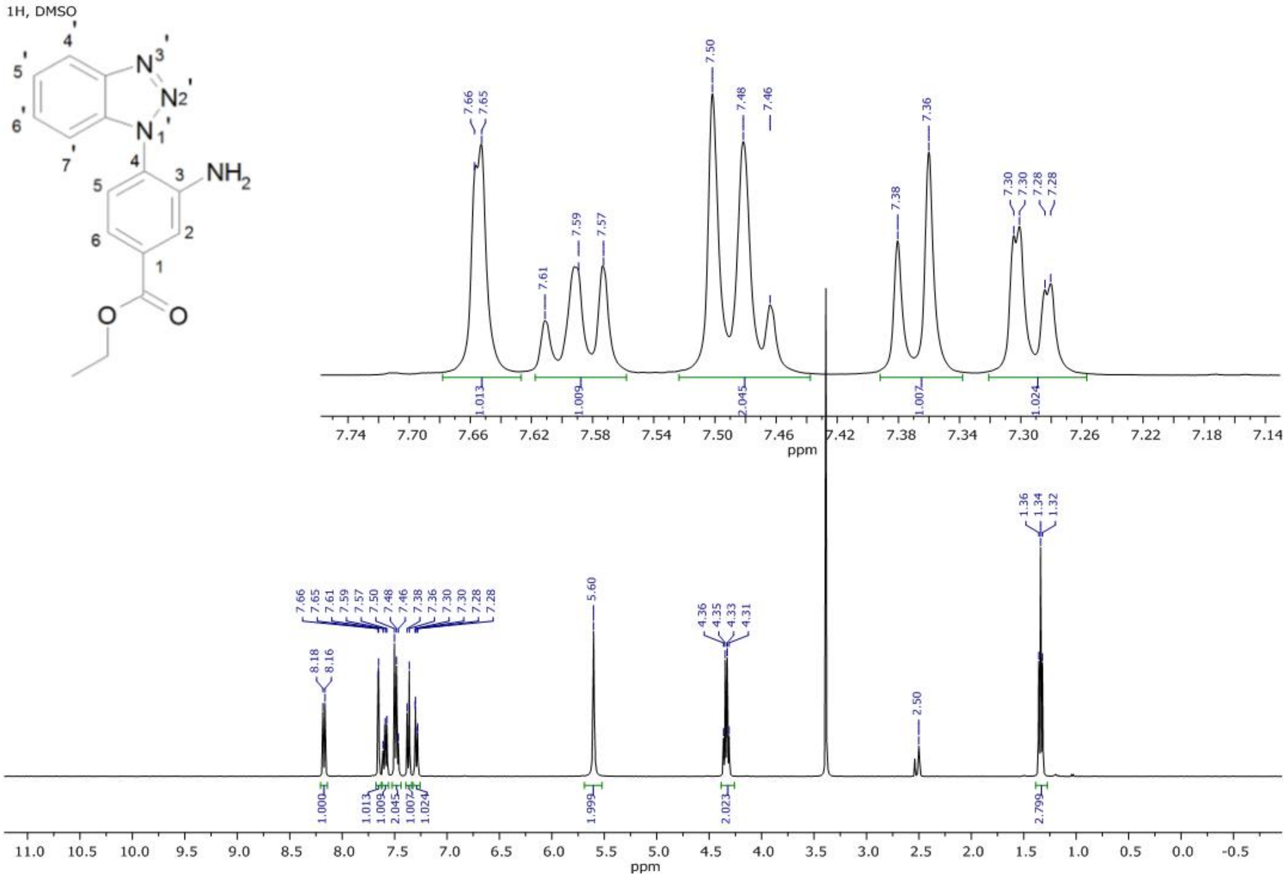

^{1}H NMR spectra of ethyl 3-amino-4-(1*H*-benzotriazol-1-yl)benzoate (**4d**) (DMSO-*d*$_6$)

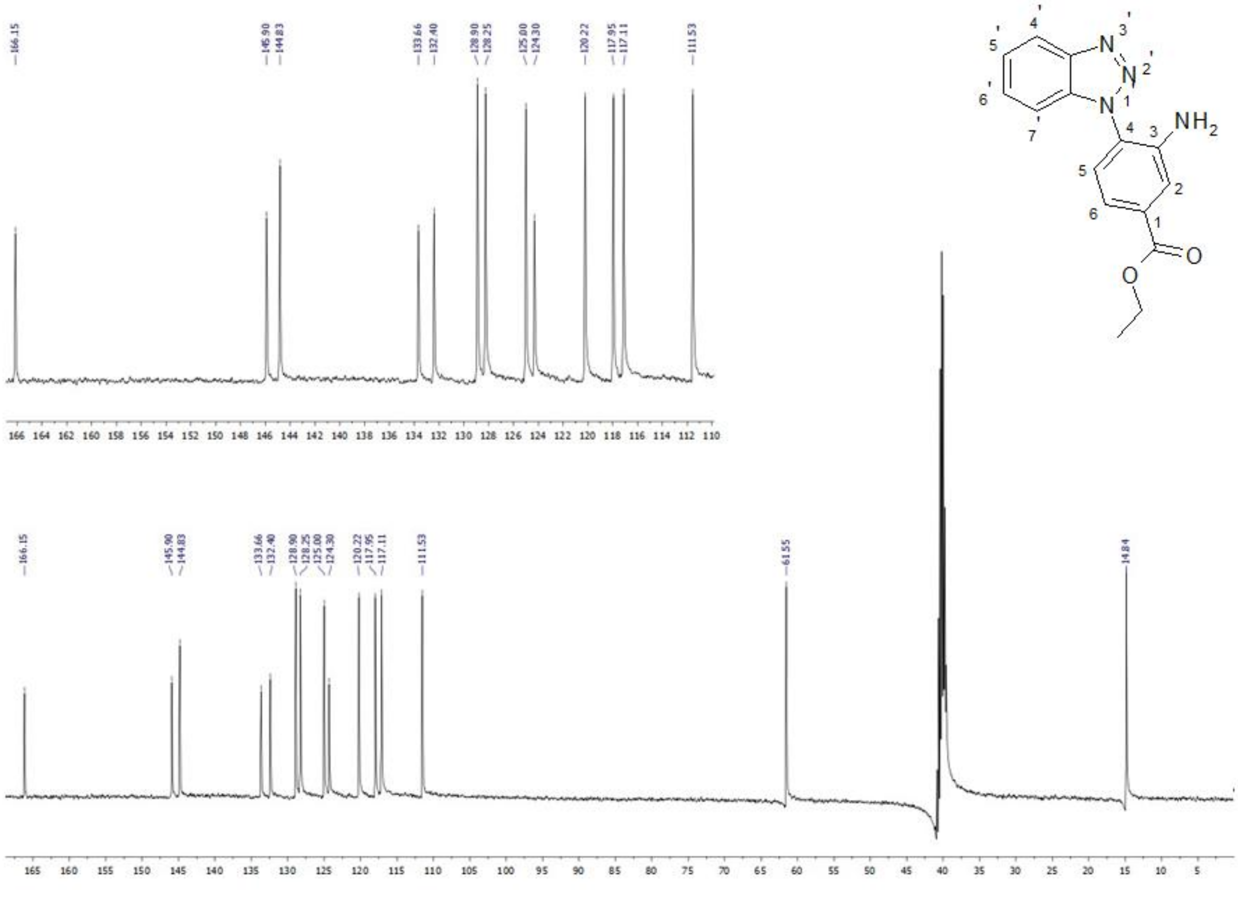

^{13}C NMR spectra of ethyl 3-amino-4-(1*H*-benzotriazol-1-yl)benzoate (**4d**) (DMSO-*d*$_6$)

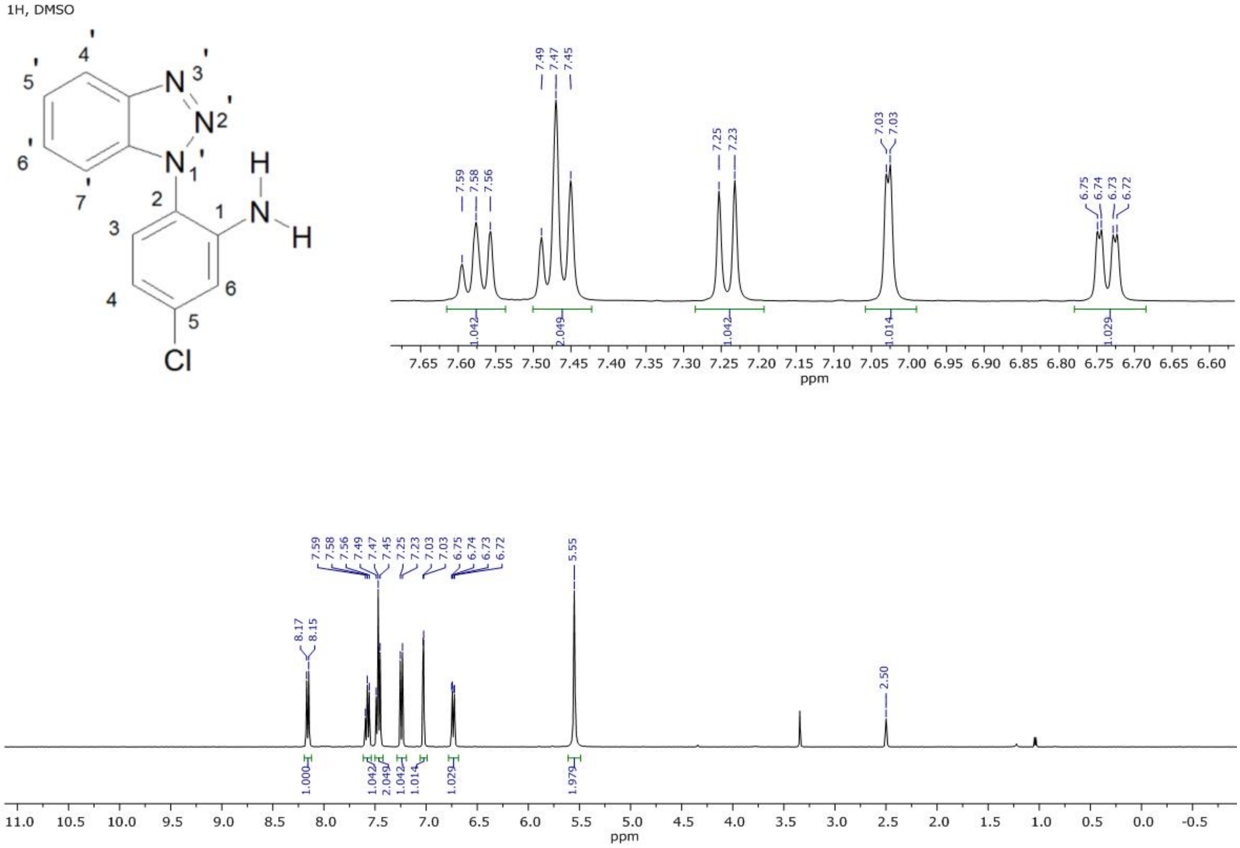

^{1}H NMR spectra of 2-(1*H*-benzotriazol-1-yl)-5-chloroaniline (**4e**) (DMSO-d_6)

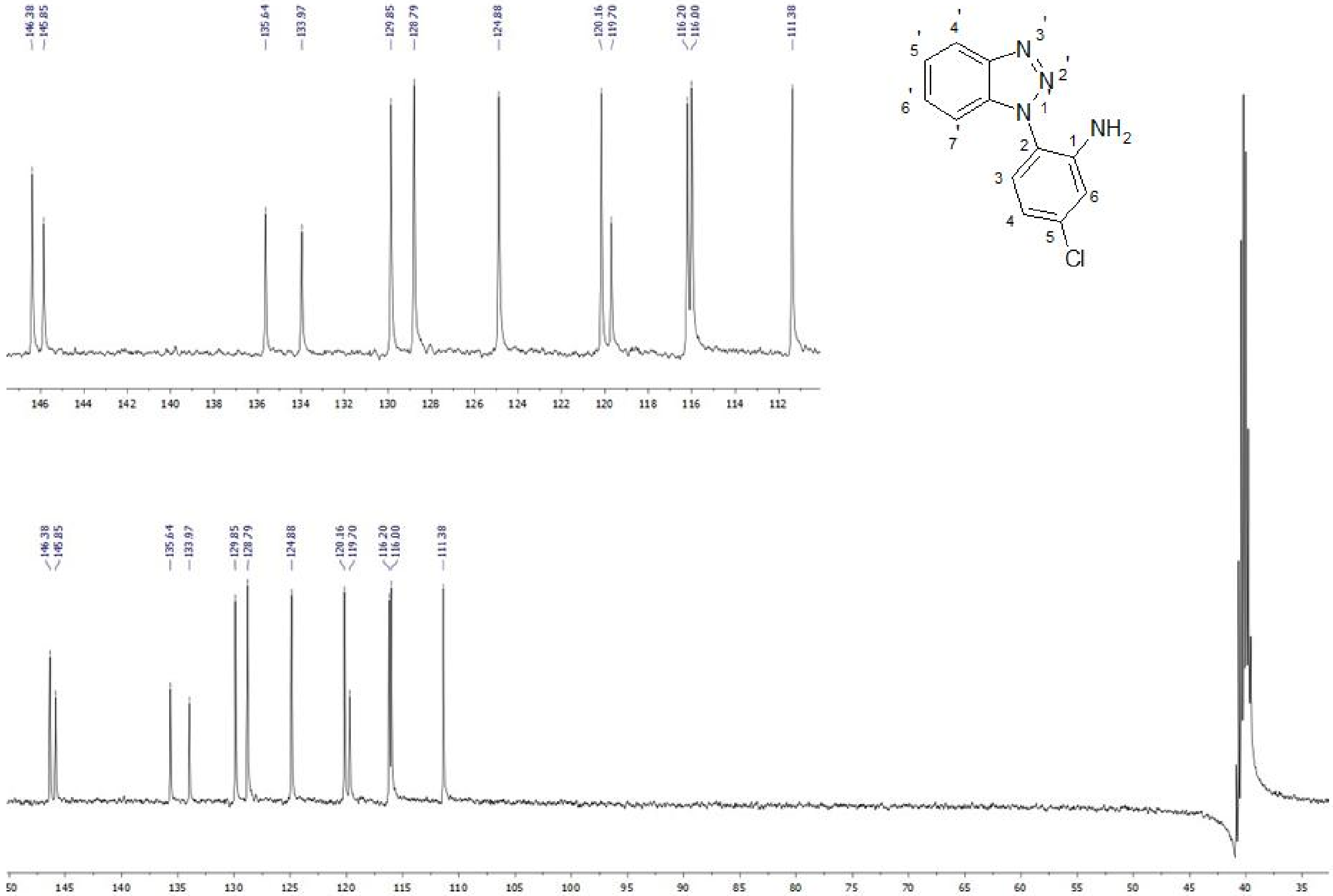

^{13}C NMR spectra of 2-(1*H*-benzotriazol-1-yl)-5-chloroaniline (**4e**) (DMSO-d_6)

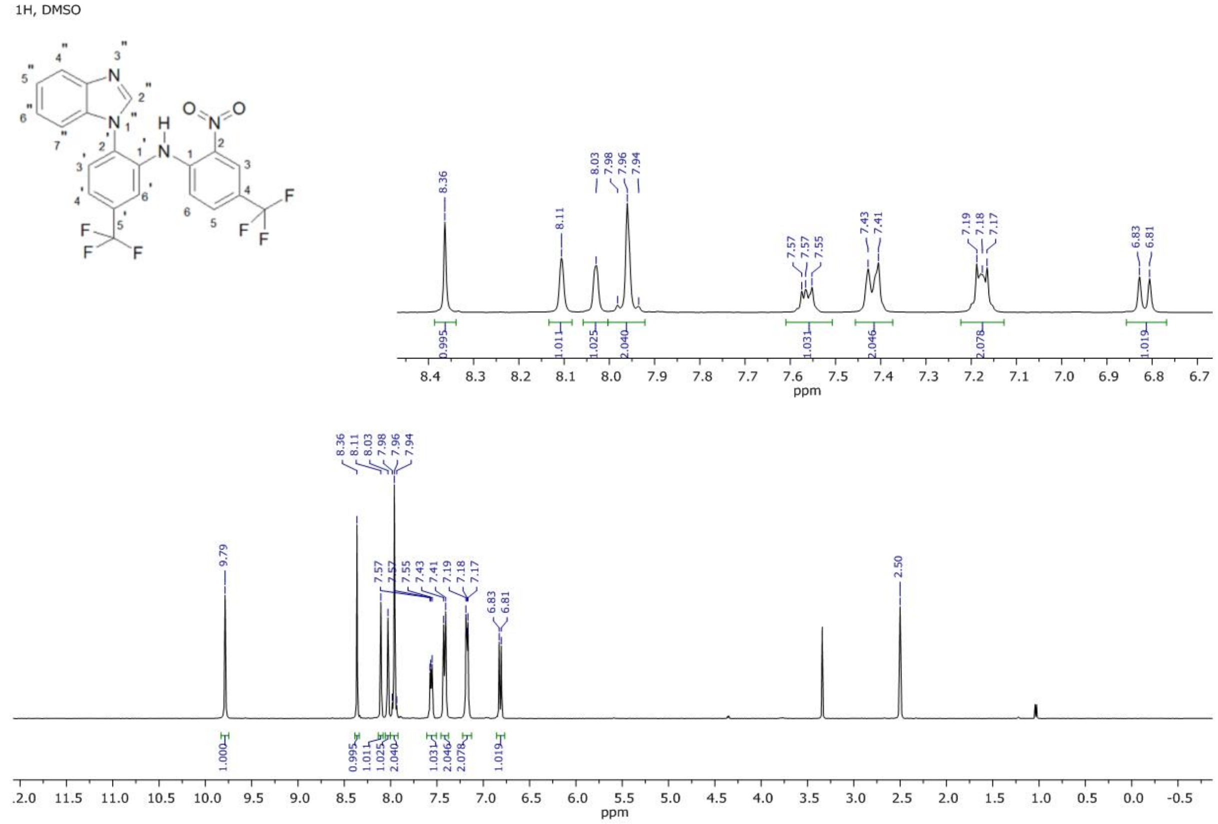

^{1}H NMR spectra of *N*-[2-(1*H*-benzimidazol-1-yl)-5-(trifluoromethyl)phenyl]-2-nitro-4-(trifluoromethyl)aniline (**5a**) (DMSO-d_6)

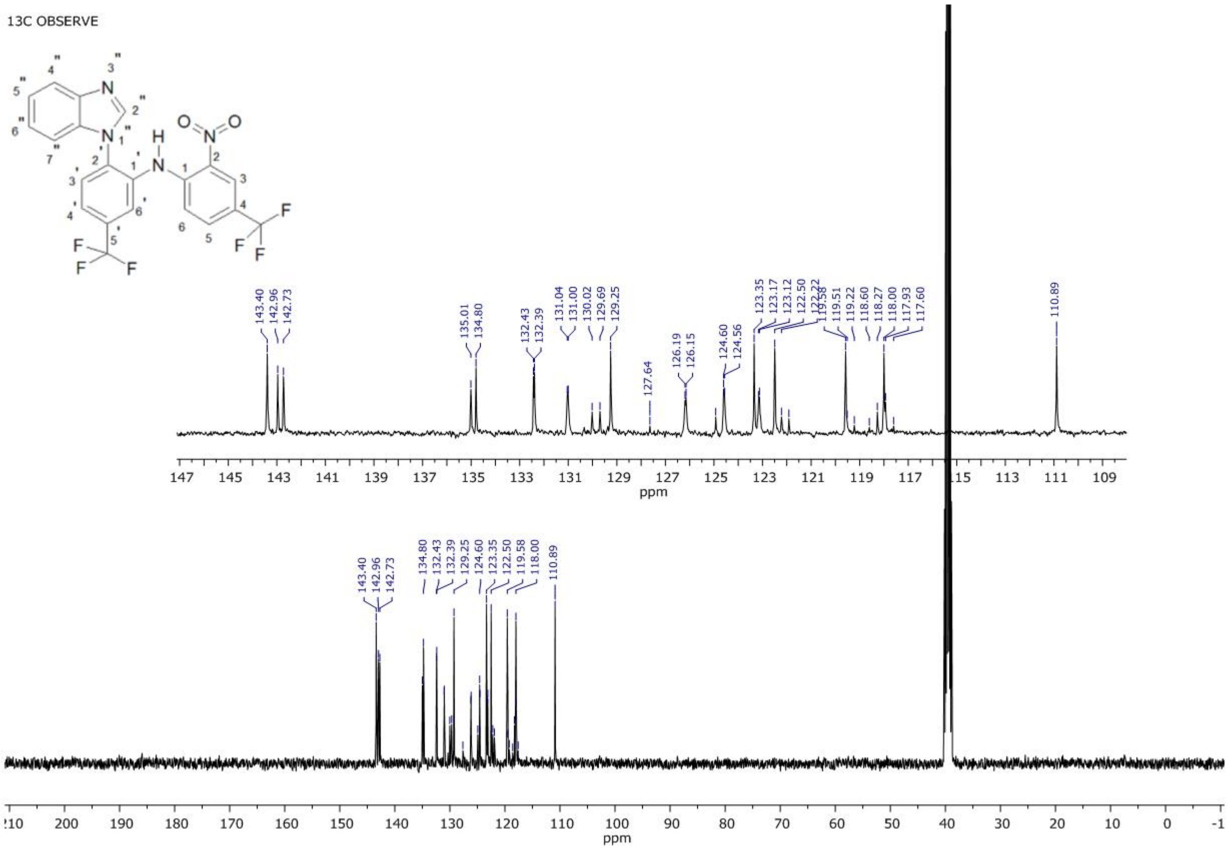

^{13}C NMR spectra of *N*-[2-(1*H*-benzimidazol-1-yl)-5-(trifluoromethyl)phenyl]-2-nitro-4-(trifluoromethyl)aniline (**5a**) (DMSO-d_6)

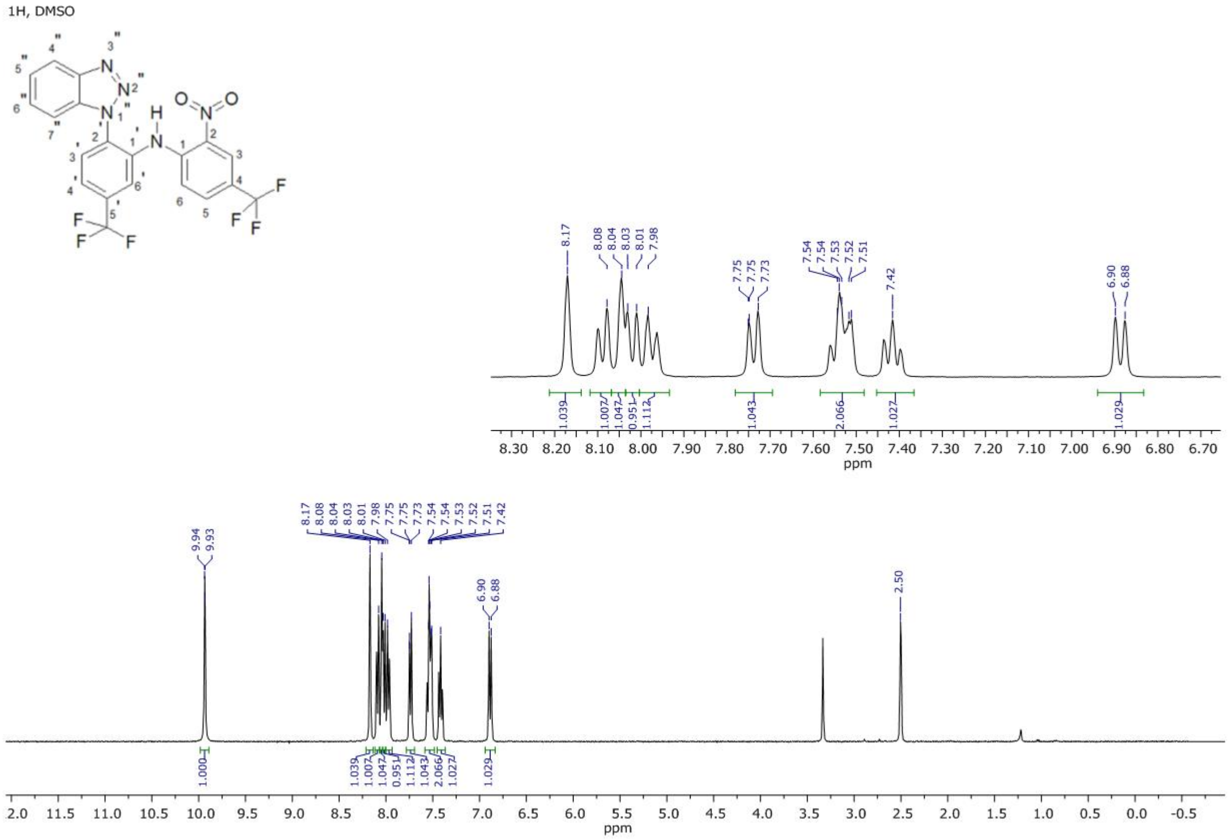

^{1}H NMR spectra of N-[2-(1*H*-benzotriazol-1-yl)-5-(trifluoromethyl)phenyl]-2-nitro-4-(trifluoromethyl)aniline (**5b**) (DMSO-d_6)

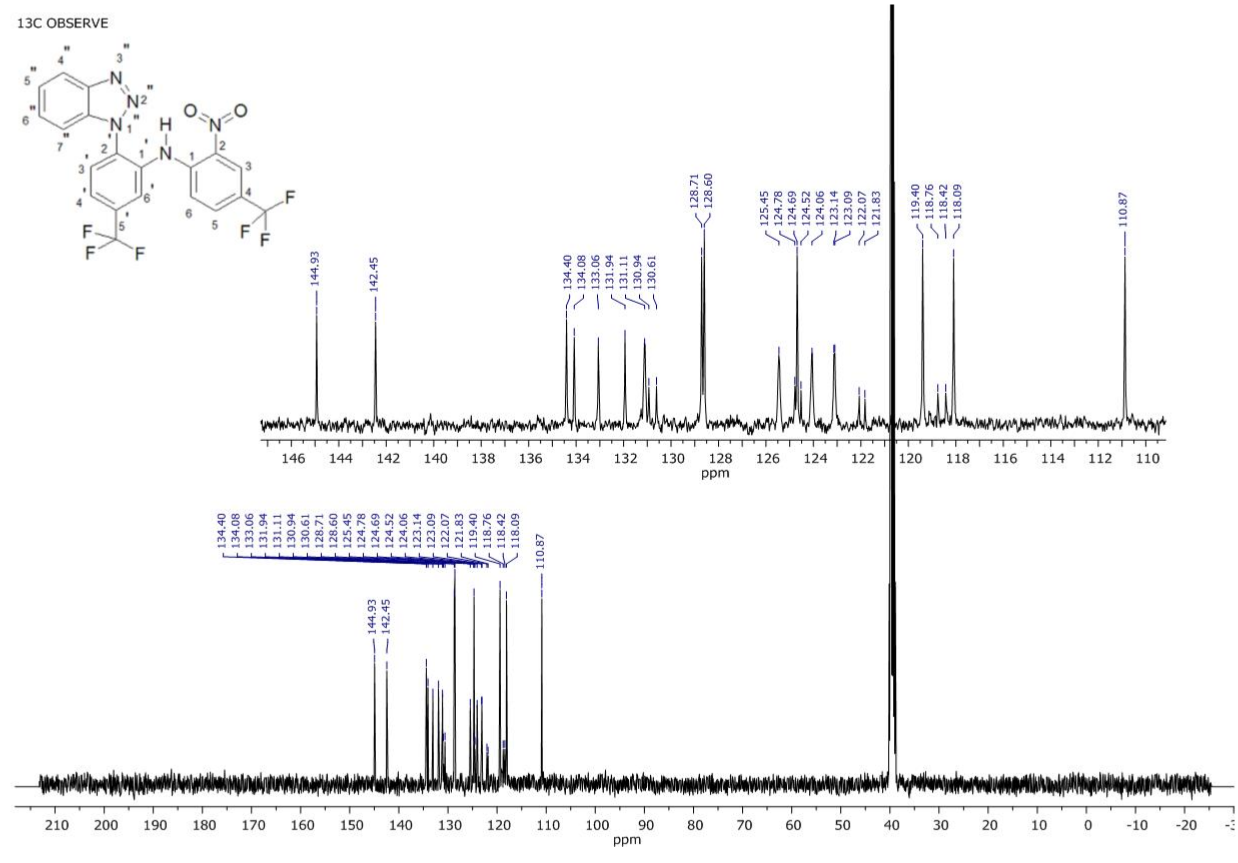

^{13}C NMR spectra of N-[2-(1*H*-benzotriazol-1-yl)-5-(trifluoromethyl)phenyl]-2-nitro-4-(trifluoromethyl)aniline (**5b**) (DMSO-d_6)

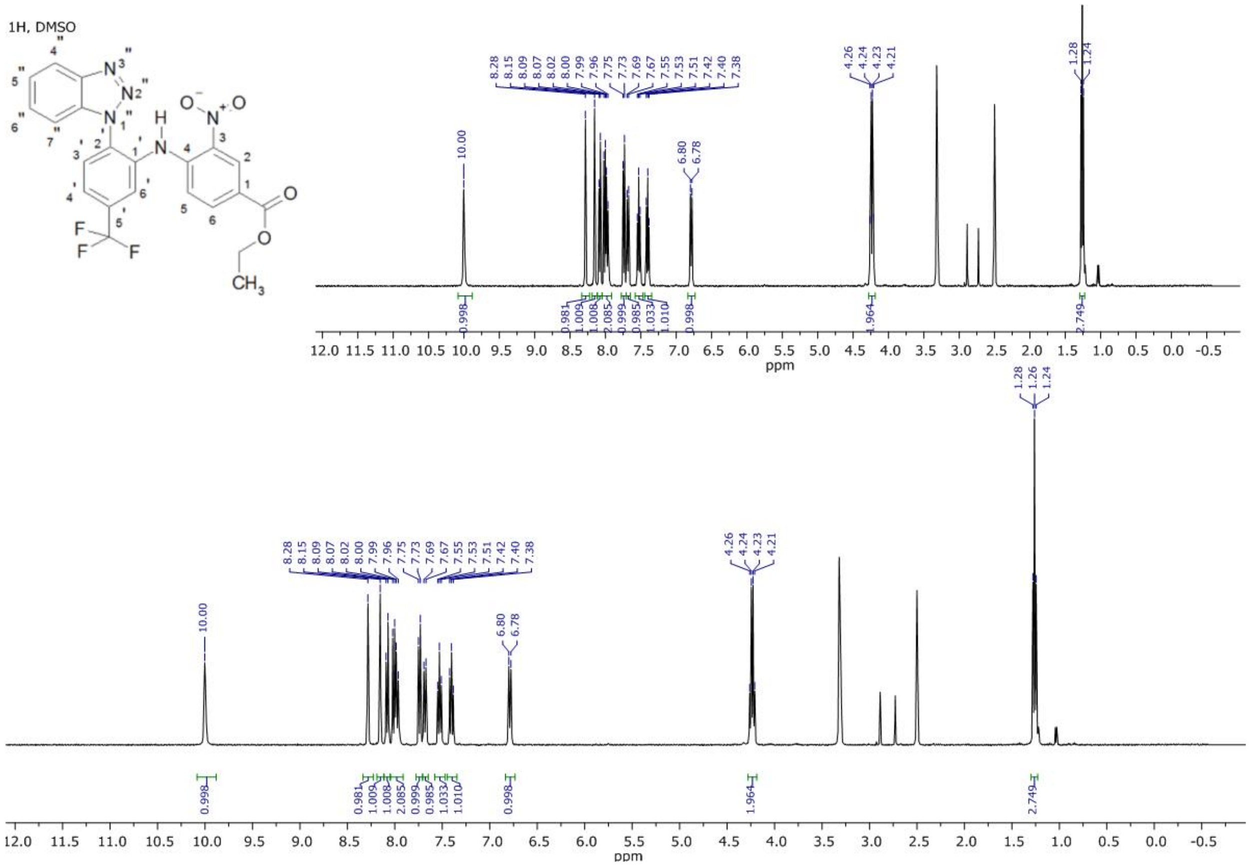

^{1}H NMR spectra of ethyl 4-[2-(1*H*-benzotriazol-1-yl)-5-(trifluoromethyl)anilino]-3-nitrobenzoate (**5c**) (DMSO-d_6)

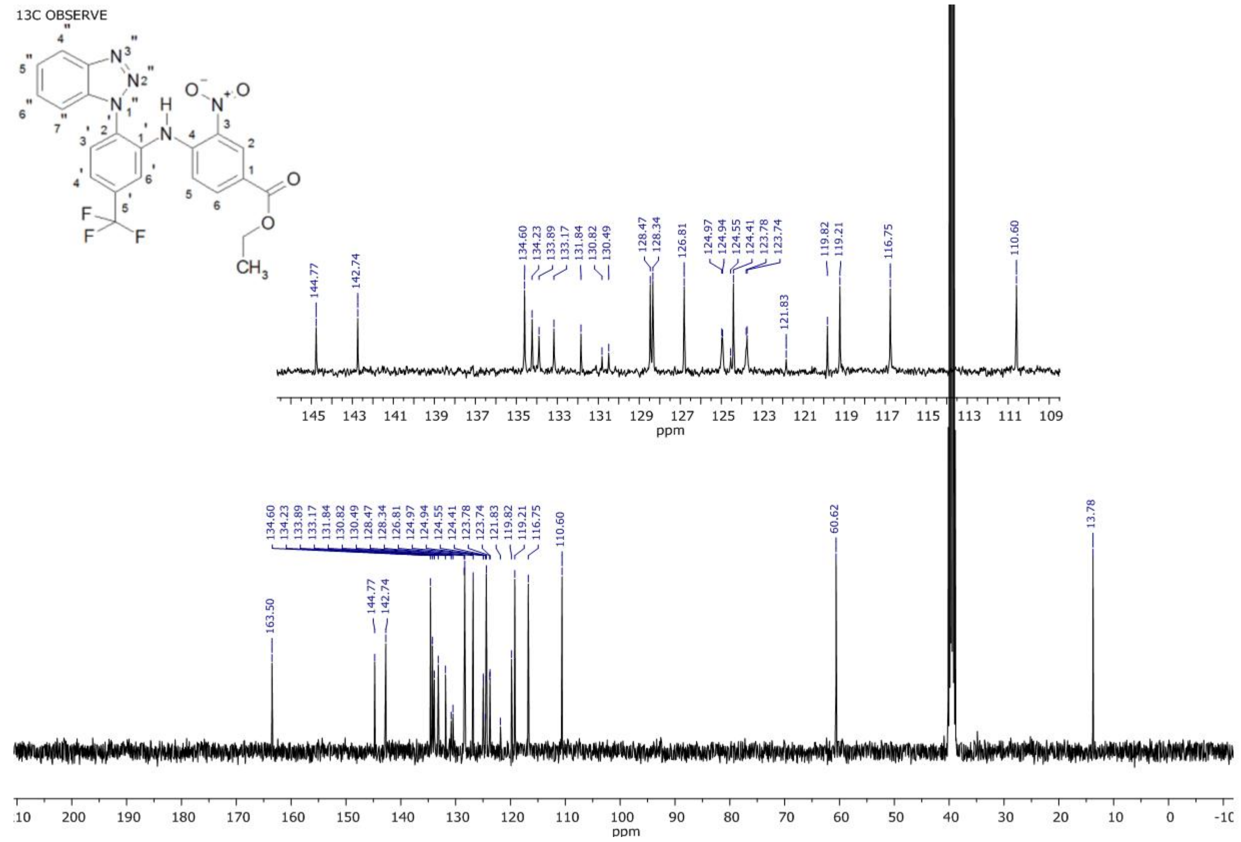

^{13}C NMR spectra of ethyl 4-[2-(1*H*-benzotriazol-1-yl)-5-(trifluoromethyl)anilino]-3-nitrobenzoate (**5c**) (DMSO-d_6)

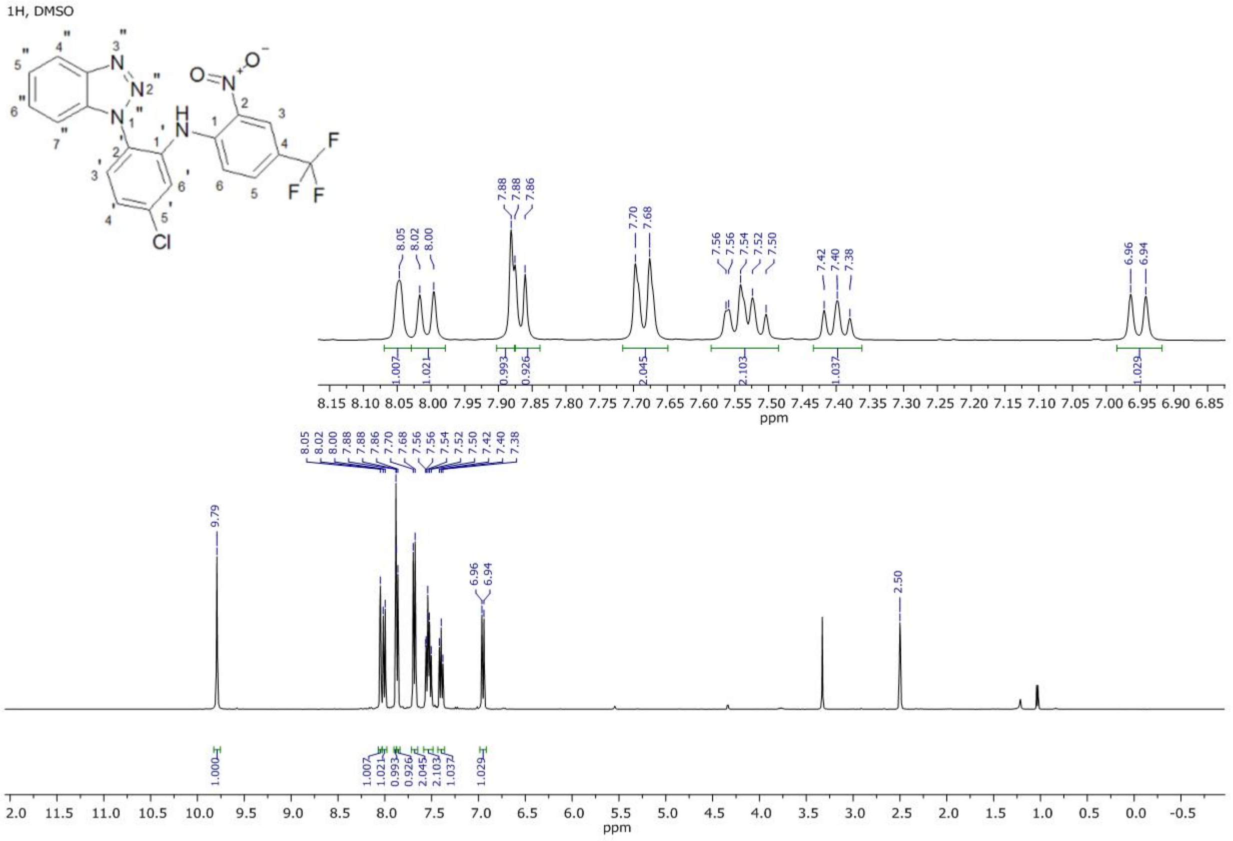

^{1}H NMR spectra of *N*-[2-(1*H*-benzotriazol-1-yl)-5-chlorophenyl]-2-nitro-4-(trifluoromethyl)aniline (**5d**) (DMSO-d_6)

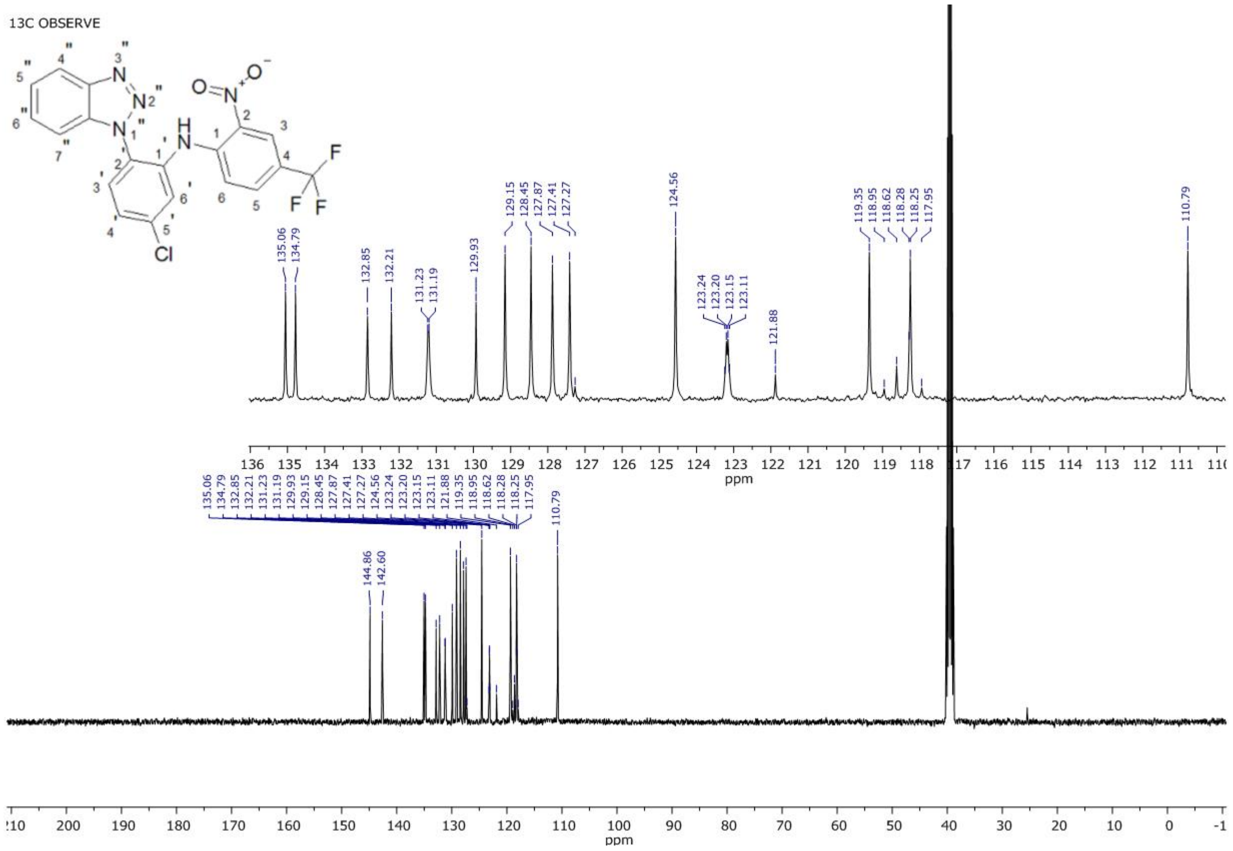

^{13}C NMR spectra of *N*-[2-(1*H*-benzotriazol-1-yl)-5-chlorophenyl]-2-nitro-4-(trifluoromethyl)aniline (**5d**) (DMSO-d_6)

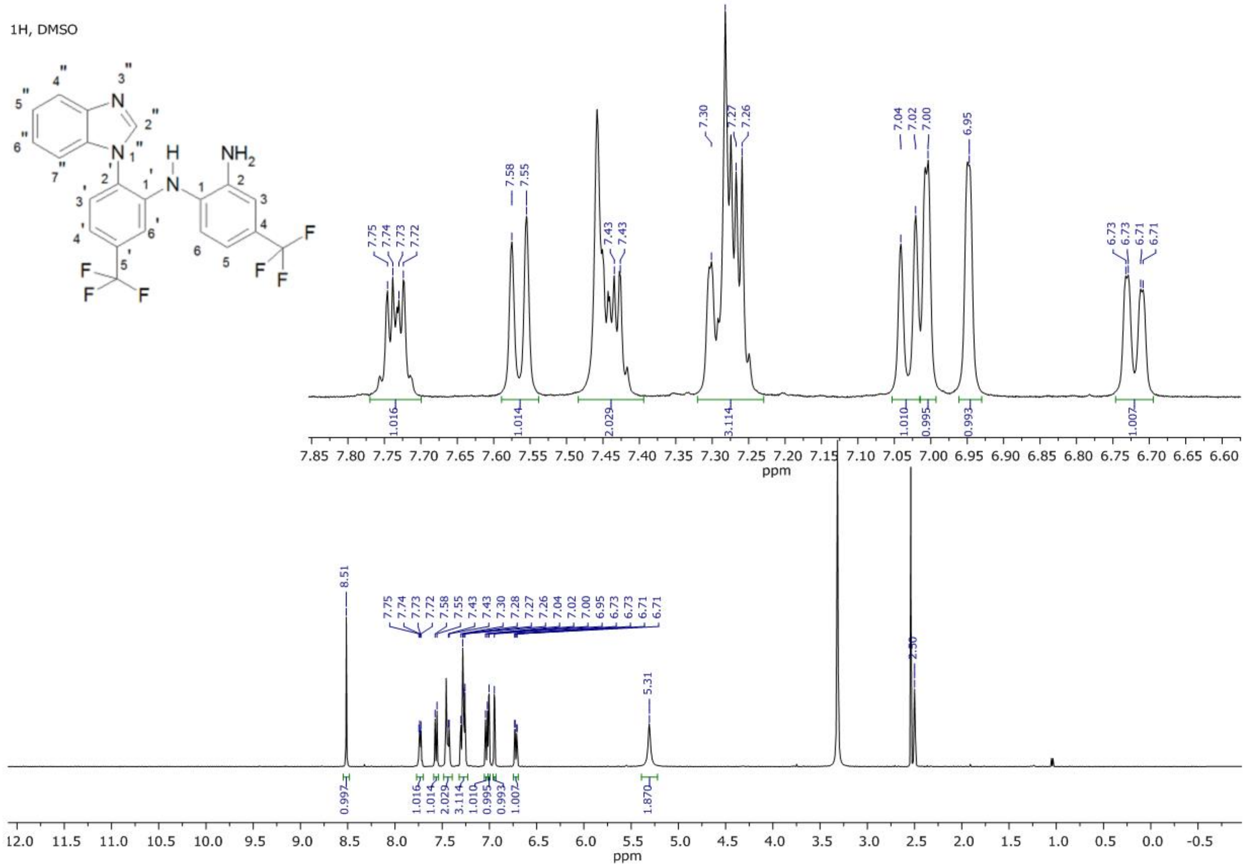

^{1}H NMR spectra of *N*-[2-(1*H*-benzimidazol-1-yl)-5-(trifluoromethyl)phenyl]-2-amino-4-(trifluoromethyl)aniline (**6a**) (DMSO-d_6)

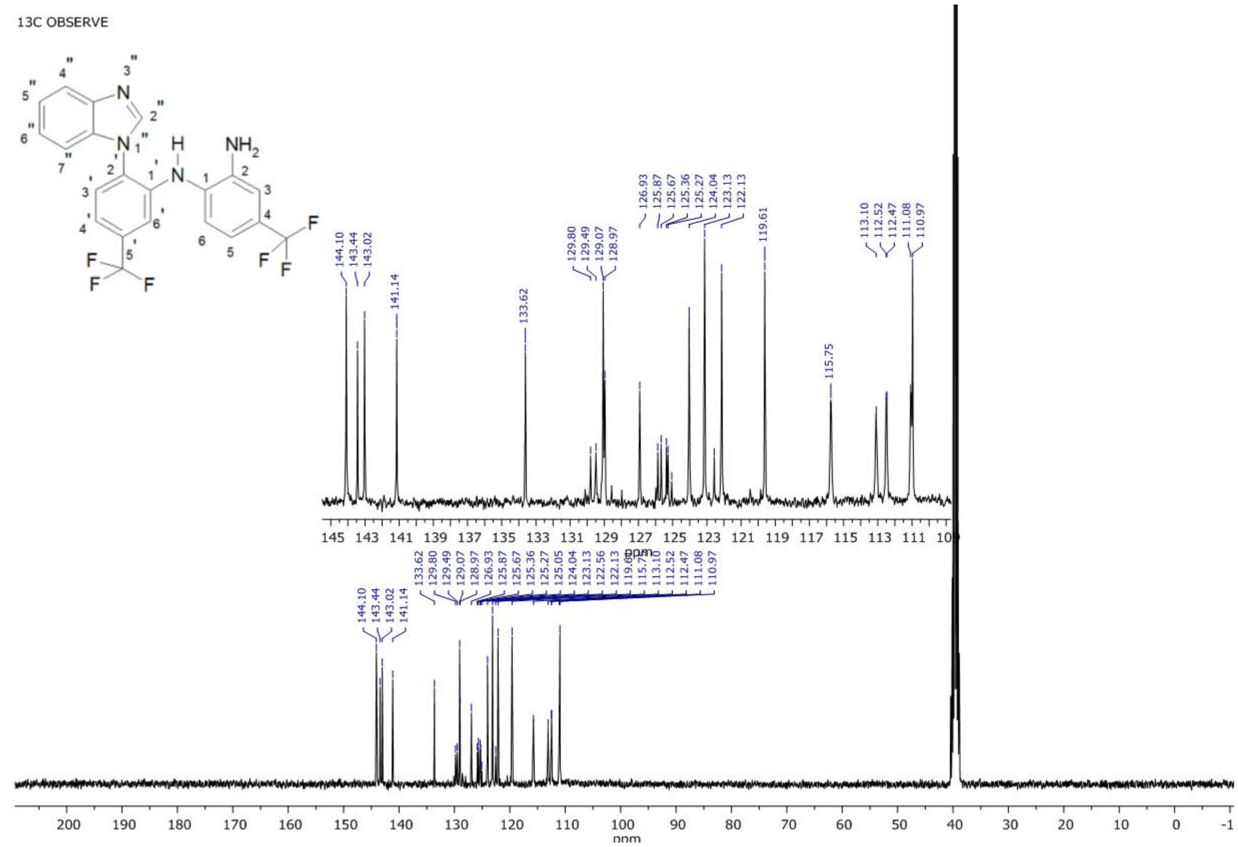

^{13}C NMR spectra of *N*-[2-(1*H*-benzimidazol-1-yl)-5-(trifluoromethyl)phenyl]-2-amino-4-(trifluoromethyl)aniline (**6a**) (DMSO-d_6)

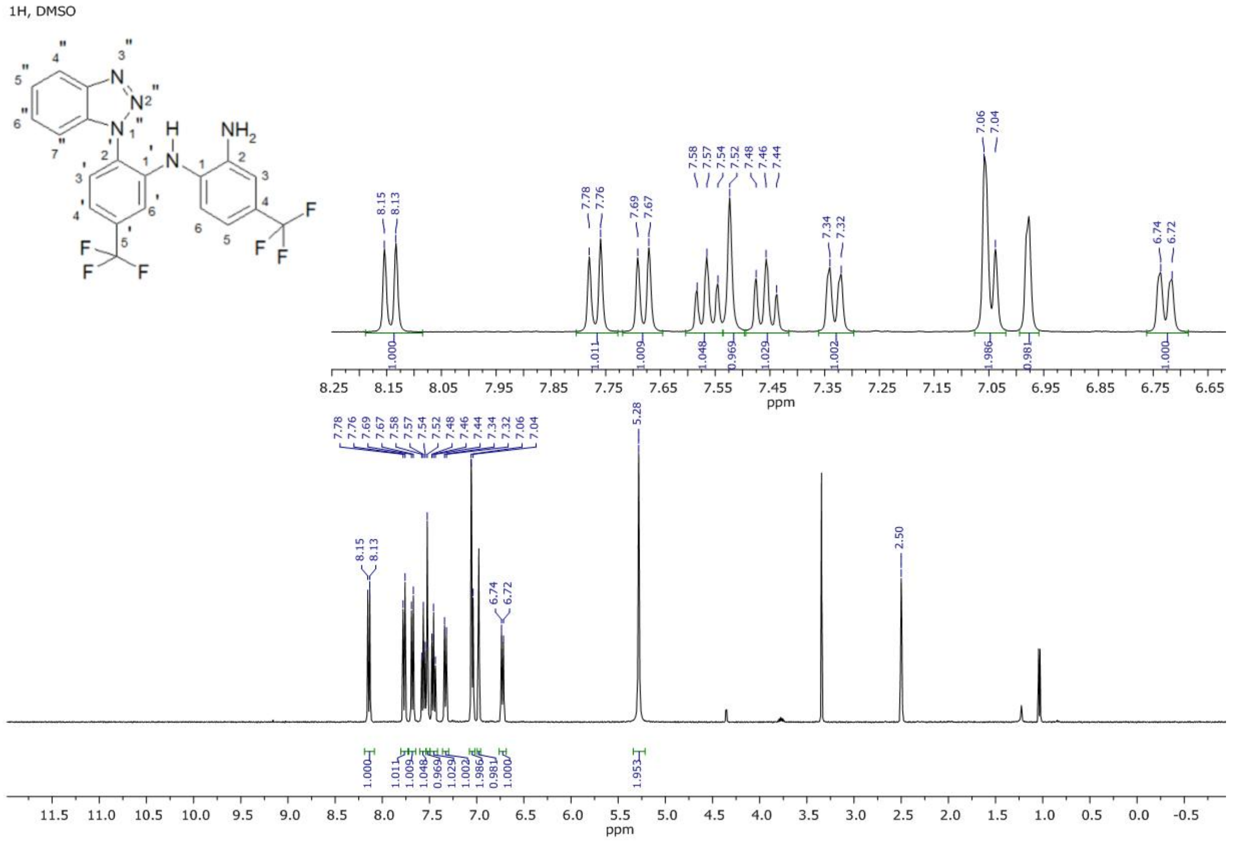

^{1}H NMR spectra of *N*-[2-(1*H*-benzotriazol-1-yl)-5-(trifluoromethyl)phenyl]-2-amino-4-(trifluoromethyl)aniline (**6b**) (DMSO-d_6)

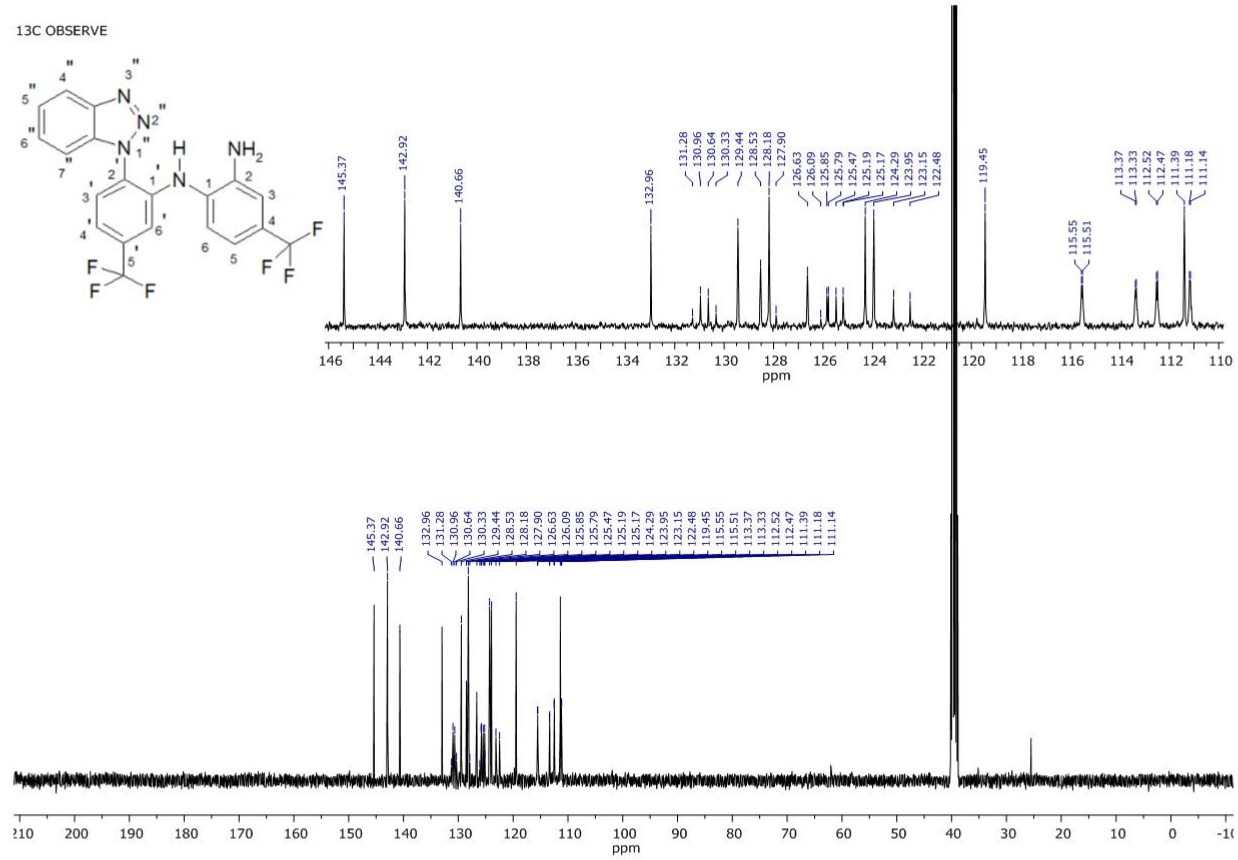

^{13}C NMR spectra of *N*-[2-(1*H*-benzotriazol-1-yl)-5-(trifluoromethyl)phenyl]-2-amino-4-(trifluoromethyl)aniline (**6b**) (DMSO-d_6)

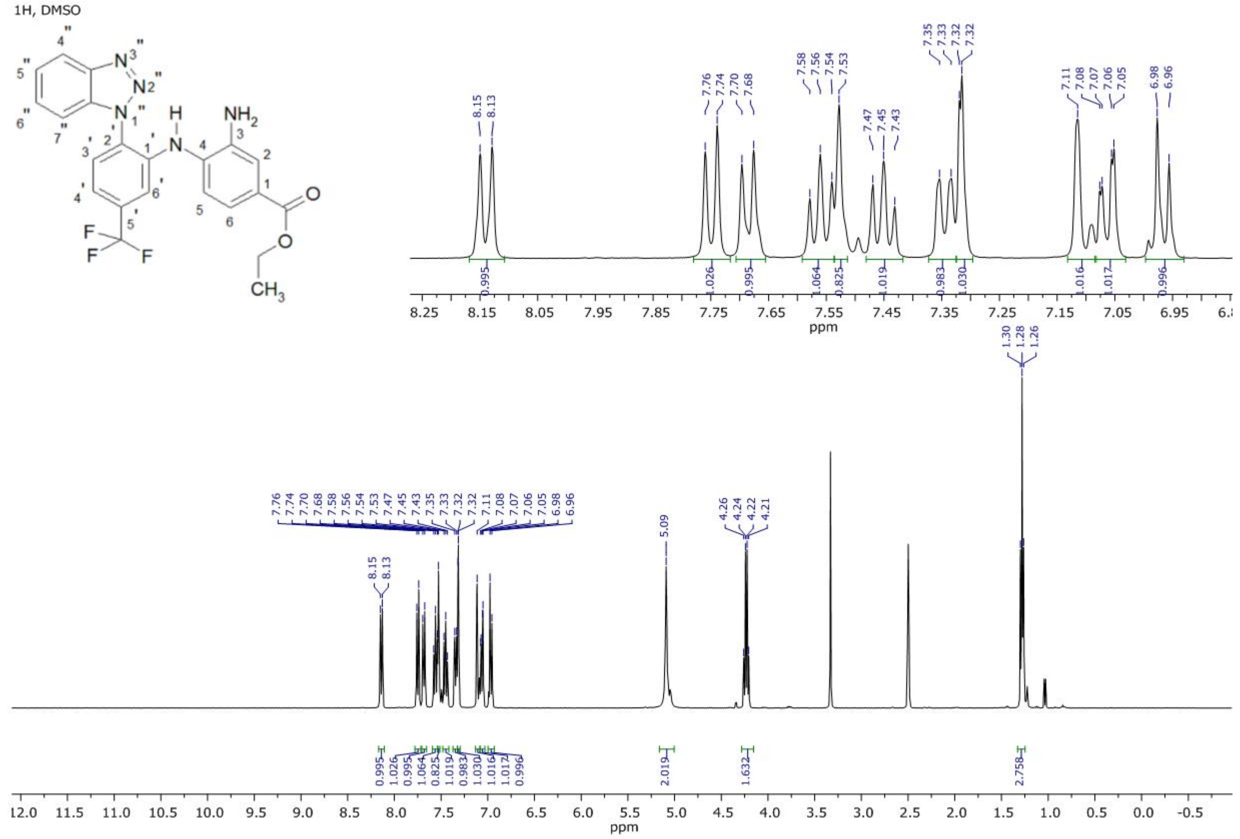

^{1}H NMR spectra of ethyl 3-amino-4-[2-(1*H*-benzotriazol-1-yl)-5-(trifluoromethyl)anilino]-benzoate (**6c**) (DMSO-d_6)

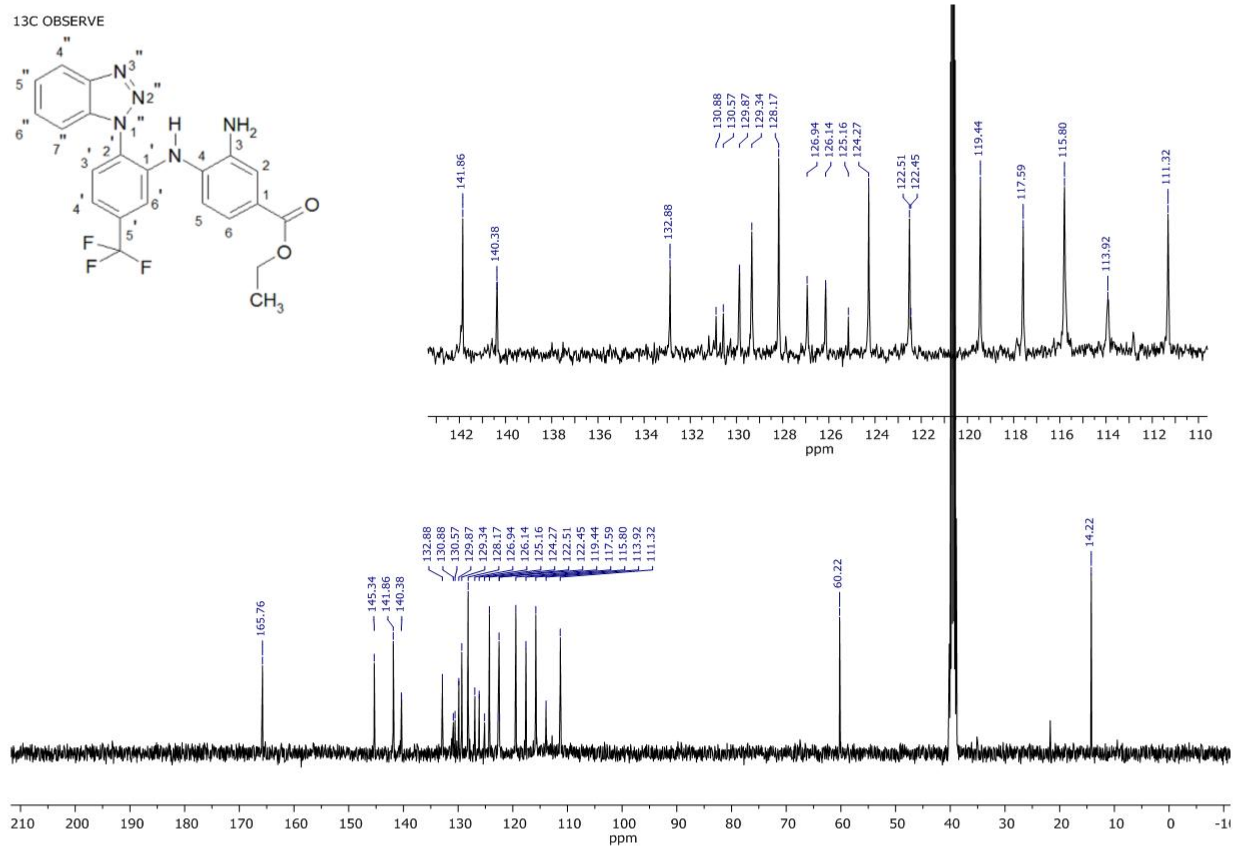

^{13}C NMR spectra of ethyl 3-amino-4-[2-(1*H*-benzotriazol-1-yl)-5-(trifluoromethyl)anilino]-benzoate (**6c**) (DMSO-d_6)

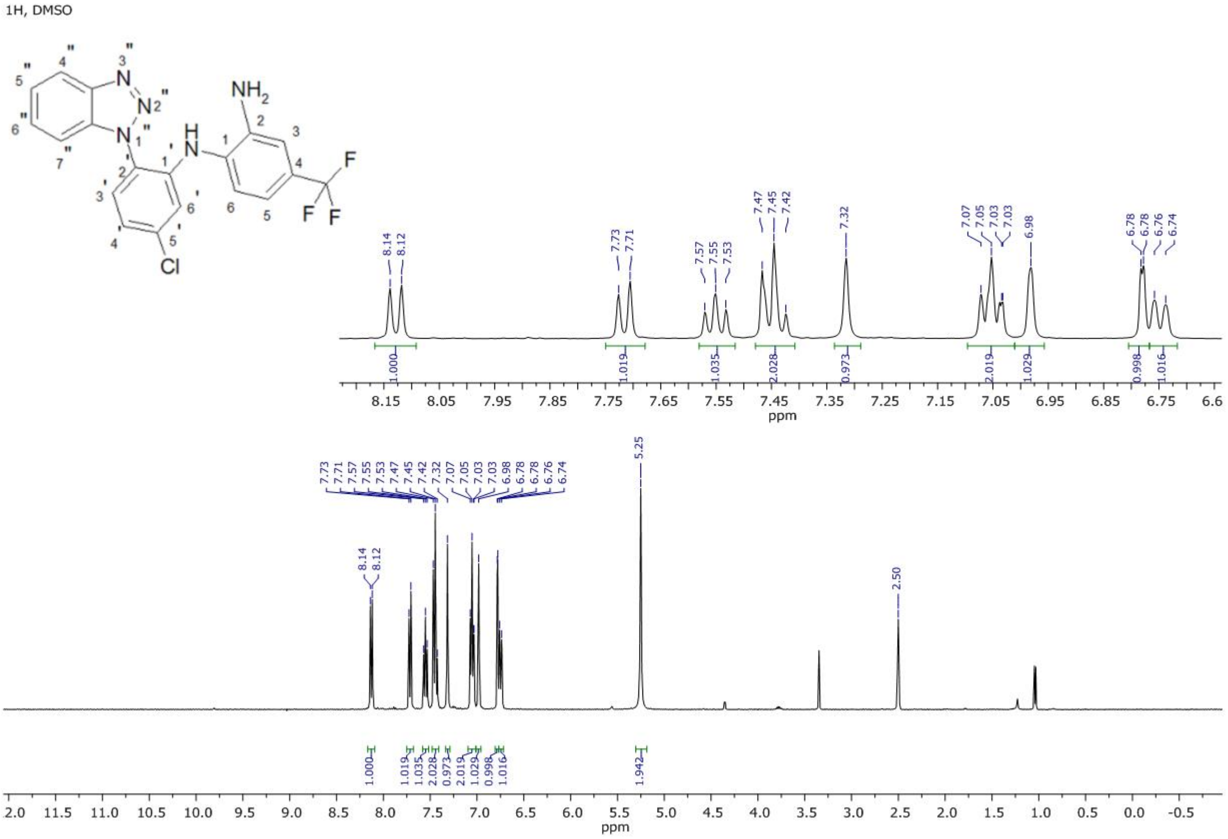

^{1}H NMR spectra of *N*-[2-(1*H*-benzotriazol-1-yl)-5-chlorophenyl]-2-amino-4-(trifluoromethyl)aniline (**6d**) (DMSO-d_6)

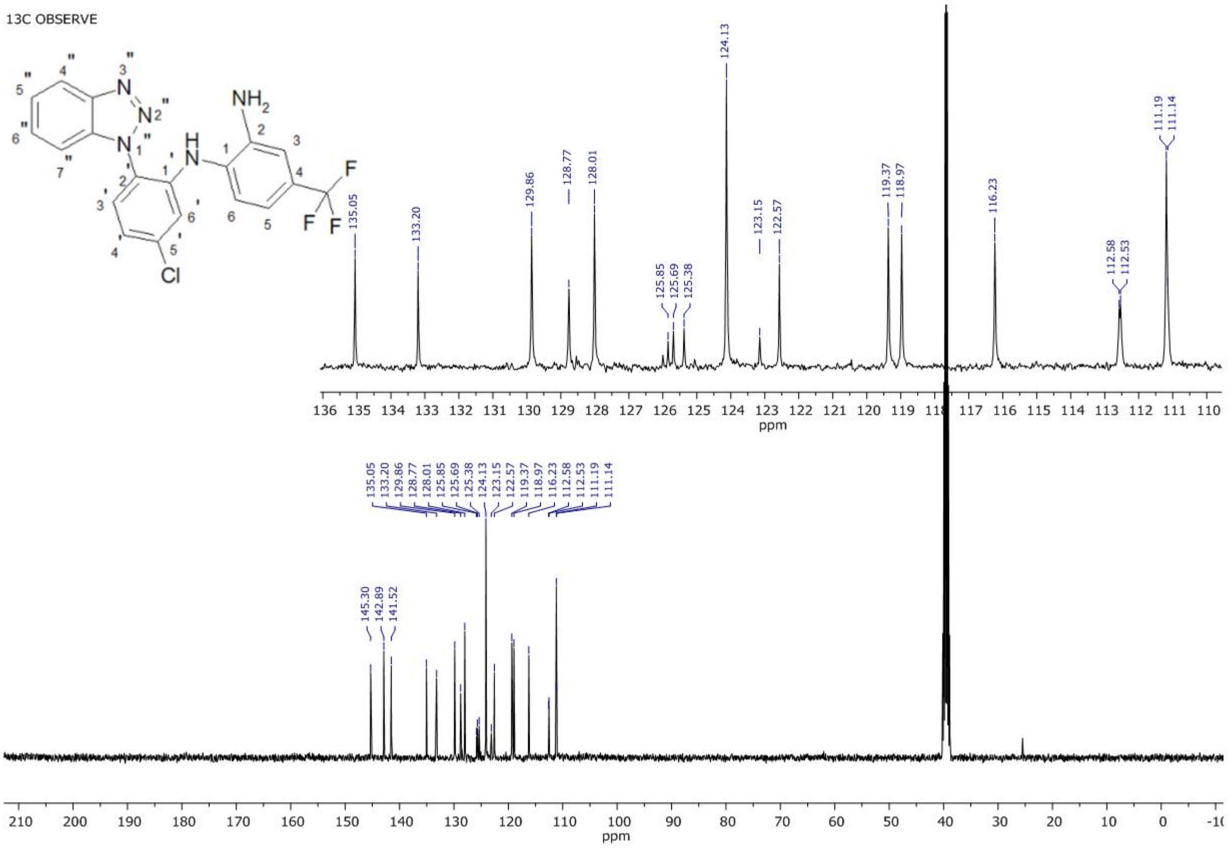

^{13}C NMR spectra of *N*-[2-(1*H*-benzotriazol-1-yl)-5-chlorophenyl]-2-amino-4-(trifluoromethyl)aniline (**6d**) (DMSO-d_6)

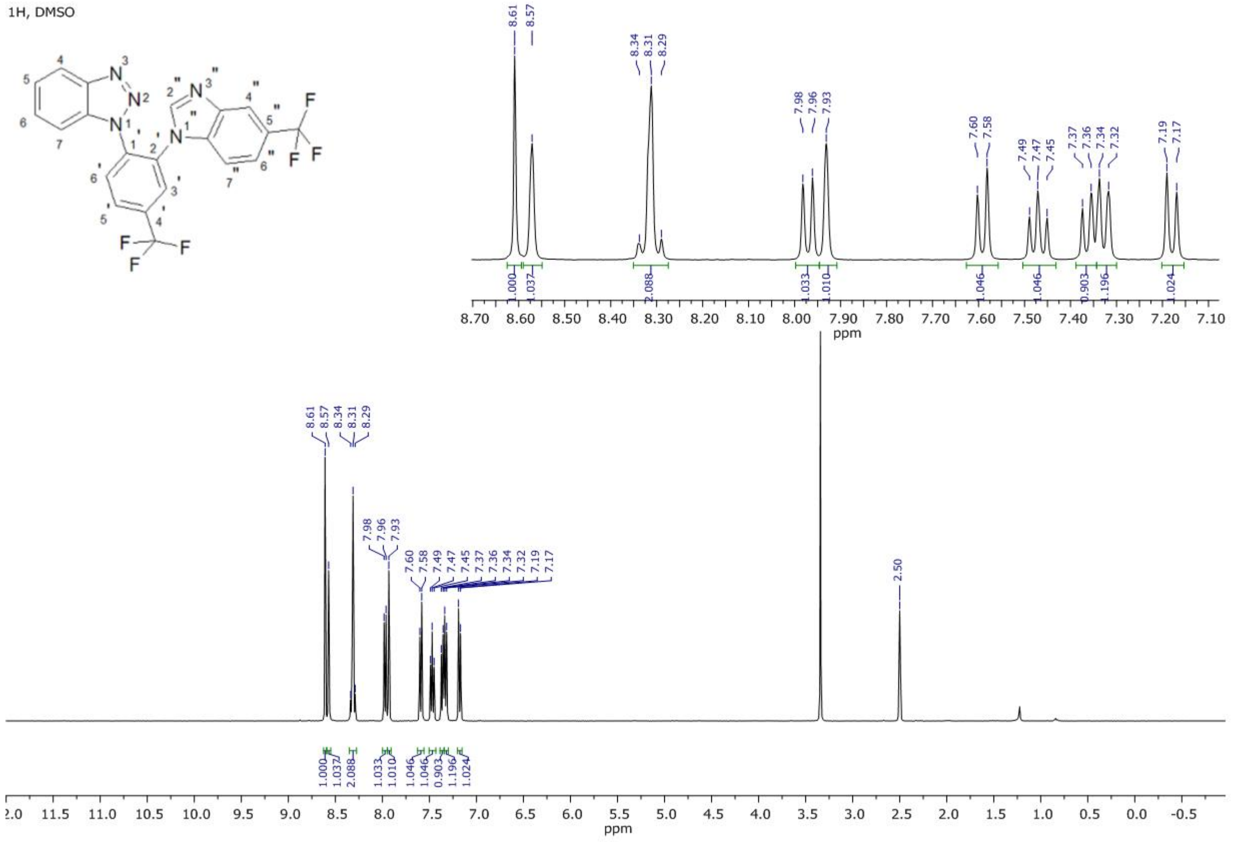

^{1}H NMR spectra of 1-{4-(trifluoromethyl)-2-[5-(trifluoromethyl)-1*H*-benzimidazol-1-yl]phenyl}-1*H*-benzotriazole (**7a**) (DMSO-d_6)

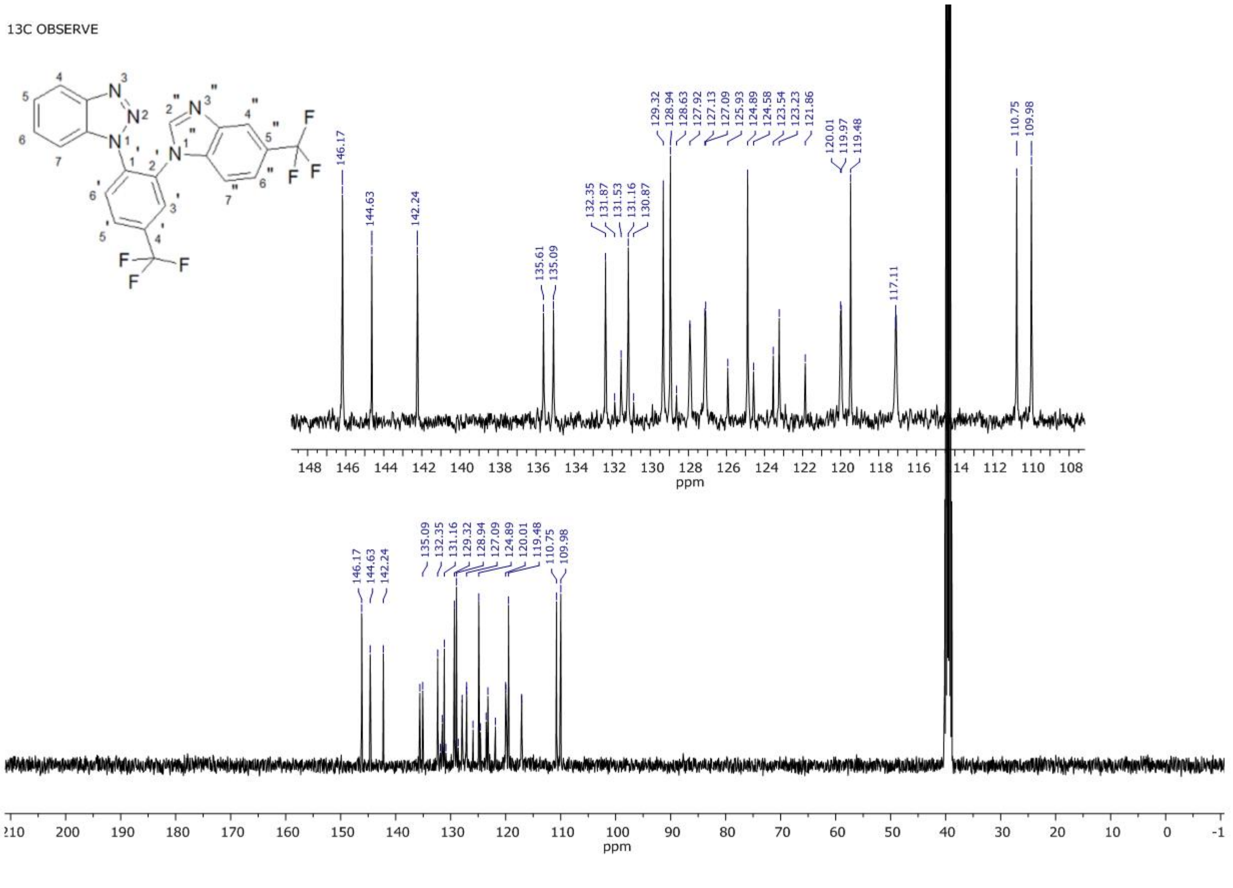

^{13}C NMR spectra of 1-{4-(trifluoromethyl)-2-[5-(trifluoromethyl)-1*H*-benzimidazol-1-yl]phenyl}-1*H*-benzotriazole (**7a**) (DMSO-d_6)

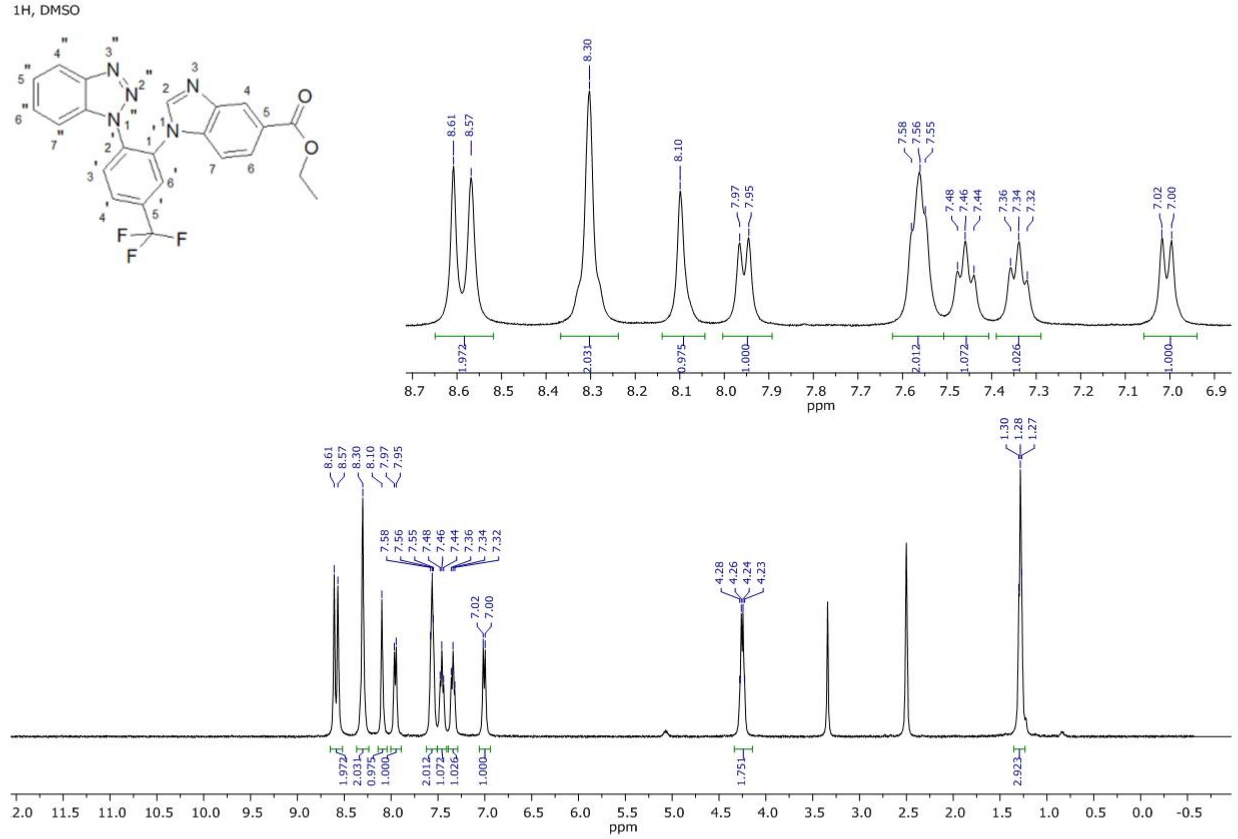

^{1}H NMR spectra of ethyl 1-[2-(1*H*-benzotriazol-1-yl)-5-(trifluoromethyl)phenyl]-1*H*-benzimidazole-5-carboxylate (**7b**) (DMSO-d_6)

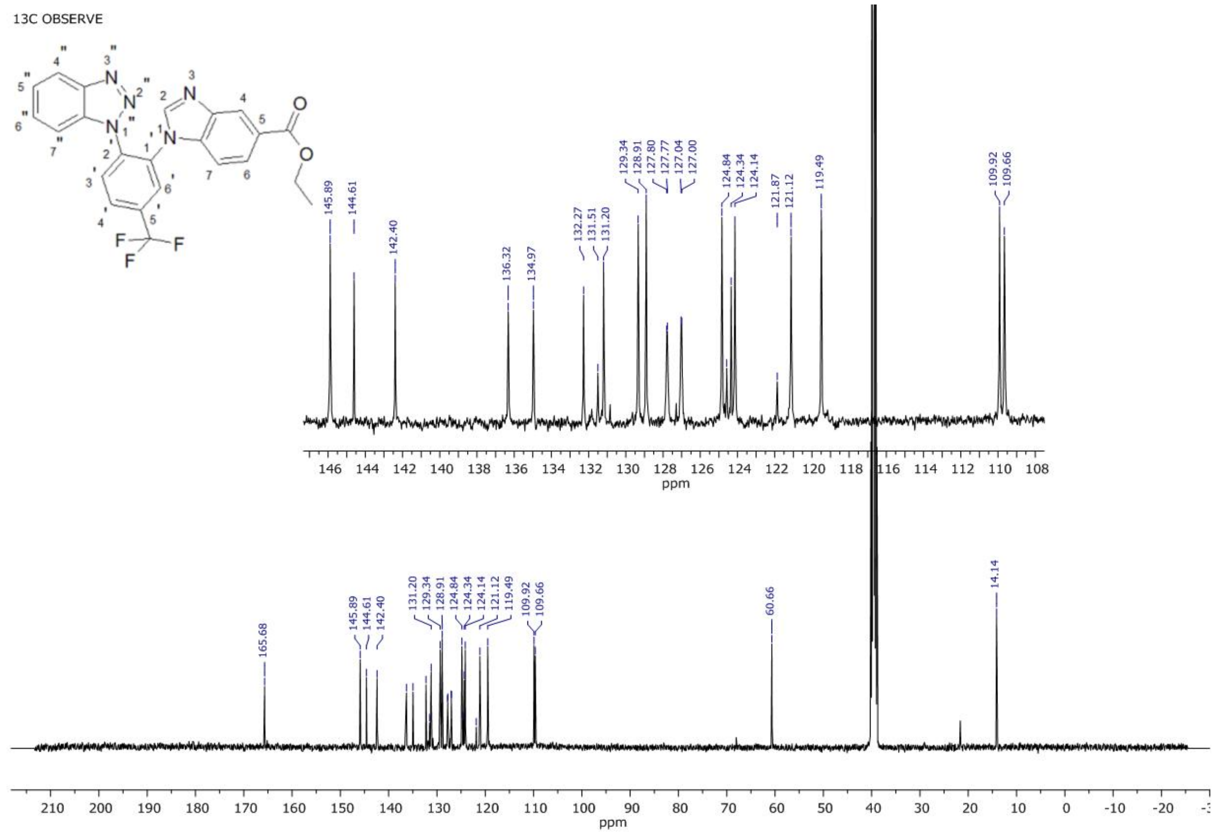

^{13}C NMR spectra of ethyl 1-[2-(1*H*-benzotriazol-1-yl)-5-(trifluoromethyl)phenyl]-1*H*-benzimidazole-5-carboxylate (**7b**) (DMSO-d_6)

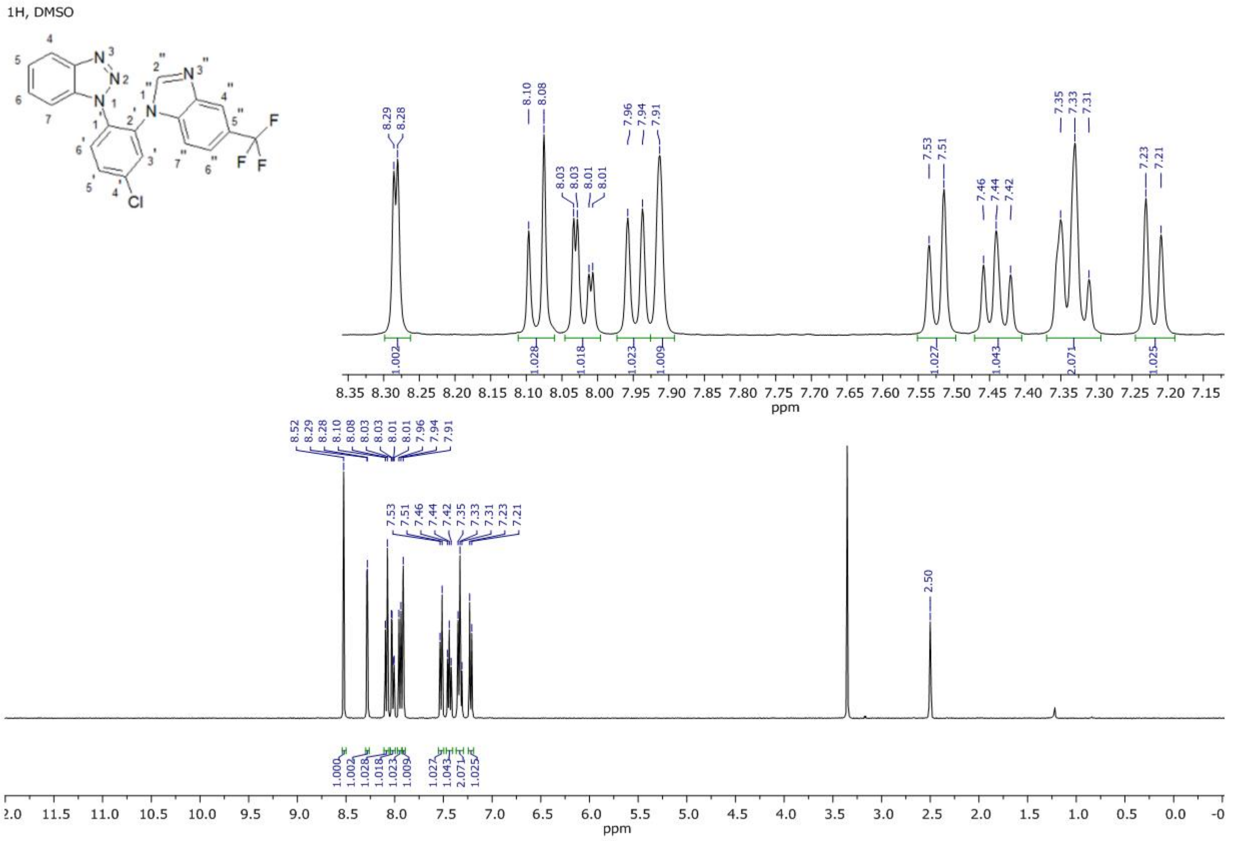

^{1}H NMR spectra of 1-{4-chloro-2-[5-(trifluoromethyl)-1*H*-benzimidazol-1-yl]phenyl}-1*H*-benzotriazole (**7c**) (DMSO-d_6)

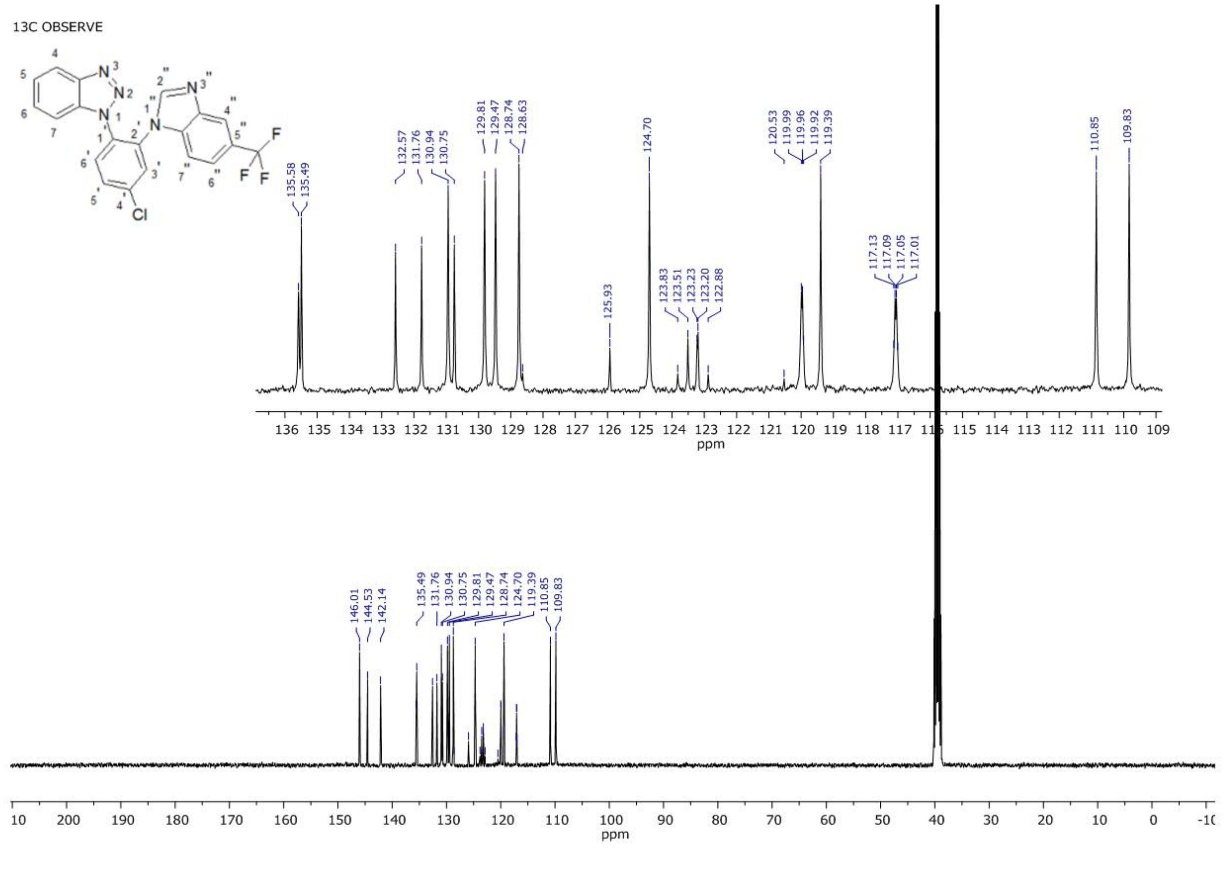

^{13}C NMR spectra of 1-{4-chloro-2-[5-(trifluoromethyl)-1*H*-benzimidazol-1-yl]phenyl}-1*H*-benzotriazole (**7c**) (DMSO-d_6)

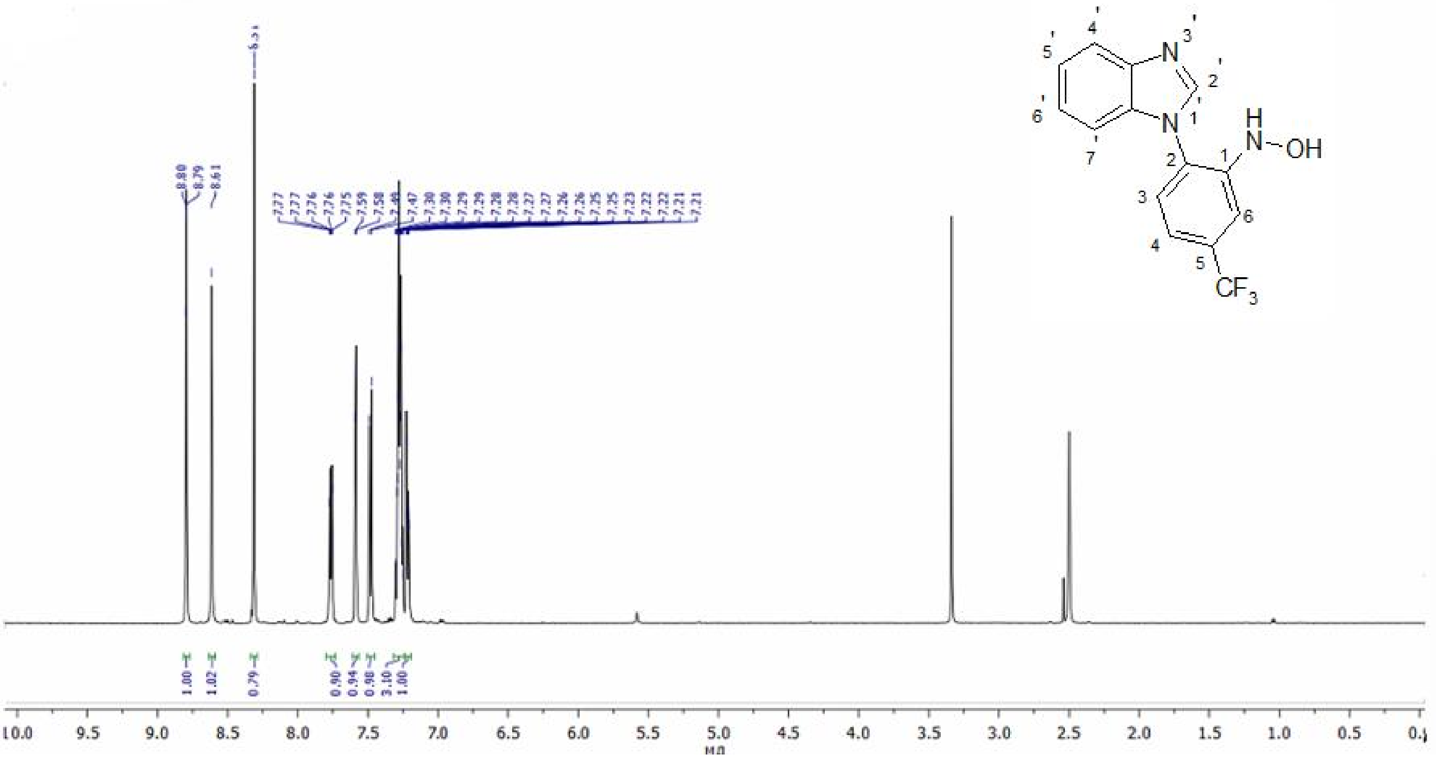

^{1}H NMR spectra of 1 N-(2-(1*H*-benzimidazol-1-yl)-5-(trifluoromethyl)phenyl)hydroxylamine (**8a**) (DMSO-d_6)

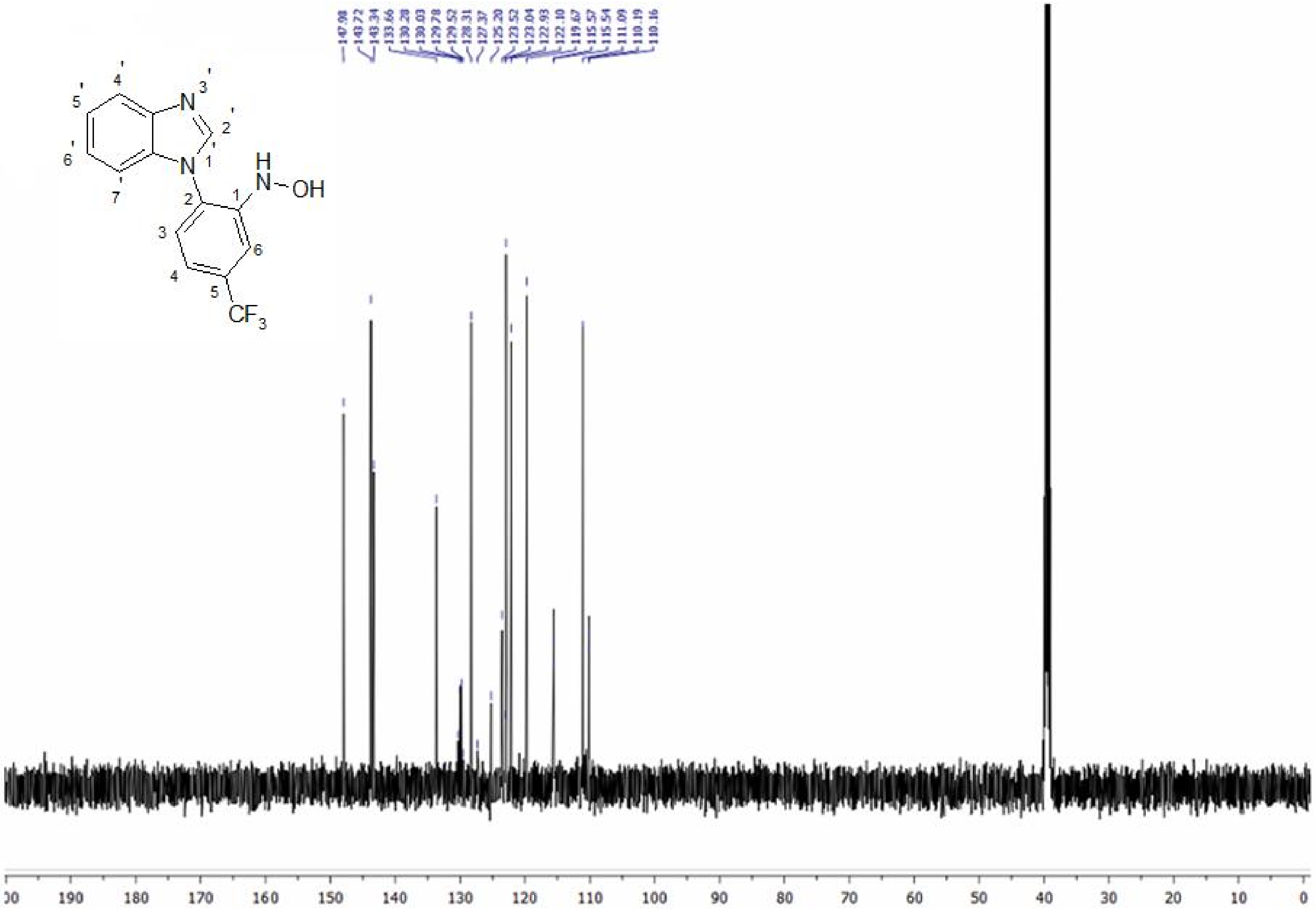

^{13}C NMR spectra of 1 N-(2-(1*H*-benzimidazol-1-yl)-5-(trifluoromethyl)phenyl)hydroxylamine (**8a**) (DMSO-d_6)

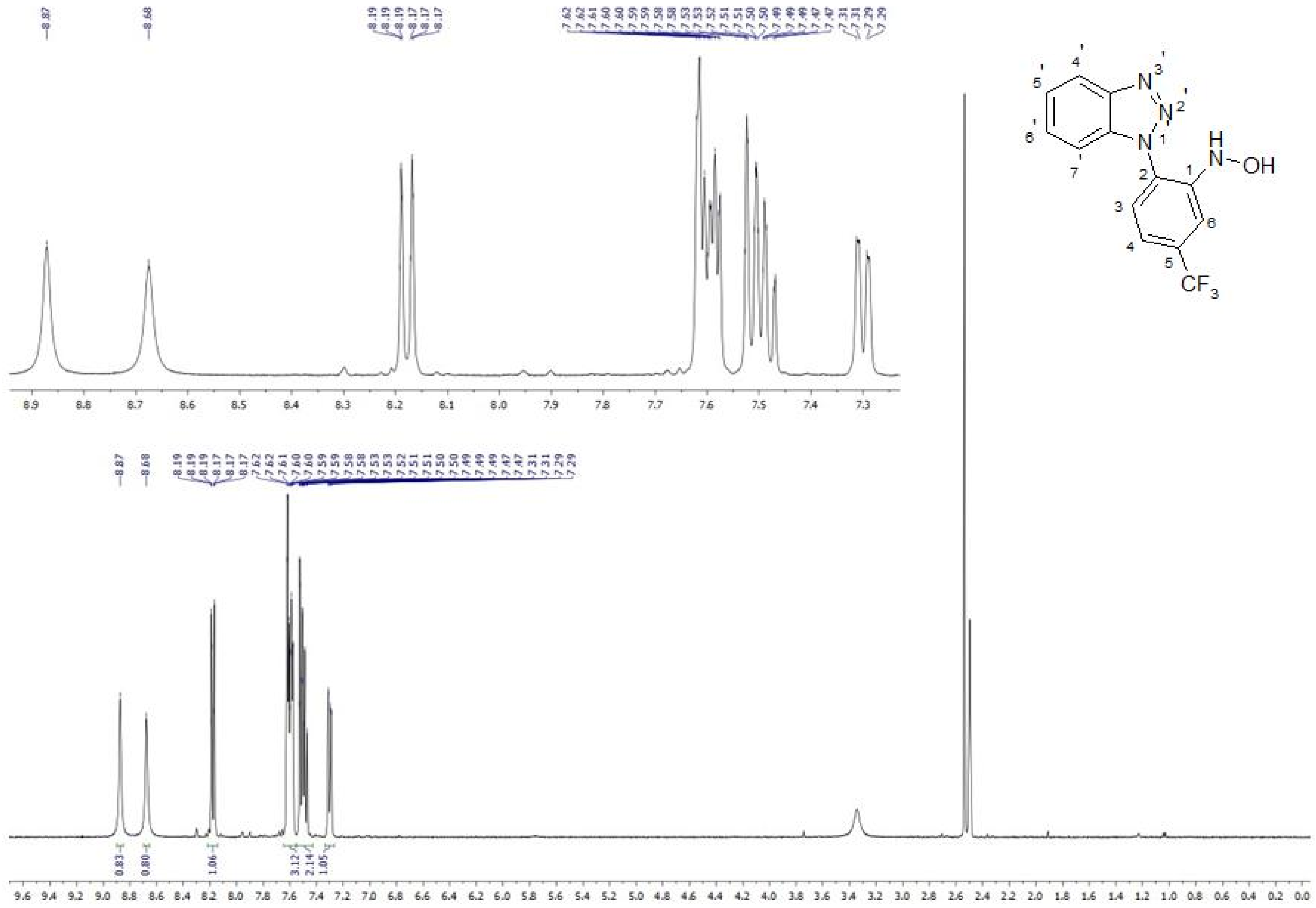

^{1}H NMR spectra of 1 N-(2-(1*H*-benzotriazol-1-yl)-5-(trifluoromethyl)phenyl)hydroxylamine (**8b**) (DMSO-d_6)

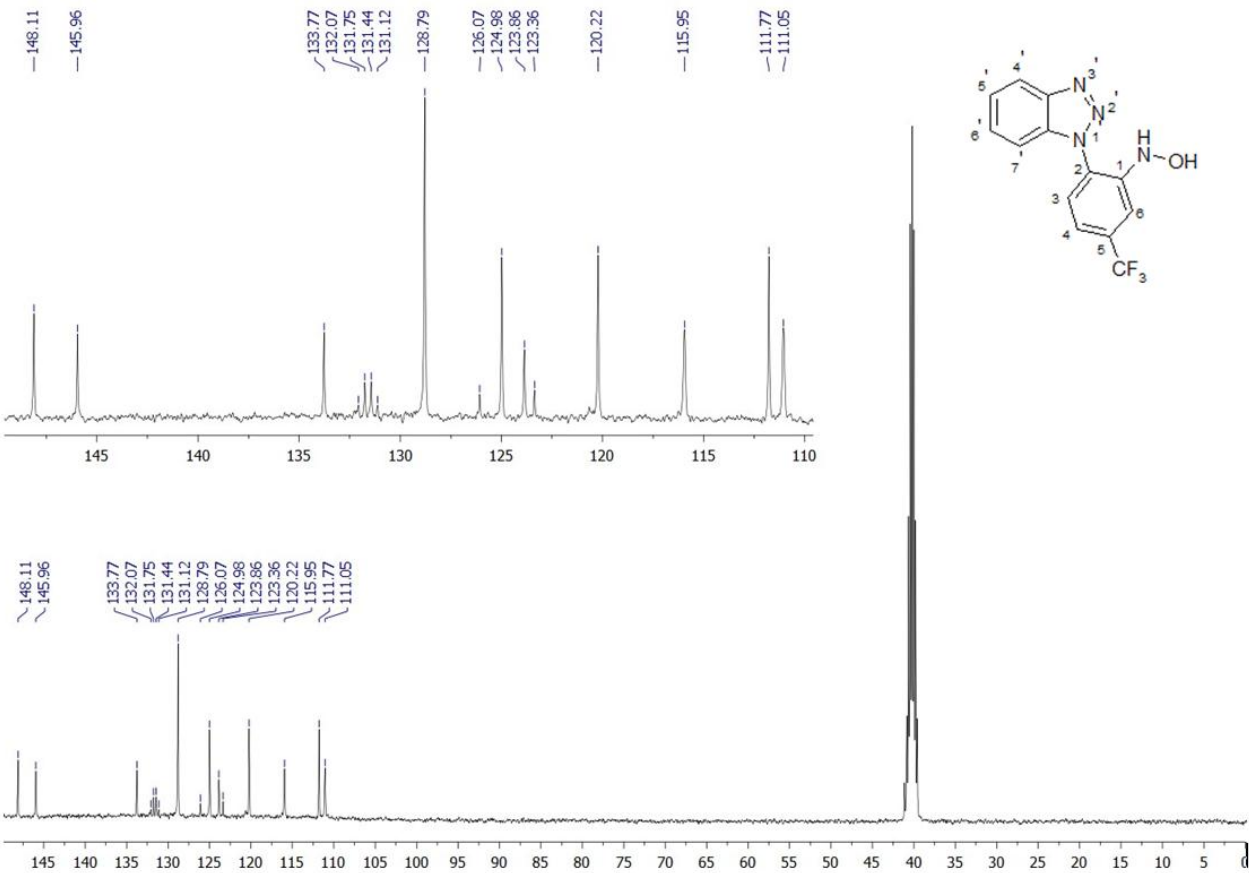

^{13}C NMR spectra of 1 N-(2-(1*H*-benzotriazol-1-yl)-5-(trifluoromethyl)phenyl)hydroxylamine (**8b**) (DMSO-d_6)
